# Structure of the intergenic spacers in chicken ribosomal DNA

**DOI:** 10.1186/s12711-019-0501-7

**Published:** 2019-10-26

**Authors:** Alexander Dyomin, Svetlana Galkina, Valerie Fillon, Stephane Cauet, Celine Lopez-Roques, Nathalie Rodde, Christophe Klopp, Alain Vignal, Anastasia Sokolovskaya, Alsu Saifitdinova, Elena Gaginskaya

**Affiliations:** 1 Saint Petersburg State University, Universitetskaya emb. 7/9, Saint Petersburg, 199034 Russian Federation; 2 Saratov State Medical University, Bolshaya Kazachia Str. 112, Saratov, Russian Federation; 3 INRA, GenPhySE, 24 Chemin de Borde Rouge, Auzeville, 31326 Castanet Tolosan, France; 4 INRA, CNRGV, 24 Chemin de Borde Rouge, Auzeville, 31326 Castanet Tolosan, France; 5 INRA, GeT-PlaGe, 24 Chemin de Borde Rouge, Auzeville, 31326 Castanet Tolosan, France; 6 INRA, Sigenae, MIAT, 24 Chemin de Borde Rouge, Auzeville, 31326 Castanet Tolosan, France; 7 Herzen State Pedagogical University of Russia, Moika Emb. 48, Saint Petersburg, 191186 Russian Federation

## Abstract

**Background:**

Ribosomal DNA (rDNA) repeats are situated in the nucleolus organizer regions (NOR) of chromosomes and transcribed into rRNA for ribosome biogenesis. Thus, they are an essential component of eukaryotic genomes. rDNA repeat units consist of rRNA gene clusters that are transcribed into single pre-rRNA molecules, each separated by intergenic spacers (IGS) that contain regulatory elements for rRNA gene cluster transcription. Because of their high repeat content, rDNA sequences are usually absent from genome assemblies. In this work, we used the long-read sequencing technology to describe the chicken IGS and fill the knowledge gap on rDNA sequences of one of the key domesticated animals.

**Methods:**

We used the long-read PacBio RSII technique to sequence the BAC clone WAG137G04 (Wageningen BAC library) known to contain chicken NOR elements and the HGAP workflow software suit to assemble the PacBio RSII reads. Whole-genome sequence contigs homologous to the chicken rDNA repetitive unit were identified based on the *Gallus_gallus*-5.0 assembly with BLAST. We used the Geneious 9.0.5 and Mega software, maximum likelihood method and Chickspress project for sequence evolution analysis, phylogenetic tree construction and analysis of the raw transcriptome data.

**Results:**

Three complete IGS sequences in the White Leghorn chicken genome and one IGS sequence in the red junglefowl contig AADN04001305.1 (*Gallus_gallus*-5.0) were detected. They had various lengths and contained three groups of tandem repeats (some of them being very GC rich) that form highly organized arrays. Initiation and termination sites of rDNA transcription were located within small and large unique regions (SUR and LUR), respectively. No functionally significant sites were detected within the tandem repeat sequences.

**Conclusions:**

Due to the highly organized GC-rich repeats, the structure of the chicken IGS differs from that of IGS in human, apes, Xenopus or fish rDNA. However, the chicken IGS shares some molecular organization features with that of the turtles, which are other representatives of the Sauropsida clade that includes birds and reptiles. Our current results on the structure of chicken IGS together with the previously reported ribosomal gene cluster sequence provide sufficient data to consider that the complete chicken rDNA sequence is assembled with confidence in terms of molecular DNA organization.

## Background

Arrays of ribosomal DNA (rDNA) repeated units are extremely important components of eukaryotic genomes. They form nucleolus organizing regions (NOR) in one or several chromosome pairs. Active NOR build a nucleolus, which is a dynamic nuclear compartment involved in ribosome biogenesis. The functional state of the nucleolus is an indicator of the cell and tissue physiological states. Each rDNA unit consists of an rRNA gene cluster (5′ETS (external transcribed spacer), 18S rRNA, ITS1 (internal transcribed spacer), 5.8S rRNA, ITS2, 28S rRNA, 3′ETS) that is transcribed into a single pre-rRNA molecule and an intergenic spacer (IGS). In spite of numerous animal genome-wide studies conducted recently, the structural organization of the entire rDNA unit sequence, particularly the IGS, remains poorly investigated for most vertebrates. Nevertheless, in the species that have been studied, the IGS was found to play a key role in pre-rRNA cluster transcription regulation. In particular, it contains regulatory sites for RNA polymerase I (RNApol I). Transcription termination sites for the upstream rRNA gene cluster are located at the IGS 5′ end and the transcription initiation site of the downstream cluster at the IGS 3′ end [1]. In mammals, additional RNApol I promoters are located within the IGS, at least 2 kb upstream of the pre-rRNA start site, and spacer promoter transcripts are assumed to have a function in the NoRC (nucleolar chromatin-remodelling complex) directed transcriptional silencing of rDNA [2, 3]. Replication origins and replication fork barriers impede the entry of the replication machinery into the transcription unit [4–6]. Certain regulatory elements in IGS are conserved at least between primate species [7]. A typical IGS includes specific repeats that vary in copy number, which causes heterogeneity in spacer length. They can serve as transcription terminator elements or have an enhancer activity [1, 8–12]. Progress in long-read sequencing methods makes it possible to capture entire rRNA repetitive units within individual reads and to establish their detailed structure, and thereby to unmask within-individual variability in humans [13].

The chicken (*Gallus gallus*) genome includes a single NOR, which maps to chromosome 16 and contains the rDNA array [14–17]. As for many other eukaryote species, the chicken rDNA array is absent from the current version of the assembled GRCg6a chicken genome (https://www.ncbi.nlm.nih.gov/assembly/GCF_000002315.5/). Nevertheless, the composition of the chicken rDNA array, in particular the IGS, has been investigated in earlier works based on rDNA restriction analysis and sequencing of fragments. By analyzing rDNA repeat units in various domestic chicken lines, Delany and coworkers [18–20] established that the intra- and inter-NOR variability of the length of rDNA repeat units ranged from 11 to 50 kb and was mainly due to heterogeneity in IGS size. The overall 5 to 7 Mb variation in NOR lengths was assumed to depend on the heterogeneity of IGS size and variation in rDNA unit copy number. Delany and Krupkin established that the average number of rDNA repeat copies in diploid sets ranged from 279 to 368 [20]. A spacer promoter (9 bp) and the following RNApol I transcription start site (10 bp) were sequenced from a cloned fragment of chicken IGS [21]. The question of whether the function of the spacer promoter is related to the initiation of rRNA gene cluster transcription or serves to generate a regulatory RNA for NoRC dependent rDNA transcriptional silencing, as in mammals, has not been explored so far in other vertebrates. Undoubtedly, to date, the lack of available data on complete IGS sequences has prevented the study of the regulatory mechanisms of NOR.

Our earlier attempt to assemble a complete chicken rDNA repeat unit (rRNA gene cluster plus IGS) using Illumina sequencing data was unsuccessful due to the complex structure of the repeats within the IGS, and only the transcribed part of the rRNA gene cluster was determined (NCBI Nucleotide: KT445934; [22]). To date, the description of the complete rDNA repeat unit sequences is not available for any representative of the Sauropsida clade that includes reptiles and birds, although such data are essential to study regulatory molecular interactions and evolutionary mechanisms in this group.

Here, we describe the chicken IGS structure based on the PacBio single-molecule sequencing of a BAC clone containing a chicken NOR fragment that includes three complete IGS. We identified several novel tandem repeats in the chicken IGS, which form highly organized structures. The number of repeat variants is indicative of IGS heterogeneity within the chicken rDNA sequence.

## Methods

### BAC clone sequencing and assembling

We selected the WAG137G04 BAC clone from the Wageningen BAC library [23], which was constructed with DNA from a female White Leghorn chicken, for long-read PacBio RSII sequencing. This BAC clone is known to contain NOR elements [17, 22]. Library preparation and sequencing were performed at the GeT-PlaGe core facility, INRA Toulouse, according to the manufacturer’s instructions “Shared protocol-20 kb Template Preparation Using BluePippin Size Selection system (15 kb size Cutoff)”. At each step, DNA was quantified using the Qubit dsDNA HS Assay Kit (Life Technologies). DNA concentration and purity were measured using a NanoDrop™ spectrophotometer (Thermofisher) and size distribution and degradation were assessed using the Fragment analyzer (AATI) High Sensitivity Large Fragment 50 kb Analysis Kit. Purification steps were performed using 0.45X AMPure PB beads (PacBio).

High-quality BAC DNA was extracted using the Nucleobond Xtra Midi Plus kit (Macherey–Nagel) following the manufacturer’s instructions using 100 mL of LB media that contains chloramphenicol to select clones (12.5 µg/mL). DNA damage repair and end repair were performed with the SMRTBell template Prep Kit 1.0 (PacBio). After ligation to blunt hairpin adapters, the library was treated with an exonuclease cocktail to digest unligated DNA fragments. A 10-kb cutoff size selection step was performed with the BluePippin Size Selection system (Sage Science) on 0.75% agarose cassettes, Marker S1 high Pass 15-20 kb.

Conditioned Sequencing Primer V2 was annealed to the size-selected SMRTbell templates, which were then bound to polymerase P6-C4 with a polymerase: SMRTbell template ratio of 10:1. After performing a magnetic bead-loading step (OCPW), the SMRTbell library was sequenced on one SMRTcell (RSII instrument at 0.25 nM with a 360 min movie). In fact, the WAG137G04 BAC clone DNA was sequenced in a pool with 12 other BAC clones. We obtained a mean read length of 9 kb for the pool of sequences and 7168 reads were obtained for the WAG137G04 BAC (NCBI SRA: PRJNA577229). The quality value was 48 as scored by the PacBio SMRT^®^ Analysis software suite that calculates this value after the assembly step and predicts the error probability of a basecall, based on Phred quality score.

### PacBio RS II assembly

PacBio RS II reads were assembled following the HGAP workflow (https://github.com/PacificBiosciences/Bioinformatics-Training/wiki/HGAP). The SMRT^®^ Analysis (v2.2.0) software suite was used for HGAP implementation. The longest contig WAG137G4_utg0 (100,614 bp) was obtained by the HGAP3 method with an average coverage of 500X. However, the coverage was not regular, with two regions of repeated sequences with a high coverage (see Additional file 1: Figure S1).

First, reads were aligned using BLASR against “*Escherichia coli* str. K12 substr. DH10B, complete genome”. The detected *E. coli* reads and low-quality reads (read quality < 0.75 and read length < 500 bp) were removed from the data. The filtered reads (8390 reads, 78,347,167 bp) were then preassembled to generate long and highly accurate sequences (776 reads, 5,031,668 bp). For this step, we separated the longest reads (> 13 kb) in order to correct read errors by mapping the first ones to the second ones. Then, the sequences were filtered against vector sequences, and the Celera assembler was used to assemble the data into a draft assembly. A final “polishing” step of the HGAP workflow with the Quiver algorithm, which is a quality-aware consensus algorithm that uses the rich quality scores embedded in Pacific Biosciences bas.h5 files, decreased significantly the remaining InDel (short insertions or deletions) and base substitution errors in the draft assembly.

### IGS analysis and annotation

Nucleotide sequence alignment, identification and annotation of IGS specific repeats in the chicken IGS sequence, analysis of the distribution of CpG islands and IGS transcription were carried out with the Geneious 9.0.5 software package (https://www.geneious.com/). WGS contigs that are homologous to the chicken rRNA repetitive unit were searched in the Gallus_gallus-5.0 assembly of the red junglefowl genome (NCBI Assembly: GCA_000002315.3, GCF_000002315.4) (NCBI WGS: AADN00000000.4) using BLAST (https://blast.ncbi.nlm.nih.gov/Blast.cgi). Homologous repetitive sequences were identified by NCBI BLAST search and Repbase Update library (https://www.girinst.org/repbase/) search.

### Phylogenetic analysis of IGS specific repeats

The Molecular Evolutionary Genetics Analysis (MEGA) version 7.0 software [24] was used for a statistical analysis of molecular evolution (see Additional file 2: Table S1 and Additional file 3: Table S2) and reconstruction of the genetic links between IGS specific repeats. A phylogenetic tree was constructed by using the maximum likelihood method combined with the evolutionary nucleotide replacement Kimura 2-parameter model with a Gamma distribution [5 categories (+G, parameter = 2.8698)] [24, 25]. To assess the reliability of the tree topology, we applied a Bootstrap test (500 replications).

### Transcriptome data analysis

To determine whether the IGS is transcribed in chicken, we analyzed raw transcriptomic data that were available from the Chickspress project (NCBI BioProject: PRJEB4677; PRJNA204941; http://geneatlas.arl.arizona.edu/), in which RNA libraries were created from red junglefowl total RNA extracted with the miRNeasy Mini Kit (Qiagen) [26]. We used the transcriptomes of chicken testis (NCBI SRA: ERX321399), ovary (NCBI SRA: ERX321403), kidney (NCBI SRA: ERX321415), liver (NCBI SRA: ERX321417) and heart (NCBI SRA: ERX321421). The IGS sequence (15,241 bp) flanked by the 3′ETS (335 bp) and 5′ETS (1836 bp) sequences from the WAG137G4_utg0 contig was used as a reference.

## Results

### Assembly of the rRNA gene cluster based on sequencing data from the WAG137G04 BAC clone

**Fig. 1:**
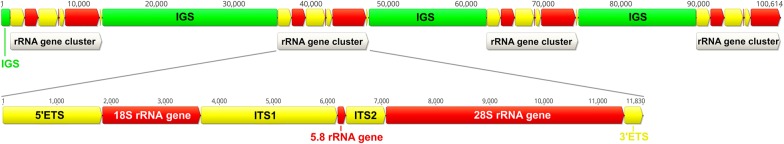
Chicken rDNA repeat structure. Structure of the WAG137G4_utg0 contig obtained by PacBio sequencing of the WAG137G04 chicken BAC clone. The positions of three complete and one incomplete rRNA gene clusters together with three intercalary IGS are indicated. An enlarged diagram of the rRNA gene cluster is shown separately

The WAG137G04 BAC clone was sequenced using PacBio RSII and a single contig (WAG137G4_utg0 contig) was obtained, which contained three full rDNA gene clusters and one incomplete cluster that lacked the 3′ end of the 28S rRNA gene and the 3′ETS (Fig. 1) and (see Additional file 4). The lengths of the full rRNA gene clusters were 11,871, 11,830 and 11,855 bp, respectively, and were all homologous to the described previously rRNA gene cluster of domestic chicken (NCBI Nucleotide: KT445934.2; [22]).

We performed pairwise alignment of these three rRNA gene clusters and detected 33 variable nucleotide positions (see Additional file 5: Table S3), including 13 SNPs and 20 InDel. The 5′ETS region contained the largest number of polymorphic loci (12 of 33), no variant was found in the 5.8S rRNA gene, only two in the 18S and 3′ETS, and a few in the 28S rRNA gene and in both ITS.

### Structure of the intergenic spacer

**Fig. 2:**
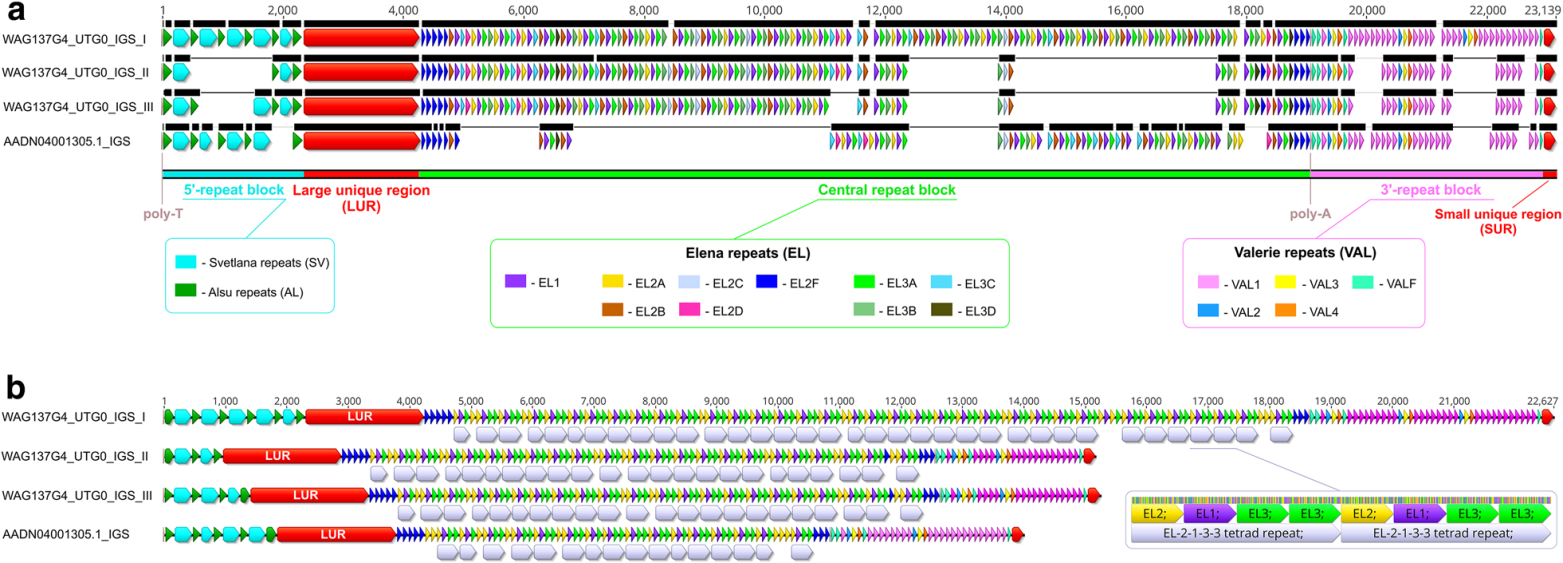
Chicken IGS length variants. Structure of three IGS from the WAG137G04 BAC clone (IGS_I, IGS_II, IGS_III) and an IGS present in the AADN04001305.1 contig of *Gallus_gallus*-5.0. **a** Detailed comparative figures of IGS structural elements distribution. Repeat deficiency regions are designated with fine black lines. **b** Contracted IGS figures, central block repeats are organized into tetrads

In the WAG137G4_utg0 contig, we identified three fragments among the three rRNA gene clusters at positions 13,051–35,677 bp, 47,508–62,677 bp, and 74,533–89,733 bp that were specified as IGS of different lengths (Fig. 2). In addition, a 1179-bp sequence of an incomplete IGS was located upstream of the first rRNA gene cluster. According to the dot-plot analysis data, each IGS contained three internal arrays (blocks) of tandemly repeated sequences and relatively large (1937–1941 bp) and small (190–191 bp) unique regions that are conserved among the IGS (see Additional file 6: Figure S2). In the three IGS analyzed here: the lengths of the first block of repeats (5′ block) ranged from 950 to 2290 bp; the lengths of the second repeat block (central block) ranged from 9297 to 14,414 bp, which represented the main source of the differences between chicken IGS; and the third repeat block (3′ block) was 2400 to 3766 bp long. The structure of these three IGS was very similar to that of the 14,002-bp IGS in the AADN04001305.1 red junglefowl contig (see Additional file 6: Figure S2). Below, we provide a detailed description of the components of chicken IGS based on the four sequences investigated here.

### Annotation of the IGS components

#### Tandem repeats at the IGS 5′end

**Fig. 3:**
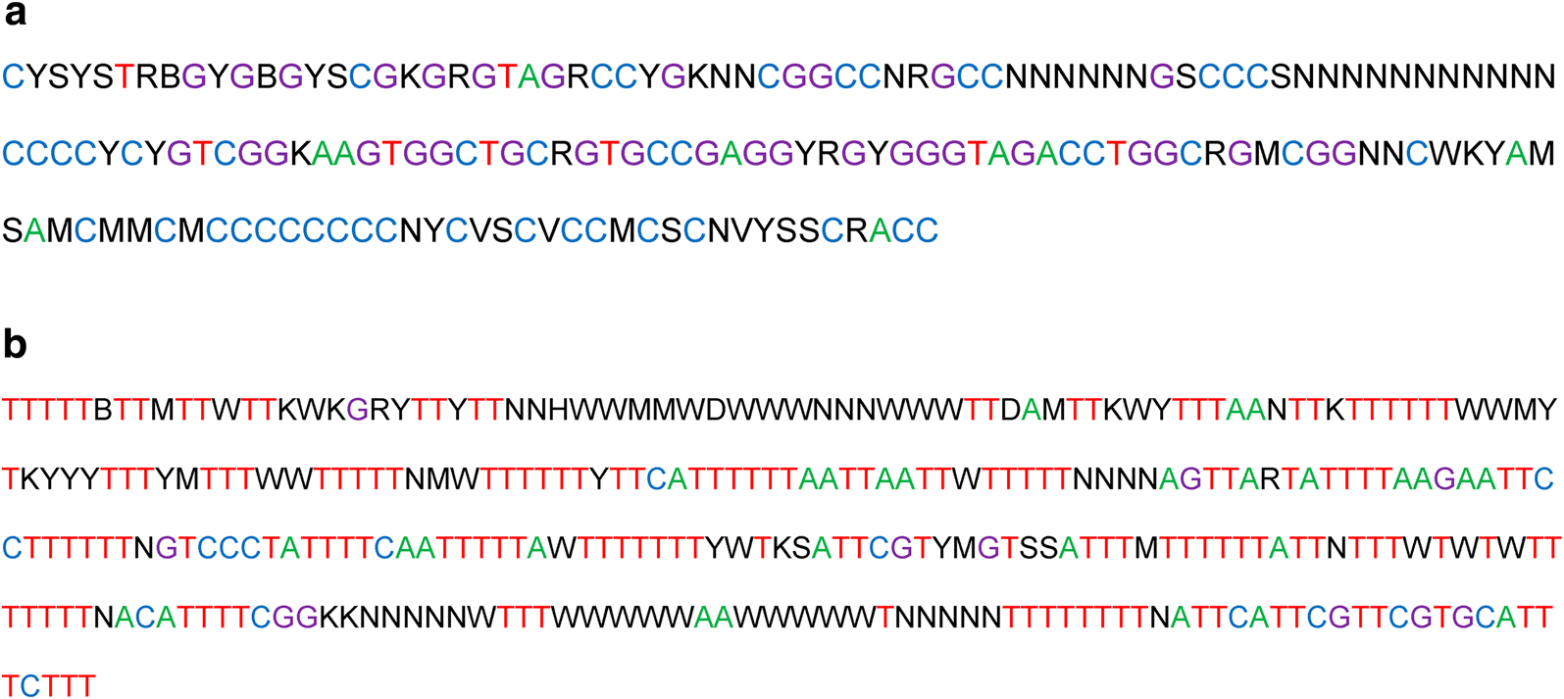
Tandem repeats at the chicken IGS 5′ end. **a** *Svetlana* (*SV*) repeat unit; **b** *Alsu* (*AL*) repeat unit. Both repeats are consensus sequences

The 5′ repeat block is separated from the 3′ETS by a poly-T (9–21 bp) track and consists of two types of phylogenetically related repetitive sequences. The GC-rich repeats have an elementary unit of about 150 bp (hereinafter referred to as *Svetlana* repeats, or *SV*) (Fig. 3a and Table 1) (see Additional file 7). The AT-rich repeats with a 200- and, more often, 300-bp repeat unit (hereinafter referred to as *Alsu* repeats, or *AL*) have multiple oligoT tracks (Fig. 3b and Table 1) (see Additional file 8). Both *SV* and *AL* repeats alternate with each other according to the (*SV*–*AL*)n scheme, which ends with an additional *SV* repeat unit at the end (Fig. 2). The last *SV* repeat unit is preceded by only the shorter (200 bp) *AL* repeat.

**Table 1:** Tandem repeats in chicken IGS

IGS repeat block	Repeat name	Repetitive unit size (bp)	(C+G) content (mean %)	Nucleotide diversity in the repeat sequences in WAG137G4_utg0 contig (%)
5′ repeat block	*Svetlana (SV)*	137–158	78	20
	*Alsu (AL)*	209–303	15	18
Central repeat block	*Elena (EL)*			
	*EL*1	94	75	4
	*EL*2A	93	72	3
	*EL*2B	93	71	6
	*EL*2C	92	72	0
	*EL*2D	92	71	3
	*EL*2F	89/93	72	17
	*EL*3A	93	71	3.5
	*EL*3B	92	69	5
	*EL*3C	93	69	3
	*EL*3D	93	67	7
3′ repeat block	*Valerie (VAL)*			
	*VAL*1	85–95	77	15
	*VAL*2	83	68	0
	*VAL*3	92	80	0
	*VAL*4	82/90	79	10
	*VAL*F	72–92	69	33

#### Large unique region

A unique region of about 1900 bp referred to as LUR (large unique region) is situated downstream of the 5′ repeat block (Fig. 2). The similarity between the 5′ and 3′ ends of the LUR and the adjacent repeats suggests a common origin but with a loss of sequence homology. The central part of the LUR contains regions of low complexity, such as T-rich, C-rich, G-rich regions, as well as several simple repeats: (CT)n, (AGGCG)n, (CCG)n, (CCA)n, etc.

#### IGS central tandem repeat block

**Fig. 4:**
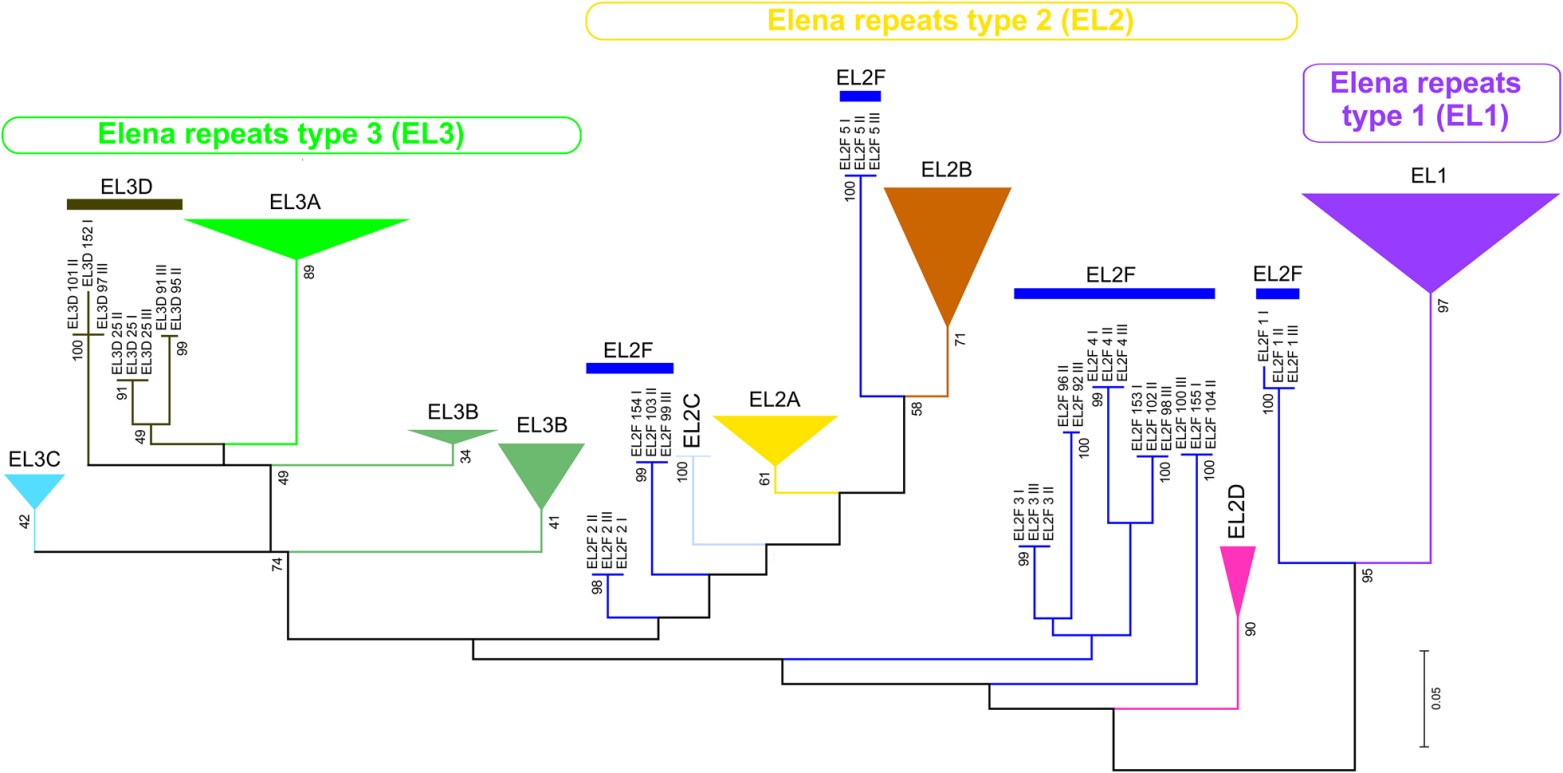
*Elena* repeats in chicken IGS. Relationships between repeats in the *Elena* (*EL*) group. The figure was plotted using the maximum likelihood method. The numbers following the repeat names indicate the repeat ordinal position in the IGS, and the numbers following after a space—the IGS ordinal position in WAG137G4_utg0 contig. An expanded figure is attached in Additional file 4

**Fig. 5:**
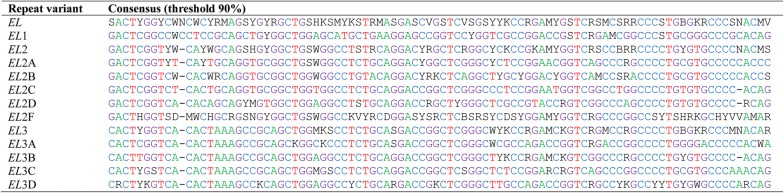
Consensus sequences of *Elena* (*EL*) repeat variants. *Elena (EL)* repeat variants: alignment of the sequences

The central repeat block is the largest and most structurally complex sequence in the chicken IGS. It is composed of short repeats of 92 to 94 bp with a (C+G) content varying between 65 and 76% (Table 1 and see Additional file 9). All these repeats represent one phylogenetically related group (hereinafter referred to as *Elena* repeats, or *EL*) (Fig. 4). *Elena* repeats are degenerated but their lengths are stable (Fig. 5).

Reconstruction of the phylogeny of the *Elena* repeats by the maximum likelihood method showed that they fell into three clades *EL*1, *EL*2, and *EL*3 (Fig. 4 and see Additional file 10: Figure S3). *EL*1 and *EL*3 are monophyletic clades, whereas *EL*2 is a polyphyletic clade. *EL*1 comprises degenerated repeats of 94 bp with 73 to 76% of GC pairs (Fig. 5). Based on their diversity, the *EL*3 repeats (92–93 bp) can be classified into *EL*3A, *EL*3B, *EL*3C, and *EL*3D subgroups. *EL*3 is the most common variant of the *Elena* repeats (Table 1).

The *EL*2 clade comprises a few monophyletic evolutionary lines (*EL*2A, *EL*2B, *EL*2C, and *EL*2D) that represent the variants of similar repeats (Fig. 4 and Table 1). Some of the other EL2 lines combine into the *EL*2F artificial group. Interestingly, each of the *EL*2F lines consists of three identical repeats that belong to three different IGS from the WAG137G4_utg0 contig. These are the first five repeats and the last three or four repeats at each end of the central repeat block. Each repetitive sequence has its own unique SNP profile, which is conserved between the three analyzed IGS and occurs in each IGS as a single copy at a strict unique position. Therefore, these *EL*2F repeats seem to be important structural components of the chicken IGS. Due to the presence of A-rich motifs at the 3′ end of some *EL*2F repeats, their variability was rather high i.e. on average 17%.

A close examination of the distribution of the patterns of *Elena* repeats in IGS from the WAG137G4_utg0 contig and AADN04001305.1 scaffold reveals their highly organized structure. *EL*2–*EL*1–*EL*3–*EL*3 repeats form tetrads with a total length of 372 to 373 bp, which is the main element of the *Elena* repeat type. Putatively, a deletion of one of the *EL*3 repeats may result in the formation of a triad, although these represent exceptions. In general, the organization of the *Elena* repeats in all the IGS studied can be described as (*EL*2F)5 + (*EL*2–*EL*1–*EL*3–*EL*3)n + (*EL*2)2 + (*EL*2F)3.

#### Tandem repeats at the IGS 3′ end

**Fig. 6:**
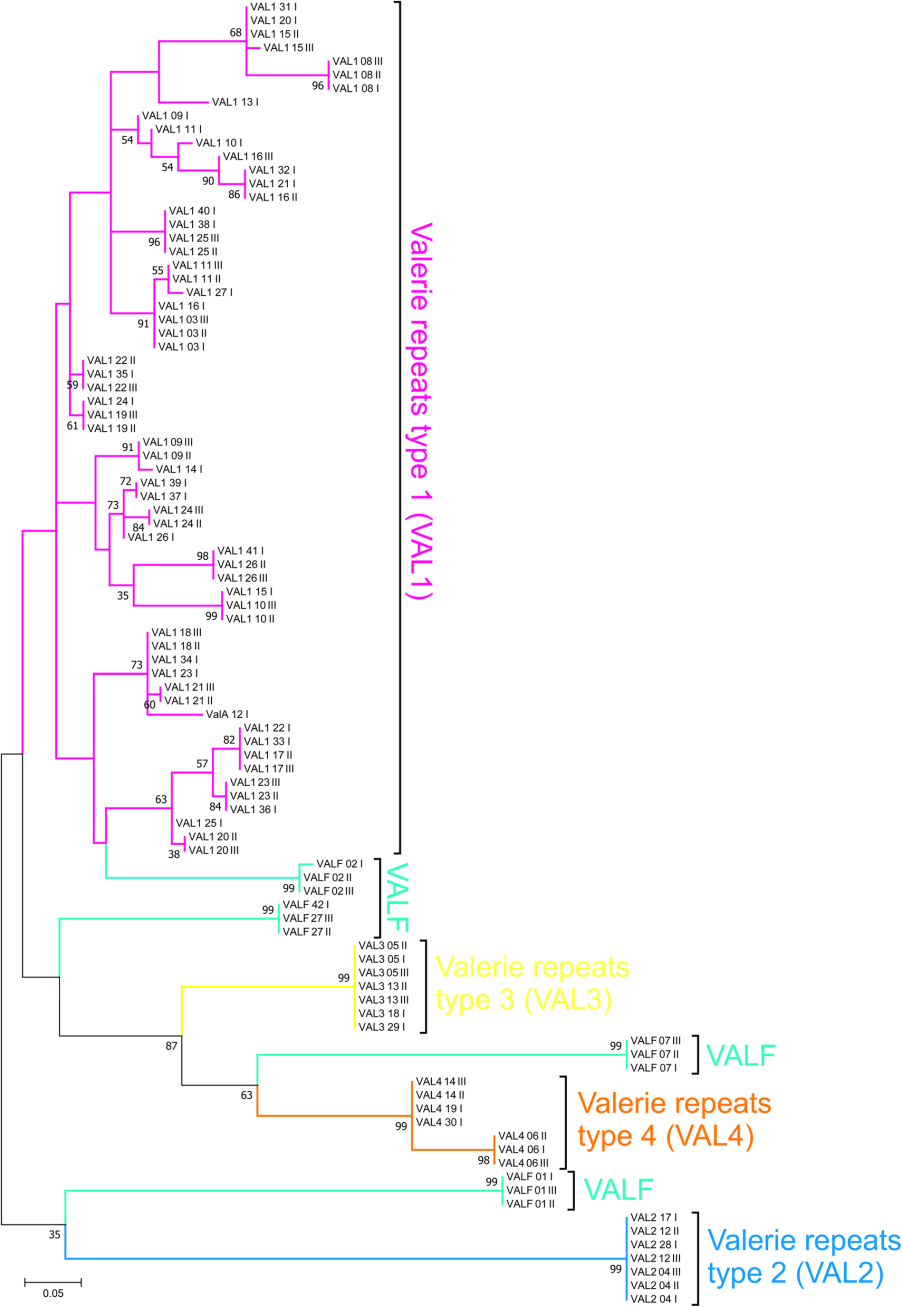
*Valerie* repeats in chicken IGS. Relationships between repeats in the *Valerie* group. The figure was plotted using the maximum likelihood method. The numbers following the repeat names indicate the repeat ordinal position in the IGS, and the numbers following a space—the IGS ordinal position in WAG137G4_utg0 contig

**Fig. 7:**
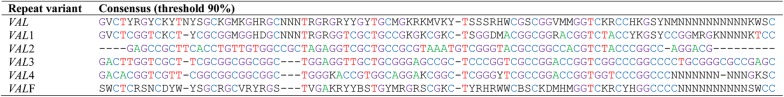
Consensus sequences of *Valerie* (*VAL*) repeat variants. *Valerie (VAL)* repeat variants: alignment of the sequences

The 3′ repeat block is separated from the central repeat block by a (A)_7_ motif. We identified repeats (hereinafter referred to as *Valerie* repeats or *VAL*) that significantly differ from each other (Table 1) within this block. Alignment (see Additional file 11) and phylogeny reconstruction (Fig. 6) showed that the *Valerie* repeats are related. They can be classified into four main clades: *VAL*1, *VAL*2, *VAL*3, and *VAL*4. *VAL*1 repeats are variable and exist in multicopy, whereas only a few copies of *VAL*2, *VAL*3, and *VAL*4 are present. In addition, the *VAL*F clade includes several separate evolutionary lines of *VAL* repeats (Figs. 6, 7).

Similar to the *EL*2F repeats, each *VAL*F repeat variant has a specific set of SNPs and is located at a fixed position in all the chicken IGS studied here (Fig. 2). *VAL*F variants are the first, second and the seventh repeats at the beginning of the 3′ repeat block in both the WAG137G4_utg0 contig and the AADN04001305.1 scaffold sequences. The very last copy in the 3′ repeat block is also a *VAL*F repeat. Low-copy *VAL*2–*VAL*3–*VAL*4 repeats appear to form multiple triads. The number of *VAL*1 copies can vary. In general, the profile of the 3′ repeat block can be represented as (*VAL*F)2 + *VAL*1 + (*VAL*2–*VAL*3–*VAL*4) + *VAL*F + (*VAL*1)n + ((*VAL*2–*VAL*3–*VAL*4) + (*VAL*1)n)n + *VAL*F.

#### Small unique region

The small unique region of 190 to 191 bp is situated at the 3′ end of the chicken IGS, just before the 5′ETS of the following rRNA cluster (Fig. 2). For all four IGS studied here, no substitutions were detected in this region. The (C+G) content is lower than in the IGS tandem repeat arrays (58% vs 65 to 81%). According to the BLAST results, the chicken RNApol I promoter is located in this region (NCBI Nucleotide: DQ112354.1 [21]).

### CpG distribution

**Fig. 8:**
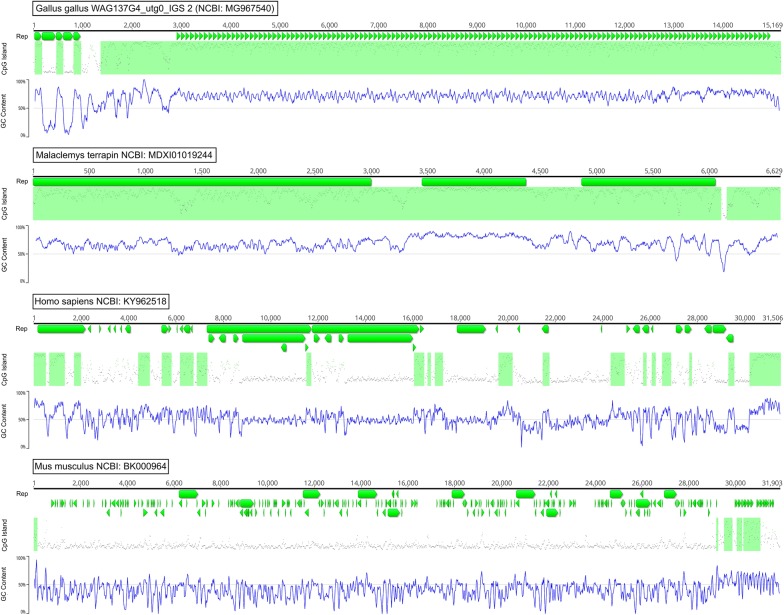
GC content in Sauropsida and Mammalia IGS. (C+G) content and putative CpG island distribution in the IGS of chicken *Gallus gallus*, terrapin *Malaclemys terrapin*, human *Homo sapiens*, and mouse *Mus musculus*. Regions containing repeats are designated with horizontal green blocks (Rep); putative CpG islands—with light-green boxes (CpG Island); GC pairs distribution is shown in the graphs (GC content)

We analyzed the distribution of CpG islands in the chicken IGS sequence studied here (NCBI Nucleotide: MG967540), and in IGS of the following vertebrates: *Homo sapiens* (NCBI Nucleotide: KY962518), *Malaclemys terrapin* (NCBI Nucleotide: MDXI01019244.1), *Macaca mulatta* (NCBI Nucleotide: KX061890); *Mus musculus* (NCBI Nucleotide: BK000964); *Xenopus laevis* (NCBI Nucleotide: AF110804); *Lissotriton vulgaris* (NCBI Nucleotide: X98876); *Perca flavescens* (NCBI Nucleotide: EU325541); *Cyprinus carpio* (NCBI Nucleotide: AF133089); *Acipenser fulvescens* (NCBI Nucleotide: FJ688028). This comparison shows that the chicken and terrapin IGS tandem repeat sequences are very GC-rich and enriched with CpG islands, whereas these features do not seem typical of the other vertebrate IGS sequences studied (Fig. 8 and see Additional file 12: Figure S4).

### IGS transcription

**Fig. 9:**
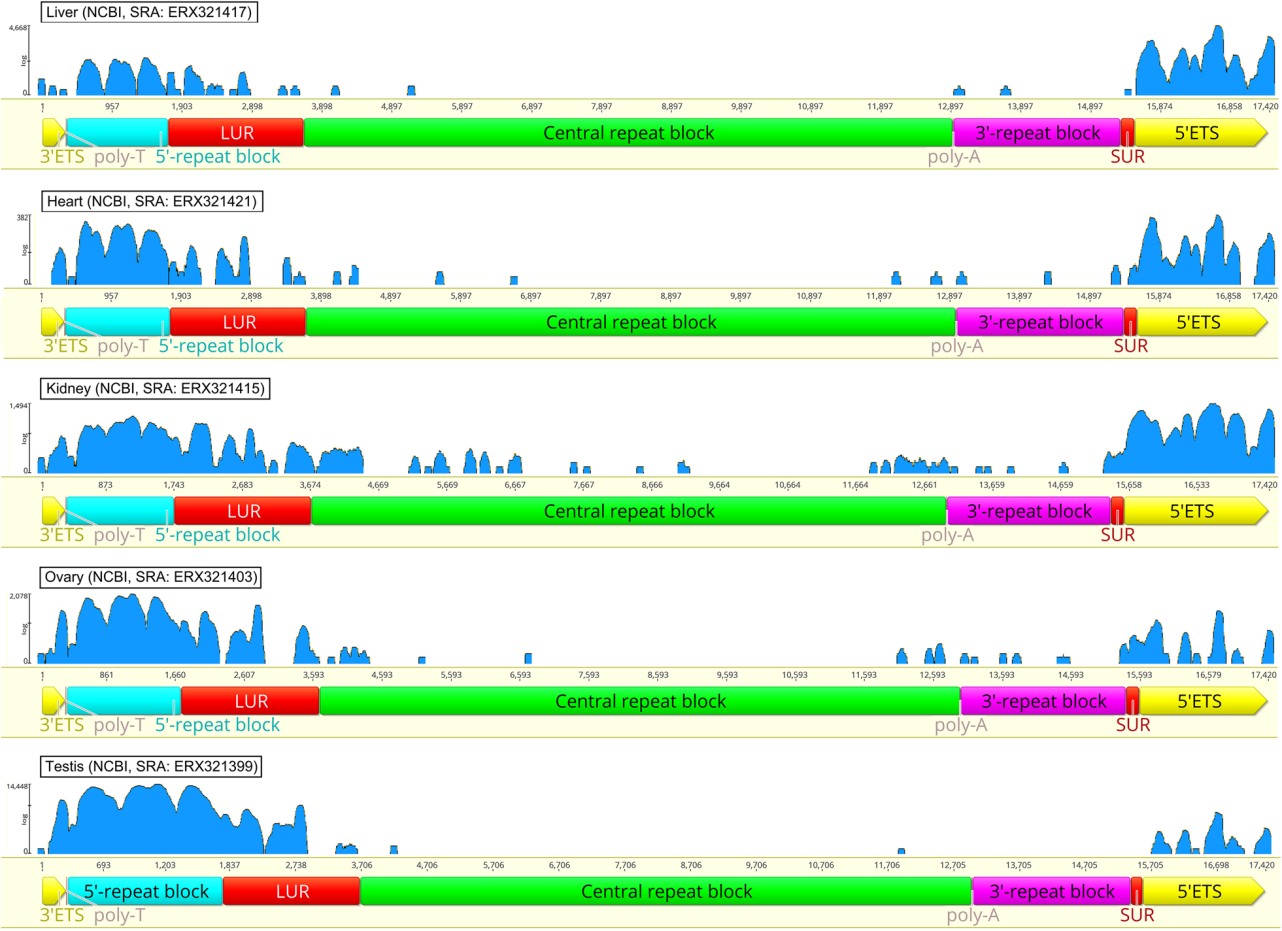
IGS transcription. Analysis of the IGS transcription in different chicken organs. The vertical axis represents read counts aligned to each nucleotide position

The alignment of transcriptome reads from different chicken tissues against the most complete rDNA sequence from the WAG137G4_utg0 contig shows that the 5′ repeat block is transcribed along with the rRNA gene cluster for all tissues. In addition, both the large and small unique regions are transcribed either partly or completely (Fig. 9). Several reads align against some sites of the *Elena* and *Valerie* repeats. However, most of the central region and the *Valerie* repeat region of the IGS do not show any sign of transcription.

## Discussion

In 2016, we published a complete description of a chicken rRNA gene cluster, which consists of the successive 5′ETS, 18S rRNA gene, ITS1, 5.8S rRNA gene, ITS2, 28S rRNA gene, and 3′ETS sequences [22]. In the present work, the sequence of the intergenic spacer (IGS) between the rRNA gene clusters in chicken NOR was analyzed.

A chicken BAC clone, which hybridizes in situ to the NOR on GGA16 [17], was sequenced using the PacBio technique. We found that the insertion in this BAC contained three complete and one incomplete rDNA repeat units with three complete IGS sequences between them. We compared the structure of three complete rDNA repeats from the same BAC clone with a non-annotated complete rDNA sequence identified in a red junglefowl contig AADN04001305.1 from the Gallus_gallus-5.0 assembly, each of these originating from two different chicken breeds. The only crucial differences between these sequences were in the IGS, and not in the rRNA gene clusters. All four IGS contain tandemly repeated sequences organized in complex arrays. A fundamental similarity in structure and sequential location of the key repeats was detected among the four IGS analyzed: we observed no signs of mixing or shifting of nucleotides or clusters of repeated nucleotides and no loss of the high-level organization in any of the four IGS compared. When aligned against each other, the IGS feature a notable difference in lengths which range from 22,627 to 15,170 bp in the same NOR and is equal to 14,002 bp in the other individual. This variability in chicken IGS length is the main cause of the length variation of the rDNA repeat unit that ranges from 11 to 50 kb as was earlier described by Delany and coauthors [19, 20], who performed genome DNA restriction analysis on rDNA repeat sequences and IGS size ranging in several chicken lines and breeds. We examined in detail the molecular pattern of the IGS length variations, which appear to be due to the absence of individual IGS inner repeats and/or of larger blocks of entire repeats. Similar to the variability in rDNA repeat number, the mechanism that underlies the IGS inner repeat number instability can be unequal sister-chromatid recombination or slippage during DNA replication [27, 28]. According to Delany and Krupkin [20], the rDNA repeat number ranges from 269 to 378 per diploid genome in chicken, depending on the genetic line examined. It should be noted that they found larger ribosomal gene copy numbers and smaller rDNA repeat unit sizes in broiler populations compared to egg laying breeds.

In the best-known vertebrate to date, namely *Homo sapiens*, IGS comprise several functional elements such as pre-rRNA promoters, several *Sal* box terminator sites, two sites for non-coding RNA associated with stress response, a cdc27 pseudogene, and putative c-Myc and p53 binding sites, most of these being conserved at least among ape species [7]. In chicken, we were unable to identify any known functional sites within the IGS inner repeats and any known motifs similar to the repeated 10–18 bp *Sal* boxes that have been shown to be a termination signal sequence in human and mouse IGS [29]. One of the *Elena* repeats (*EL2A*) showed 100% similarity to a 24-bp chicken sequence of a microRNA that is deposited in GenBank (NCBI Nucleotide: AM691130.1). Both *EL2D* and *EL2F* were similar to a repeated sequence that is related to antiviral activity (NCBI Nucleotide: AB124589, AB124590.1). To date, no function is known for the majority of the repeats in chicken IGS.

Our alignment of transcriptome reads from different tissues (Chickspress project: http://geneatlas.arl.arizona.edu/) against IGS sequences indicates that, in chicken, the pre-rRNA transcription starts in the small unique region (SUR) followed by the 5′ETS sequence. These data match well the data reported by Massin et al. [21]. These authors sequenced a 1262-bp fragment of chicken IGS and identified an rDNA promoter immediately followed by the start point of transcription. The fact that the sequence of this promoter completely matches to a sequence located in the SUR and the transcriptome alignment data support the existence of a single promoter sequence in chicken IGS, which is the pre-rRNA transcription promoter.

In mouse, Xenopus, and yeast, the rRNA gene cluster transcription termination was shown to end in a T-enriched sequence downstream of the 3′ETS sequence, which is a region that contained no repeats [30]. Chicken rRNA gene cluster transcription appears to continue into the 5′ repeat block of the IGS, in which C-rich and T-rich repeats alternate, and gradually ends in the LUR sequence. Thus, in chicken rDNA, the sites of both the pre-RNA gene cluster transcription termination and the next cluster transcription initiation are situated within the unique sequences that are separated by a series of *EL* and *VAL* repeats. The central IGS region occupied by the repeats of the *EL* group and *VAL* repeats appears to be non-transcribed. As a rule, only a few transcriptome reads are aligned with the central region. Presumably, they represent a nonspecific reaction, since no functional sites were found in this region. An obvious exception is the NOR in the kidney cells, where the reads were more abundantly aligned against *EL* and *VAL* repeat areas. To date, the regulation or the meaning of this phenomenon are unknown. Based on our results, the chicken IGS structure seems to be quite different from its counterpart in human rDNA.

We compared the organization of IGS in two representatives of the Sauropsida group, i.e. chicken (*G. gallus*) and terrapin (*M. terrapin)*. A complete terrapin ribosomal repeat sequence was found among the non-annotated data of the whole-genome shotgun sequencing project (NCBI Assembly: MDXI00000000.1). Unlike IGS of mammals, amphibians or fish, IGS of chicken and terrapin are GC-enriched and contain many putative CpG islands. Besides, they both include very long conservative GC rich tandem repeats and lack the inverted sequences that can form hairpins. These data seem to put the investigated Sauropsida representatives at odds with other vertebrates and should be taken into account when studying the evolution of the Sauropsida group.

In spite of the fast development of genome sequencing techniques, chromosomal NORs are still quite difficult to sequence and assemble. Regarding *G. gallus*, even in the last assembly version (GRCg6a) that includes PacBio and Nanopore data, the NOR bearing chromosome (GGA16) region contains only one complete and two reduced rRNA gene clusters (NCBI Nucleotide: NC_006103.5) and has no IGS sequence. Based on the chicken IGS structure presented here and the ribosomal gene cluster sequence that was determined and published earlier [22], we are confident that a complete chicken rDNA repeat unit is now assembled in terms of molecular DNA organization.

## Conclusions

Our findings fill a knowledge gap about the ribosomal repeat nucleotide sequence in the first representative of birds—*Gallus gallus.* The data obtained on the nucleotide sequence of the chicken IGS and its features combined with our earlier analysis of the chicken ribosomal gene cluster [22], as well as data from Delany et al. [19, 20] on the differences in the number and size of chicken rDNA repeats, allow to characterize the complete organization of chicken NOR rDNA. Remarkably, chicken IGS features a high (C+G) content, a complex repeat sequence organization, no known regulatory sites and only a single promoter sequence. The data presented in this paper allow us to suggest the existence of IGS heterogeneity in a single array of rDNA repeats but this deserves serious consideration. The comparison between chicken and turtle IGS sequences showed that they are very similar to each other and significantly different from IGS in rRNA genes of mammals, amphibians, and fish. This emphasizes the evolutionary separation of the Sauropsida group from representatives of both the lower and higher organized vertebrate taxa.

## Supplementary information

**Additional file 1: Figure S1.** Base per base depth of PacBio sequencing coverage along WAG137G4_utg0 contig (window size = 200 bp).

**Additional file 2: Table S1.** Identification of the best evolutionary model for the description of sequence evolution in *Elena* (*EL*) repeats.

**Additional file 3: Table S2.** Identification of the best evolutionary model for the description of sequence evolution in *Valerie* (*VAL*) repeats.

**Additional file 4.** WAG137G4_utg0 contig nucleotide sequence.

**Additional file 5: Table S3.** Sequence variants of three chicken rRNA clusters from WAG137G04 BAC clone with coordinates, numbers, types, and variant frequencies per kilobases.

**Additional file 6: Figure S2.** Self-similarity dot plot analysis of chicken IGS sequences. (a-c) IGS sequences from White Leghorn WAG137G04 BAC clone: a—IGS_1, b—IGS_2, c—IGS_3; (d) IGS sequence from AADN04001305.1 red junglefowl contig. Three internal blocks of tandemly repeated sequences are marked as the 5′ repeat block, the central repeat block, and the 3′ repeat block.

**Additional file 7.** *Svetlana* (*SV*) repeat alignment. The numbers following the respective repeat names indicate the repeat ordinal position in the IGS; the numbers following after a space—the IGS ordinal position in WAG137G4_utg0 contig. I, II, and III—rDNA unit number in the contig.

**Additional file 8.** *Alsu* (*AL*) repeat alignment. The numbers following the respective repeat names indicate the repeat ordinal position in the IGS; the numbers following after a space—the IGS ordinal position in WAG137G4_utg0 contig. I, II, and III—rDNA unit number in the contig.

**Additional file 9.** *Elena* (*EL*) repeat alignment. The numbers following the respective repeat names indicate the repeat ordinal position in the IGS; the numbers following after a space (I, II, III)—the IGS ordinal position in WAG137G4_utg0 contig.

**Additional file 10: Figure S3.** Relationships between repeats in *Elena* (*EL*) group (expanded). The figure was plotted using the maximum likelihood method. The numbers following the repeat names indicate the repeat ordinal position in the IGS; the numbers following after a space—the IGS ordinal position in WAG137G4_utg0 contig.

**Additional file 11.** *Valerie* (*VAL*) repeat alignment. The numbers following repeat names indicate the repeat ordinal position in the IGS; the numbers following after a space (I, II, III)—the IGS ordinal position in WAG137G4_utg0 contig.

**Additional file 12: Figure S4.** (C+G) content and putative CpG island distribution in IGS of mammals (rhesus macaque *Macaca mulatta,* NCBI Nucleotide: KX061890), amphibia (common newt *Lissotriton vulgaris,* NCBI Nucleotide: X98876; African clawed frog *Xenopus laevis,* NCBI Nucleotide: AF110804), and fish (European carp *Cyprinus carpio,* NCBI Nucleotide: AF133089; yellow perch *Perca flavescens,* NCBI Nucleotide: EU325541; lake sturgeon *Acipenser fulvescens,* NCBI Nucleotide: FJ688028). Repeat regions are designated with horizontal green blocks (Rep); putative CpG islands—with light green boxes (CpG Island); GC pair distribution is shown in the graphs (GC content).

## Appendix

The raw sequences of the WAG137G04 BAC clone generated in this study have been uploaded to the NCBI SRA archive with project ID PRJNA577229, the reference sequence of the chicken rDNA repetitive unit was uploaded to GenBank under the accession ID MG967540.
