# Supplementary figures and images for: Structure of the intergenic spacers in chicken ribosomal DNA
Source: Genet Sel Evol. 2019 Oct 26;51:59. doi: 10.1186/s12711-019-0501-7 (PMC6815422; doi:10.1186/s12711-019-0501-7)

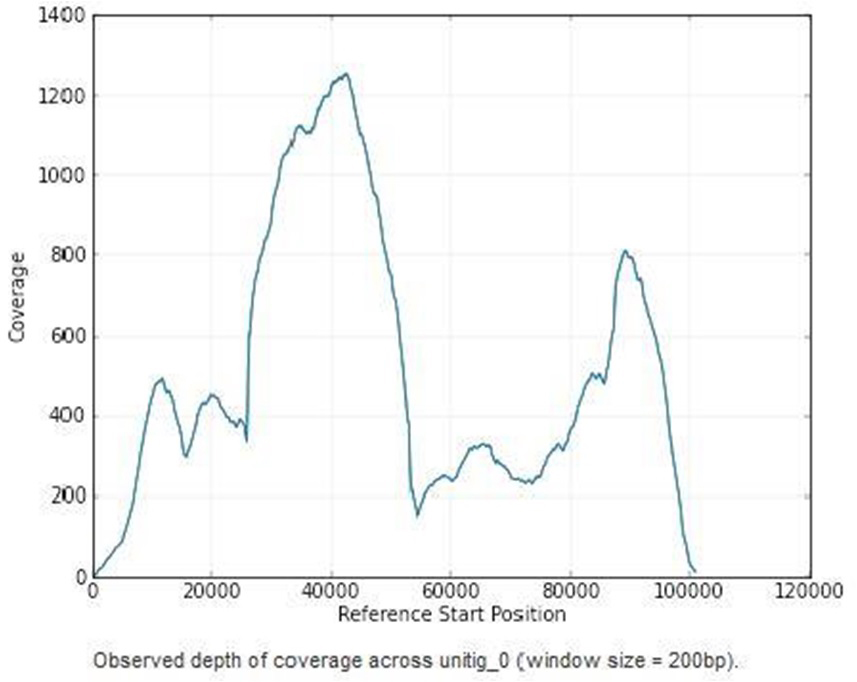

Supplement: Supplementary file 1 — Additional file 1: Figure S1. Base per base depth of PacBio sequencing coverage along WAG137G4_utg0 contig (window size = 200 bp). [file 12711_2019_501_MOESM1_ESM.tif]

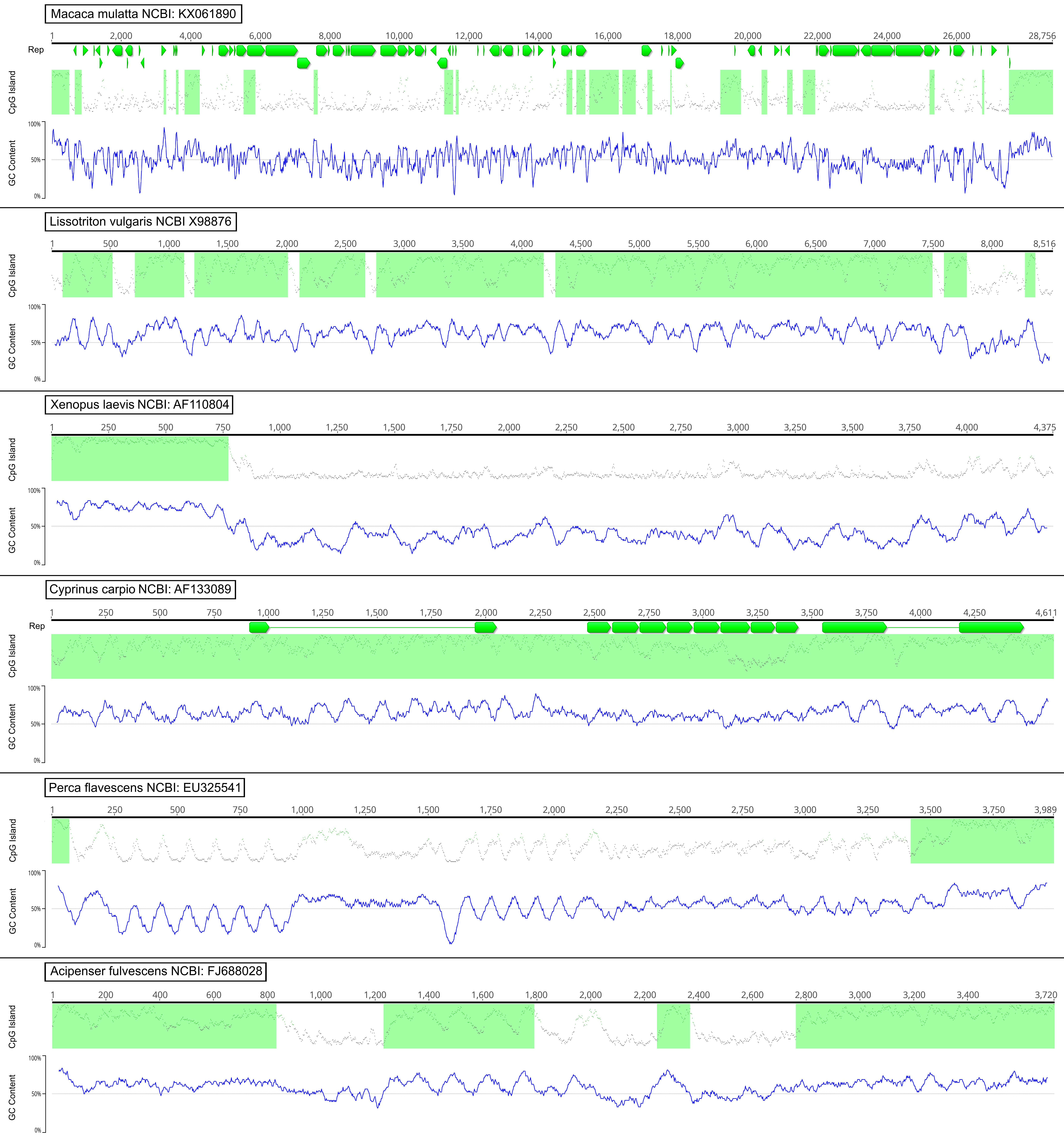

Supplement: Supplementary file 12 — Additional file 12: Figure S4. (C+G) content and putative CpG island distribution in IGS of mammals (rhesus macaque Macaca mulatta, NCBI Nucleotide: KX061890), amphibia (common newt Lissotriton vulgaris, NCBI Nucleotide: X98876; African clawed frog Xenopus laevis, NCBI Nucleotide: AF110804), and fish (European carp Cyprinus carpio, NCBI Nucleotide: AF133089; yellow perch Perca flavescens, NCBI Nucleotide: EU325541; lake sturgeon Acipenser fulvescens, NCBI Nucleotide: FJ688028). Repeat regions are designated with horizontal green blocks (Rep); putative CpG islands—with light green boxes (CpG Island); GC pair distribution is shown in the graphs (GC content). [file 12711_2019_501_MOESM12_ESM.tif]
