# Supplementary material for: Structure of the intergenic spacers in chicken ribosomal DNA
Source: Genet Sel Evol. 2019 Oct 26;51:59. doi: 10.1186/s12711-019-0501-7 (PMC6815422; doi:10.1186/s12711-019-0501-7)
Supplement: Supplementary file 4 — Additional file 4. WAG137G4_utg0 contig nucleotide sequence. [file 12711_2019_501_MOESM4_ESM.docx]

Nucleotide sequence of the contig WAG137G4_UTG0

– *Intergenic spacer* (*IGS*)

– *5' external transcribed spacer* (*5’ETS*)

– *18S rRNA gene*

– *Internal transcribed spacer 1* (*ITS1*)

– *5.8S rRNA gene*

– *Internal transcribed spacer 2* (*ITS2*)

– *28S rRNA gene*

– *3' external transcribed spacer* (*3’ETS*)

GCCTCGGTCGCTCCGCGGAGGAGGCTAGGGGTCGCTGCCGGGGCGTCTCGGAAACGGCGGGACGGTCTACCTTGCCCCGGCAGGCTTCGTCCGGCTCGGTCGCTCCGCGGCGGCGGCTAGAGGTCGCTGCCGTGTCGGCTCGGAAACGGCGGAACGGTCTACCCGGCCCCGGCAGGCTGCGTCCGGCTCGGTCGCTCCGCGGCGGCGGCTAGAGGTCGCTGCCGGGGCGGCTGGGAAACGGCGGGACGGTCTACCCGGCCCCGGCGGTCCCCGTCCGACTTGGTCGCTCCGCGGCGGCGGCTAGAGGTCGCTGCCGGGGCGTCTCGGAAACGGCGGGACGGTCTACCTTGCCCCGGCAGGCCTCGTCCGGCTCGGTCGCTCCGCGGCGGCGGCTAGGGGTCGCTGCCGTGTCGGCTCGGAAACGGCGGAACGGTCTACCCGGCCCCGGCAGGCTGCGTCCGGCTCGGTCGCTCCGCGGCGGCGGCTAGAGGTCGCTGCCGGGGCGGCTGGGAAACGGCGGGACGGTCTACCCGGCTCCGGCGGGCCCCGTCCGACTTGGTCGCTCCGCGGAGGAGGCTAGGGGTCGCTGCCGGGGCGTCTCGGAAACGGCGGGACGGTCTACCTTGCCCCGGCAGGTTTCGTCCGGCTCGGTCTCTCCGCGGCGGCGGCGGCTAGGGGTCGCTGCCGGGGCGGCTCGGAAACGCCGGCACGGTCTACCTGGCTCCGGCGGTCCCCGTCCGGCTCGGTCGCTCCGCGGCGGCGGCTAGGGGTCGCTGCCGGGGCGGCTGGGGCACGGCGGAACGGTCTACCTGGTCCCGGCGGGCACCGTCCGGCTCGGTCTCTCCGCGGCGGCGGCGGCTAGGGGTCGCTGCCGGGGCGTCTCGGAAACGGCGGAACGGTCTACCCGGGTGCTACCGTCTCGCGCTCTCCGCGGCGGCGGCTAGAGGTCGCTGCCGGGGCGGCTTGCGATCCGCGTCCAGGTCTACCCCGTTTCGGATTGTCTTGGCCGCTCTGGCTGTGGGGGGGGGCGCTACAGCTCCGGAGCTGCCAGAGGCGTCGCTGTAATTTTGTACCTCCAGTTACGTCGAGGTAAACCTCGGCTGCCGTCGGAGCCGCTGCCGGTAGTCGGCGCCTATGGGACTAGAACGTTTTTTTCGGATGCCTTATATGTTCGTCTGTAGGAGCGAGTGAGGACTCGGCTCCGGTAGTGGCGGTGAGCGGGCGCTCGCGAGCAGGGTTGACCGGCCGGCCGCCTAGAGAGGGGATCGGCGGCGGCGGCGGCGGCTTTCTCGGGCATCGGTTCGTTCGATCGGTCCGGTCGCTTCGGTTTGTCCGTCGCTCCTCATCCCGCAGCTCTGTCCTGGGCTAAGGCGGTTTTGCAGGCGAGCAGCGAAAAAAAGCCGGAGAAGGCGAGAGAGAGGCAAGAAGCAAGCCGGCTCCCGCGCCGCCAGGGCGAAGGCGAGAGAGAGAGGGAGACGAGAAGGGCACGGGCCGGTCTGCCGGCACCCGAACGTAGGATGGCCGGGGGCGTCCCCGGCGGGTCCCGCCGCGATGGAAGAGGGGGACCCGGAGGTCGTAGGTCGTGGCGGCGTCGCCTCGTCCTCCTTTCGCACCGCATTCTCACCCGCACGCGGGAGCCCCGGCCGATTCGTGGCGCTCCTCGGGCGCGTCGGGGAGGCTTCCCGGCGGGCCGGCTCTATCCCGCTCCCCGGCTCGTTCGGGGTGGCGTGGGGCGGGCCGGTGTTCAGGCACGGGCGAGCACCTCTCGTCGGACGTTGCCCACGCACACCCACCTGCACGTGCGCGTGCGGTCTTTCCGCCGCGCCTGGGGGAAGGGCTCGCGCCTTCTCCCTCCTTTCTTTCTCCTCCCCCCCACCCCCTTTCTCCCACCGATCGATGAGGCCACTCGGGTCGCGTCGGAGAGGGCCCCCGGCGGGCCGGCGCTCTGCGCTCCCTGTCCCAGGGAAGCCGCGGCGGCGTCCGGTGTTCAGGCACGGGCGGCCTCCTCTCCAGTTCGCTTCCCGTCGTTCGCGAGGTGAGGCGCTCGCCCGCTTGGGCCGAGGGCGGCGGCGGCGGCGGCTTCGGGGCGCGTGGCCTCGCCGTGCCGACTCGTCTGTCCGCCCGCCCTGTCGGTGCCCCAGGGCTCGCCCGACCGAATCCAGCTGTGTGACGGCCGAGCGGCCCCGCGAGCCGCAGGCGTACCTATTTCGTTGTGAGCGAGGCGTCGGCGCTGCCCTCGTTTCGGGGCCCGGCGAGTGCCGGCCGCGAGCAGCAAGCCGGCGGGGTGGCAACCGAGGGAAACCGCGGGGAACCGAGGCGAAGCGAGCAGCAGCAGAAGAAGAAGGAACGAGAAGACAACGGGGGGCTGCGCCCGGCCGAGCGGGCGAGCCCGGAGCAGCGCGGCGCGTCCCGCTCCGGATCCGTCGGGGTGTGGGGGCCGGGGGCGTCCGCCGGCCTCTCCTCCGCCTTCGGGCCGCCGCAGCCCGTGTCGGTTTTCCTGCCGCGTCCCCGCCCGCTGCGGAGCGTGCCGCCCCGGGAAAGGGTCTCCGATCGTGGGGTCGCGCCCGTCTCGAGGTCGCGTTCTCCTCTAGCACGTCCGTCTCCGGCGGCGGGGCTTCGTTTCCCCGTCCGCTTCTCCGCCGGTCCCGGAGGGCGGGTCAGCCCCGGCCGGCCGTGCGGCGCGAGCGCGAGTCCGGCTCCCGCGGGGGGGCCCGGAGCGTGCCGCCGAAAGCAGCTGCGCAGCGGTCCCCGCTCCTTCCCCGCGGGGGGGAGGTCGGCGGGGCCGCCCCGGGGATCGGGCGCGCCTCTCCGTCGTGGTCGGCGAGCGAGCGAGCGAGCGAGGGAACGACGGAGGGCCGCCCGCCCCGCCGAGAGGCGTTCGCCCCGGCGGCCGCCGCCGTCGACCCGGCAAGGGCCAGACGGGAAAGCCGAGCGAGCAGGCGAGAGAGAGAGAGAGGGAAGGAGCGAGAGCGGTCGGCGGCGGGCCGGGCCCGTCGGGTCGTGCCCCGTGGCGCGGCTACCTGGTTGATCCTGCCAGTAGCATATGCTTGTCTCAAAGATTAAGCCATGCATGTCTAAGTACACACGGGCGGTACAGTGAAACTGCGAATGGCTCATTAAATCAGTTATGGTTCCTTTGGTCGCTCCCCTCCCGTTACTTGGATAACTGTGGTAATTCTAGAGCTAATACATGCCGACGAGCGCCGACCTCCGGGGACGCGTGCATTTATCAGACCAAAACCAACCCGGGCTCGCCCGGCGGCTTTGGTGACTCTAGATAACCTCGAGCCGATCGCACGCCCCCGTGGCGGCGACGACCCATTCGAATGTCTGCCCTATCAACTTTCGATGGTACTGTCTGTGCCTACCATGGTGACCACGGGTAACGGGGAATCAGGGTTCGATTCCGGAGAGGGAGCCTGAGAAACGGCTACCACATCCAAGGAAGGCAGCAGGCGCGCAAATTACCCACTCCCGACCCGGGGAGGTAGTGACGAAAAATAACAATACAGGACTCTTTCGAGGCCCTGTAATTGGAATGAGTCCACTTTAAATCCTTTAACGAGGATCCATTGGAGGGCAAGTCTGGTGCCAGCAGCCGCGGTAATTCCAGCTCCAATAGCGTATATTAAAGTTGCTGCAGTTAAAAAGCTCGTAGTTGGATCTTGGGATCGAGCTGGCGGTCCGCCGCGAGGCGAGCTACCGCCTGTCCCAGCCCCTGTCTCTCGGCGCCCCCTCGATGCTCTTAACTGAGTGTCCCGCGGGGCCCGAAGCGTTTACTTTGAAAAAATTAGAGTGTTCAAAGCAGGCTGGCCGCCGGAATACTCCAGCTAGGAATAATGGAATAGGACTCCGGTTCTATTTTGTTGGTTTTCGGAAACGGGGCCATGATTAAGAGGGACGGCCGGGGGCATTCGTATTGTGCCGCTAGAGGTGAAATTCTTGGACCGGCGCAAGACGAACTAAAGCGAAAGCATTTGCCAAGAATGTTTTCATTAATCAAGAACGAAAGTCGGAGGTTCGAAGACGATCAGATACCGTCGTAGTTCCGACCATAAACGATGCCGACTCGCGATCCGGCGGCGTTATTCCCATGACCCGCCGGGCAGCTCCCGGGAAACCCAAGTCTTTGGGTTCCGGGGGGAGTATGGTTGCAAAGCTGAAACTTAAAGGAATTGACGGAAGGGCACCACCAGGAGTGGAGCCTGCGGCTTAATTTGACTCAACACGGGAAACCTCACCCGGCCCGGACACGGACAGGATTGACAGATTGAGAGCTCTTTCTCGATTCCGTGGGTGGTGGTGCATGGCCGTTCTTAGTTGGTGGAGCGATTTGTCTGGTTAATTCCGATAACGAACGAGACTCTGGCATGCTAACTAGTTACGCGACCCCCGAGCGGTCGGCGTCCAACTTCTTAGAGGGACAAGTGGCGTTCAGCCACCCGAGATTGAGCAATAACAGGTCTGTGATGCCCTTAGATGTCCGGGGCTGCACGCGCGCTACACTGACTGGCTCAGCTTGTGTCTACCCTACGCCGGCAGGCGCGGGTAACCCGTTGAACCCCATTCGTGATGGGGATCGGGGATTGCAATTATTCCCCATGAACGAGGAATTCCCAGTAAGTGCGGGTCATAAGCTCGCGTTGATTAAGTCCCTGCCCTTTGTACACACCGCCCGTCGCTACTACCGATTGGATGGTTTAGTGAGGTCCTCGGATCGGCCCCGGCGGGGTCGGCCACGGCCCTGCCGGAGCGTCGAGAAGACGGTCGAACTTGACTATCTAGAGGAAGTAAAAGTCGTAACAAGGTTTCCGTAGGTGAACCTGCGGAAGGATCATTACCGGGGCCGAGGCCGGGCGTCCGGCCGAGCCGTGGCACGAGCGCGCGCGGGCGCGCAGCCTTCCCTTCCCTTCCCCGAGCCCGCTCCGCGCGGAGCGCGGCTCCTCTCCCCCGGTCGAAACGGGGAAAGAAAAAAAAAACACCGCAAGTCGCTCCGCGCGCCTGCCGGCGAGAGAGAAGGGAGACGAGGGCGCGGAGCGCAGCTCCGGGGGGGAGGCGCGTGTGGGGCGCTCCGGCGCTCCGGCGCGTCTCTCCCCCCCGGCGCCGGTCCGCCGTCGGTCCGCACGCCGCGGGTCCGGTCCGTCCGGTCGCCTCGCCGGCGCGCGCCCGCGCGCGCGCGTCCCGCGGGCCTCGCCCGGGTCGCCGCGCTCCGGAGCGTCCCGCGGCCGAGTCCCGCTCCGACCGCGGGGTCGGGGTCGGGAGGTGGCGGCGGTGCGGAGGGTGGAAGGACGGCTCCCCGCTTCGTCGCTCGGCCGGAAACTCGCCACCGGCCCCCGCCGCTGTCGACGCCGGCACCCCGAGTCCGCTCGGAGGGAAGCCGCGCGGGCGGCCGCGCGCGGGGGAGGCGGCGGGCGGCGGGTCCGAGCGCGGGGCGCGGGAAGTCGGCCGCTTCCCCCGGCCTCACCCCCCACCCCCTTCGCCCGGCCCGTCGCGGGGACGGGGCCGGGTCGCGGGCGGCTGCGGAGCCGGCCGACTCCGGGCGAGCGCCGGAGGGACGCGCGCGCCGCGTACGCGCGGCAGGCGCGAGGTGCCCCGGGCGGCTTCGGTCCCGCGCGGGCGGTCCGAGCCTCGCGGCTCCTCCCGGGTGCAGCTGCCGCCCGGCGCCGGGTTGCCGAGGGAAACCCCGGGCCCCGGGAGGAACGCGAGGTGGTGGCGGCGGACGTCGGGCGCGCCCCCGCGGGCGGACGCTCCCCCGAGGGGCGCCGGGGCCGGCTGGCGGGTGCCGGGTCTCCCCTCGGCGCCCCGTCCCGCCCCGCCGAGCGGGGCGGGCGGGGGAGGCACCCCCGCGGGGCCTTCGGGTCGTTTCCCTCACCCCAGGGCCAGGTACCTAGCGTCCGCGCCTCCGCGCGTCCGGGGGGCGGGGAGGAAGGAGCGCGGCGCCGGTCCCGAGCGGGCCGCGTCGCCCACACCCCCCTCCTCCCCCCGGGCCGCGGAGCCGGGCGGAGGTTTAAAGACTCGGGCGGCCCGCGGCGCGCGCCGCGAGGTCGGGGGCCGGGGGCGGTCTTCTGCCCGCCGGCGGGACGCCGGGATGGAAGAGAGGAGTCCGGGCGGGGCGCGGCGGCGCGCCCCGCCGGCCCTCTCCCTCCCGAGCCCGCCGGCGGCGTCGGCCGTCGCCGCGCCCTCGGTCCTCCGCGGGGCGGGCCCGGGCCGGAGAGGGGGTCATCCCGTCCCCCCTCTCCGCGGCCTCGGTCTCGGGCGGAGAGCTCGGCGCGCGCGCGGGCGCGCGCTCGCTCCGGCCGGCCTCGCCCGCGTACGGAGCGGGCCGAGACGCGGGTCTCGGCCCGGCGCCCGCCGCTCCCCGCGGCGGTGCGTTCCGCGGCCTCCCCGCCGCGCGCGGCCGGCGGGACGGCGAGCCGGCCGTCCCGCCCGCGCCAGCCGCGGCGCCGGCGGTTCCGCTCCGCCGGTCCGCCCGGCGTGCGTCCGCACGCCCGGCCTCCTGCCCTCCCTCGGGGCCTCGCCGCCGTTTCCCCCTTCCGTCGCAAGCCGCGTCCTCTCCTTCGTCCCCGCCGCCGTCGCCTCCCACCGCGCTTTCGCCCTCGGCCTCGCCGGCCGCGCCGGTCGTGCGAGCGGGAGGTCCGGCGTGGGGCGTCCCGCAGCCGGTCTCCGCGCGGAGGCGCGGGGAGCGGGCGCCGCTCCCGAATCCGTCCCCGTCCCGCCCGCCGCCGTGCGCGCGTCCGCCGCGGGCGCGCCGCCAGGGCGAGCGAGAGGAGGAGGCGTCGGAGGACGAGGGGCGGGGGAGGAAGGTGAGAGGCGGCGGGGGCGTTTCGGTGCGCGCGTCTCCCGCACGGCGAGGAAGGGGCCGAGGTCGGCGCGGGCGCCGTCGGGCGGTCCGGCGCGGGCGCGGGCCGGCGGCGCCGCCGGCGCGGGCGGGGGCCTGGTCTGCTCCCGTCCCCGTCGGTCGCGGCGGCGGCGGCGGCGGTCCGTCGCGGCAGCGGGGCTTCGGCCGGGGCGGCGCGCGCCGTCCCGCGGGCGTCCGCGGCTCCTCCGCCCGGGCCGGGCCGAGCCGGGCGCCTGGTCCGTCCCCGAAGCGAGACAGGGTCGTTTCCCCAGGTCGGGAGCGAGGGCTCCCCGCCCTTCTCGTTCGGGTCGCGCTTCATTGCCGGCCGGCCGGCCGGCCGGCCGTCGCCGGCTTTTTTTTTCCCTCCCGCATCCGATATTCGTGTGCTCGTACGGTCAGCGGAGGCGACGCTCGTCCGCCCCGCGGTCGCCCCGGCGTCGGGGCTGGCCGCGGGCGCGGGCCGAGCGCCTTCGGGCAAGGCGAGAGAGAACGAGAGCGGTCCCCCCGCGCGCGCGGGGCGGTGCCGAAAGTCAGACAACTCTTAGCGGTGGATCACTCGGCTCGTGCGTCGATGAAGAACGCAGCTAGCTGCGAGAATTAATGTGAATTGCAGGACACATTGATCATCGACACTTCGAACGCACTTGCGGCCCCGGGTTCCTCCCGGGGCTACGCCTGCCTGAGCGTCGCTTGACGGTCAATCGCCGACGGCCGCCGTCCGCGGCGGCCGCGCGGCGCGGCTGGGGCGCCTCGCAGGCCCGCGCGCCCCGCCGGAGGCGGGTCGCGAGGGGGGGGGCCGCCGTCCGTCCGTCCGTCCGCCCGTCGGTCGGTCGGTTCGGGCGCCCGGATTCCCTCCCCCGCACCCCCTCCGAGCGGCGTCGCGCCGCGGGCCTTCGTCCCCCTAAGTGGAGACCCAGGTCGGGGAGCTCGCCGAGCTCCCCGCGCTCCCGGAGCGCCCGCTTTGGCCGAGCTCGTCCCCACGGGGCGGCCGGGCTTTCCGGTCGGTCGCGCGGCGCAGCGCGGCGGGGCCGGACGTTCGTTCGTTCGTTCGTTCGTCCGGCCCCCCGCCCCGGAGGAGCGCACCTCGCCCTCCCCGGCCCCGCGCGCGGCTGCCTGCGGGTCGCGTTACCGGCGGCGGTAACGCGCCGTGCTGCCGCGCGCGTGGCGGTCCGGGTCGGGGCGAGGCTGCCGGCCTCCGGTCGTCCGCCCGTCCGTCCGGCCGAGCCCGGCGCGCGTCCCCGCGGGTCCGTCTCCGGCCACCGTGCGCCGGCGGCGGCGGCGGCGGCGGTGCGAACCGCCGGCGGCGCGCCGGCTCCCCCGTCCGGGCGTTCCTCCCTCGGCAGCGCCGGGAGCAGCCGCTTGGCGTCCGAAGGCGGGTGGCCGGGCGAGCGCGGGCTCGCCCGGGGCCCGGCGTTCGGGCCCCGTTTCCGATCGCGACCTCAGGTCAGACGTGGCGACCCGCTGAATTTAAGCATATTAGTCAGCGGAGGAAAAGAAACTAACGAGGATTCCCTCAGTAACGGCGAGTGAAGAGGGAAGAGCCCAGCGCCGAATCCCCGCCCCGCGGTGGGGCGCGGGAGGTGTGGCGTACGGAAGCCCCCATCCCCGGCGCCGCTCTCGGGGGGCCCAAGTCCTTCTGATCGAGGCCCAGCCCGCGGACGGTGTGAGGCCGGTAGCGGCCCCCCGGCGCGCCGGGCCCGGGGCTTCTCGGAGTCGGGTTGCTTGGGAATGCAGCCCAAAGCGGGTGGTAAACTCCATCTAAGGCTAAATACCGGCACGAGACCGATAGCCAACAAGTACCGTAAGGGAAAGTTGAAAAGAACTTTGAAGAGAGAGTTCAAGAGGGCGTGAAACCGTTAAGAGGTAAACGGGTGGGGTCCGCGCAGTCGGCCCGGAGGATTCAACCCGGCGGGCCAAGGTCGGCCGGCGCGGGCGCCGTCGGATCCCCGCCTCCGCCTCCCCTCCGTCCCTCCCCTTCGCCGGGGCGGGGCGGGCCCAGGGGGGGCGGGCGGGCCGGGGACCGCCGCCCGGCCGGCGTCCGGCCCCCGTCGGGCGCATTTCCTCCGCGGCGGTGCGCCGCGACCGGCTCCGGGACGGCTGGGAAGGGCTGCCGGCGGGCAGGTGGCCCGGCGCCGCGCGAGCGGCCGCCGGGTGTTATAGCCGCCGGGCCCGGATCGTCGCCGAATCCCGGGGCCGAGGGAGAGGACCGCCGCCGCGCCCTCCCCCGGAGGGGGCGGCCCCCCGGAGGGCCCCCCGCGGCCGGACCGGCGTCGGGCCGGCCGCGCCGCGCGCGCGTCCGCGCCGCCGCCGTACGCCGCCGCTCGCTCTCTCTCCGTTCCCCGCCCCGGGTCCGTCCCGGGGCGCGGGGGCGGGGGGGTCGGGTGTCCGGCGCGCGGCTCGGCGCGGCGCCGCGCGTGTGGCGCGCGCCTCCAGCCCGGCGCGGGCGAGGCCGCGGGGGGCGCCGGGGGGGAACCTTCCCCCTTCTGTTCGGGCCGCCTCCGTTCCCGCGGGGGCGGCCCGTTCGGGGGACGGGCCCGCCGGCCCCCGGCGCCGCTGTCCGACCGGGGCGGACTGCGCTCAGTGCGCCCCGACCGCGCGGCGCCGCCGGGCCGGGCTCGGGCCACGCCAGGGCGCCCGGGGTCCGCGGCGACGTCGGCTACCCACCCGACCCGTCTTGAAACACGGACCAAGGAGTCTAGCACGCGCGCGAGTCGGCGGCTCGCGCGAAAGCCCGCGGCGCAATGAAGGTGAGGGCCGGCGCGCGCCGGCTGAGGTGGGATCCCGGGGCGGCAGGCCGGAAGGCCCCGGGCGCACCACCGGCCCGTCTCGCCCGCCTCGCCGGGGAGGTGGAGCATGAGCGCGCGTGCTAGGACCCGAAAGATGGTGAACTATGCCTGGGCAGGGCGAAGCCAGAGGAAACTCTGGTGGAGGTCCGTAGCGGTCCTGACGTGCAAATCGGTCGTCCGACCCGGGTATAGGGGCGAAAGACTAATCGAACCATCTAGTAGCTGGTTCCCTCCGAAGTTTCCCTCAGGATAGCTGGCGCTCGGGGCGGCGGTGCAGTTTTACCCGGTAAAGCGAATGATTAGAGGTCTTGGGGCCGAAACGATCTCAACCTATTCTCAAACTTTCAATGGGTAAGACGCCCGGCTCGCTGGCGTGGAGCCGGGCCGTGGAATGCGAGCGCTCAGTGGGCCACTTTTGGTAAGCAGAACTGGCGCTGCGGGATGAACCGAACGCCGGGTTAAGGCGCCCGATGCCGACGCTCATCAGAGCCCAGAAAAGGTGTTGGTTGATCTAGACAGCAGGACGGTGGCCATGGAAGTCGGAACCCGCTAAGGAGTGTGTAACAACTCACCTGCCGAATCAACTAGCCCTGAAAATGGATGGCGCTGGAGCGTCGGGCCCATACCCGGCCGTCGCCGGCGGTGCGGAGCCGCGGGGGCTACGCCGCGACGAGTAGGAGGGCCGCTGCGGTGCGCCTGGAAGCCTGGGGCGCGGGCCCGGGTGGAGCCGCCGCAGGTGCAGATCTTGGTGGTAGTAGCAACTATTCAAACGAGAGCTTTGAAGGCCGAAGTGGAGCAGGGTTCCATGTGAACAGCAGTTGAACATGGGTCAGTCGGTCCTAAGCGATAGGCGAGCGCCGTTCCGAAGGGACGGGCGATGGCCTCCGTTGCCCTCAGCCGATCGAAAGGGAGTCGGGTTCAGATCCCCGAATCCGGAGCGGCGGAGACAGGCGCCGCGAGGCGCCCAGTGCGGTAACGCAAGCGATCCCGGAGAAGCCGGCGGGAGCCCCGGGGAGAGTTCTCTTTTCTTTGTGAAGGGCCGGGCGCCCTGGAACGGGTTCGCCCCGAGAGAGGGGCCCGCGCCTTGGAAAGCGTCGCGGTTCCGGCGGCGTCCGGTGAGCTCTCGCTGGCCCGTGAAAATCCGGGGGAGAGGGTGTAAATCTCGCGCCGGGCCGTACCCATATCCGCAGCAGGTCTCCAAGGTGAACAGCCTCTGGCATGTTGGACCAATGTAGGTAAGGGAAGTCGGCAAGCCGGATCCGTAACTTCGGGATAAGGATTGGCTCTAAGGGCTGGGTCGGTCGGGCTGGGGCGCGAAGCGGGGCTGGGCGCGCGCCGCGGCTGGACGAGGCGCCGCCCGCCCCCGCCCCCCCTTTCCCCGCTCCCGCTCGCCGGGGCGCCGGGGGGGGGGTCAGCGGGCGGCGCGGCGGCGGCGACTCTGGACGCGCGCCGGGCCCTTCCCGTGGATCGCCCCAGCTGCGGCGGGCGCCGCTCGCCCCCCTCCTTGCCCCTCCGCCCCCCGCTCCCGGCGCCCCTCCCGTCGGCCGTCGTCCCGGCCGCCCCCCGTCCCGAGCGCCCTCCCCGCGAGGCGAGGGCGCGAGGGGCGGCGGCGGCGGCCGCGGGCGCGGCGGCGGCGGGGGGGGCCCGCCGGCGGCGCCGGGCGGGGCGGTCCCGGGCGGGGGGGGTCTCCGGGCCGGCGCCCCGCCTCGGCCGGCGCCTAGCAGCCGGCTTAGAACTGGTGCGGACCAGGGGAATCCGACTGTTTAATTAAAACAAAGCATCGCGAAGGCCCGCGGCGGGTGTTGACGCGATGTGATTTCTGCCCAGTGCTCTGAATGTCAAAGTGAAGAAATTCAATGAAGCGCGGGTAAACGGCGGGAGTAACTATGACTCTCTTAAGGTAGCCAAATGCCTCGTCATCTAATTAGTGACGCGCATGAATGGATGAACGAGATTCCCACTGTCCCTACCTACTCTCCAGCGAAACCACAGCCAAGGGAACGGGCTTGGCGGAATCAGCGGGGAAAGAAGACCCTGTTGAGCTTGACTCTAGTCTGGCGCTGTGAAGAGACATGAGAGGTGTAGAATAAGTGGGAGGCCCCGCGGTCGCGCGACCCGCGCCGCGGCCCGGCCGCCGGTGAAATACCACTACTCTGATCGTTTTTTCACTTACCCGGTGAGGCGGGGGGGCGAGCCCCGAGGGGCTCTCGCTTCTGGCGCCAAGCGCCCGGCGCGCGCCGGGCGCGACCCGCTCCGGGGACAGCGTCAGGTGGGGAGTTTGACTGGGGCGGTACACCTGTCAAAGCGTAACGCAGGTGTCCTAAGGCGAGCTCAGGGAGGCCAGAAACCTCCCGTGGAGCAGAAGGGCAAAAGCTCGCTTGATCTTGATTTTCAGTACGAATACAGACCGTGAAAGCGGGGCCTCACGATCCTTCTGACTTTTTGGGTTTTAAGCAGGAGGTGTCAGAAAAGTTACCACAGGGATAACTGGCTTGTGGCGGCCAAGCGTTCATAGCGACGTCGCTTTTTGATCCTTCGATGTCGGCTCTTCCTATCATTGTGAAGCAGAATTCACCAAGCGTTGGATTGTTCACCCACTAATAGGGAACGTGAGCTGGGTTTAGACCGTCGTGAGACAGGTTAGTTTTACCCTACTGATGATGTGTTGTTGCGCTAGTAATCCTGCTCAGTACGAGAGGAACCGCAGGTTCAGACATTTGGTGTATGTGCTTGGCTGAGGAGCCACTGGAGCGAGGCTACCATCTGTGGGATTATGACTGAACGCCTCTAAGTCAGAATCCCCCCTAAACGTAGCGATACCGCAGCGCCGAGGCGCCTCGGTGGGCTCGCGATAGCCGGCCGCCGCCCCCCTCGGGCGGGCGGTCGGTGCGGAGCGCCGCTCGTGGTCGGGACCGGAGCGCGGACAGATGTGGCGCCGCCTCTCCCCCGCCGCGTACCGCATGTTCGTGGGGAACCCGGTGCTAAATCATTCGTAGACGACCTGATTCTGGGTCGGGGTTTCGTACGTAGCAGAGCAGCTCCCTCGCTGCGATCTATTGAGAGTCAGCCCTCGACACAAGCTTTTGTCGGAGCGCGGAGCGCGCGCGCGCGCGCGCGTGGCGGCGCCCCGGCGCGGGGCCGGGTCCGGCGGGCCAGTCGGTCGGCTCCCCGCGCCGCTCCGTTTGTTCCTGGGTTCGTTCGTTCGTTCGTTCCTTCCTTCCCCGGCCCCGCGCCGGCGCCGGCGCGGGGTTGGAAAGAGGGGGAGAGGGGCGGGGGGCGCGGCCGGCCCCCTTCCCCGTTTCCGTCCCCGCGGCGCGTGCTGTGGGACGGGCTCCCTCCGTTTTACCCGAGCCCGGGGGTTGACCTGGCGGCGGCCGGGCTAGGGGGCGCTCCGCGTCCCCCTTCGGGGGGTTGACCTGTCGGGCGTTTTTTTTTTTATTTTTTTCTCCCTAGGCGGGTCCGGGGGTAGACCTGTCGGCCGGCCGGCCCGGCCCAGCACGCCCCCCCGCCGGCAAGTGGCTGCGGTGCCGAGGTGGCGGGTAGACCTGGCGGCCGGCAGTACGAACCCCCCCCCCCCCCCCCCCCCCCCCCACCCCGTTTTTTTATTTTTGAGGGTTTTTTTCCTTCCTGTTTTTTTTTTTTAATTTTCTTTAAATTGTTTTTTTCCTTTTCTTTTATTTAATTTTTTCTTTTTTTCTTCATTTTTTAATTAATTTTTTTTAGTTAGTATTTTAAGAATTCCTTTTTTGTCCCTATTTTCAATTTTTATTTTTTTTCTTGGATTCGTCAGTCGATTTATTTTTTATTTTTATTTTTTTTTTTACATTTTCGGGGTTTTGATTTAAAAAAAAAAAATTATTTTTTTTTTTATTCATTCGTTCGTGCATTTCTTTCCGTGTGCGTGTGCGCGTGAGTAGGCCCGGCCCGGCCCGGCCCGGCCCGGCCCGGCCCAGCACGCCCCCCCGTCGGTAAGTGGCTGCGGTGCCGAGGCAGTGGGTAGACCTGGCGGCCGGCTTTACGACCACCACCCCCCCCTCGCCATTTTTGTTCTTATTTTTGACTTTTTTAAAAAAAAAAAAATTAAATTGACTTTAATTTTTTTTTAAATTGCCTTTTCCTTTTTTTTTTTAATTTTTTTTTCATTTTTTAATTAATTTTTTTTTTTTAGTTAGTATTTTAAGAATTCCTTTTTTGTCCCTATTTTCAATTTTTAATTTTTTTCTTGGATTCGTCAGTCGATTTATTTTTTATTATTTTTATATTTTTTTTACATTTTCGGTTTTTTATTTTTTTTTAATTTTTTTAATTTTTTTTTTATTCATTCGTTCGTGCATTTCTTTCCGTGTGTGTGCGCGCGTGAGTAGGCCCGGCCCGGCCCGGCCCAGCACGCCCCCCCCCCCCGTCGGGAAGTGGCTGCGGTGCCGAGGCAGTGGGTAGACCTGGCGGCCGGCTTTACGACCACCACCCCCCCCTCGCCGTTTTTGTTCTTATTTTTGACTTTTTTTTTTTCTTCCTTCCTGTTTTTTTTTTATTTTTTTTAAATTGATTTTTTTCCTTTTTTAAAAATTTTTTTCTTTTTTTTTTCATTTTTTAATTAATTTTTTTTAGTTAATATTTTAAGAATTCCTTTTTTGTCCCTACTTTTTATTTTTAATTTTTTTTTATTGGATTCGTCAGTCGATTTATTTTTTATTTTTATTTTTTTTTTTTACATTTTCGGGGTTTTGATTTAAAAAAAAAAAATTATTTTTTTTTTTATTCATTCGTTCGTGCATTTCTTTCCGTGTGCGTGTGCGCGTGAGTAGGCCCGGCCCGGCCCGGCCCGGCCCGGCCCGGCCCAGCACGCCCCCCCGTCGGTAAGTGGCTGCGGTGCCGAGGCAGTGGGTAGACCTGGCGGCCGGCTTTACGACCACCACCCCCCCCCTCGCCATTTTTGTTCTTATTTTTGACTTTTTAAAAAAAAAAAAATTAAATTGACTTTAATTTTTTTTTAAATTGCCTTTTCCTTTTTTTTTTTTAATTTTTTTTTCATTTTTTAATTAATTTTTTTTTTTTTAGTTAGTATTTTAAGAATTCCTTTTTTGTCCCTATTTTCAATTTTTAATTTTTTTCTTGGATTCGTCAGTCGATTTATTTTTTATTATTTTTATATTTTTTTTACATTTTCGGTTTTTTATTTTTTTTTAATTTTTTTAATTTTTTTTTTTATTCATTCGTTCGTGCATTTCTTTCCGTGTGTGTGCGCGCGTGAGTAGGCCCGGCCCGGCCCGGCCCAGCACGCCCCCCCCCCCGTCGGGAAGTGGCTGCGGTGCCGAGGCAGTGGGTAGACCTGGCGGCCGGCTTTACGACCACCACCCCCCCCTCGCCGTTTTTGTTCTTATTTTTGACTTCTTTTTAAATTTTTTTTAAATTGACTTTTTTTTAAATTTTTTTTTAAATTGCCTTTTTCTTTTTTTTTTAATTTTTTTTTCATTTTTTAATTAATTATTTTTTTTTAGTTAATATTTTAAGAATTCCTTTTTTTGTCCCTATTTTCAATTTTTAATTTTTTTTATTCATTCGTTCGTGCATTTCTTTCCGTGTGCGTGCGCGCGTGAGTAGGCCCGGCCCGGCCCAGCCCAGCACGCCCCCCCCCCTCTGTCGGGAAGTGGCTGCAGTGCCGAGGTGGCGGGTAGACCTGGCAGACGGAGCTTCAACACCAACCCCCCCCCCCCCAGCACCCCGCCCTGGCAACCTTTTCTGCATTTTTTTTAATGATTTTTGATTTTTCTTTTCCCGTTTTTTTTTTTTTTAAATTGATTTTTTTTCCCCTATTCCCCCCCCCCCCCCTTTTTTTTTTTTTTTTTAAAAAAAAAAAAAAGGTGTCCCCCCCTTCCGATGGCTCGGCCGGATAGTGACTCGACTTGGCGTCCGGTGCCCGGCACCCCCCCCCCCAATCCCCTGTATCCGCTCCACCTGATCTGCCCCCCGCTCCTTTTTCTCTTTTTCTCCCCCACATTCGTTCCTTCGTTTGTTCGCTCGCTCTTTATTTCTCTCTCTCTCTCTCATCTCTCTCTCACTCTCTCCATCTTTCTTTCTTTCCTTCTTTGCCTGGGTTCCCCCCCCCCCCCTTACTTTCTTCTTAAATACGTCCGTCCATCCGTGCCATTGGTCGTTTGCTCAGTCGTCGCCCGTTTCTTCCTCGTTCTCGGGGGAAAGTCGGACGGGAGAGGGTCACGGGATTGTTATTTATTTATTCCTCCCTCCCCCCCCCCCCCCCCCCCCCCTTTCTCTCGCCGTTCATGCCCGCTCCCCTCCTCCCCCCCACTCGACTCCCGAAGGGCTGGACCCGACGGCTCGGCCGGCCAGTGGCGAGACAGAAGGGTCGGCGCCCCAGCCCCACCCCCCGGATCCGCCCCGCCTGATCGGCCCCCTTTTGTTCGTCTTTCCCCGCCGTTTTTTTGGTTTTGTTTTGTTTTTGTTTTCTGAACGCTGCTTCTCCACACCCCGCCACCCCCCCCCCGCTTTGATTTTTTTTTCCCGTGCCTTTCTTTCCTCCGGCGGGCACGACCTGATGTCTCGACCGGTCCCCGCGCCGCTCACACCCGCACCCCCCCCCCGCGCAACTCCTCCCTGCCCTCCCCCAACCGTGGACCCACCCCCAGCCCGCCACGCGCTTTCTTTCCATTACACTCTTGCTTTCCGTTTTTTTTTTTTTTTTCTACCGGGGGGGGGGGGTGCGGGGAACGGGCACGTACGTTCGCACTCCCGTAGTCGCTCGCTCTTCTTCCACTTCTCCCTCTAGTAGCGCCCCGCCGGGGGAGCCGAGGGTCCCCTCCCCGAGCGCCGGCGGTCGGACCCGACGGCTCGGACGCCGGCCGGGTGGGAGGCGAGGCGAGGCGAGGCGAGGCGAGGCGAGGCTGCCTCTCCGCCCTCCTGCCGGCCGGGAGAGAGGCTCGCTGCGAGCCCGGTGACCGGACGAGTCGAACAGACGAGTCCCAGGCGGCCCGCGGCCCGCGGCCCCCGCCCCCCGCCGCCGCCGCCGCCGCCGCCGCCGCCGCCGCCGCCGCCGCCGCCACCACCACCACCACCCCCGGGACGGACGACGCCGTCTCCCCCCGGCCTACCTCTGCTACCGTCTTCCCGCCATTATTTTGCGTTCCCGCGCCGACACGAGCGACCGCCCGCATGCGAACCGGCCGTACTTTCCCGCCCGCCCGGCCCCCTTGTGGGCGGAGCCCCCGCCCACACGCCCGCGCCTGCGCACCTCCTCGGGAGGTCGGGGCCGTGTCCGCCGGTCGGGTGGAGGGGGACCGGGGGGAGGGGGGGGGTTACGCGGCGCCCTGGCTAAGGTGTCCCGGCGTTGATGTGGCGCGCGAGCGAGCCGCACGCGAATGGGGTTTCAAAGACCCAGCTTGAAACCGCCTAAAGAGTTACCTAGCCCCCGAGCGAAAAGGAAACGAAACCAAAACGTTCAAACCGGCAAACGAACGGGCACACTGAGGGACAGAGAAATAAACAAAGAAGCAAACACGAGACGAACGCTGCCGTAAGAAGTCGCTGCCGAGCGGCTCGAAAGCAGCGGAACGGTCTACCCGGCCCCAGAGGGCTCCGAGCCGTTCACACGGCGGAGACCGCTAGAGGTCGCTGGCCCGGCCCTCTCCGGGCTGGCAGGCCACGTCTACCCGGCCAGGACGGACTTGGTCGCTCCGCGGCGGCGGCTGGAGGCCTCTGCGGGAGCCGCTCCCGGTCGCCGGACCGGACGGCCCGGCCGCTCCGTGCACGCACAGGACTCGGTCTCACTGCAGCCGCGGCTGGAGGCCTCTGCAGGACCGGCTCGGGCTCTCCGGAACGGTCGGCCCGGCCCCTGCGTGCCCAAAAAGGACTCGGTCACACAGCAGCAGTGGCTGGAGGCCTCTGCAGGACCGGCTCGGGCCCTCCGGAACGGTCGGCCCGGCCCCTGAGTGCCCCAAAAGGACTCGGTCACACTGCAGGTGCGGCTGGAGGCCGCTGCTGGACCGGCTCCGGCTCTCCGGAATGGTCAGCCCGGCCCCTGTGTGCTCCCACAGGACTCGGTGTCACTGCAGGTGTGGCTGGTGGCCTGTACAGGACCGACTCTGGGTCAGCGGACCGGTCGGCCCGGCCCCTGCGTGCCTCCACAGGACTCGGTCTCACTACAGGTGCGGCTGGAGGCCTGTACAGGCCCGTCTCAGGCTTGCTGGACCGGTCAACCCAACCCCTGCGTGCCCCCACCCGACTCGGCCACCTCCGCAGCTGCGGCTGGAGCATGCTGAAGGAGCCGATCCCGGTCACCGGACCGCTCGCACCGGCCCCTGCGGGCCCGCACAGCACTCGGTCACACTAAAGCCGCAGCTGGAGGCCTCTGCAGGACCGGCTCCGGCTCTCCGGAACGGTCAGCCCGGCCCCTGCGTGCCCAAACAGGACTCGGTCACACAGCAGCCGTGGCTGGAGGCCTCTGCAGGACCGGCTTGGGCTCGCCGTACCGGTCGGCCCAGCCCCTGTGTGCCCCACAGGACTCGGTTTCATTGCAGGTGCGGCTGCAGGCCTCTGCAGGACTGGCTCGGGCCCTCCGGAACGGTCAGCCCGGCACCTGCGTGCCCCCACCTGACTCGGCCACCACCGCAGCTGCGGCTGGAGCATGCTGAAGGAGCCGGTCCCGGTCGCCGGACCGCTCAGACCGGCCCGTGCGGGCCCGCACAGCACTCGGTCACACTAAAGCCGCAGCTGGCTCCCTCTGCACGACCGGCTCGGACACGCCAGACCGGTCAGACCGGCCCCTGGGGACCCCCACAACACTTGGTCACACTAAAGCCGCAGCTGGAGGCCTCTGCAGGACCGGCTCGGGCTTGCCAGACCGGTCGGCCCAGCCCCTGCGTGCCCCACAGGACTCGGTCTCACTGCAGGTGCGGCTGCAGGCCTCTGCAGGACCGGCTCGGGCTCTCCGGAACGGTCAGCCCGGCCCCTGCGTGCCCCCACCCGACTCGGCCTCCTCCGCAGCTGCGGCTGGAGCATGCTGAAGGAGCCGGTCCCGGTCGCCGGACCGCTCGGACCGGCCCCTGCGGGCCCGCACAGCACTCGGTCACACTAAAGCCGCAGCTGGCGCCCTCTGCACGACCGGCTCGGGCTCGCCAGACCGGTCGGACCGGCCCCTGGGGACCCCCACAACACTTGCTCACACTAAAGCCGCAGCTGGAGGCCTCTGCAGGACCGGCTCGGGCTCTCCGGAACGGTCAGCCCGGCCCCTGCGTGCCCAAACAGGACTCGGTCACACAGCAGCCGTGGCTGGAGGCCTCTGCAGGACCGGCTTGGGCTCGCCGTACCGGTCGGCCCAGCCCCTGTGTGCCCCACAGGACTCGGTTTCATTGCAGGTGCGGCTGCAGGCCTCTGCAGGACTGGCTCGGGCCCTCCGGAACGGTCAGCCCGGCCCCTGCGTGCCCCCACCCGACTCGGCCACCTCCGCAGCTGCGGCTGGAGCATGCTGAAGGAGCCGGTCCCGGTCGCCGGACCGGTCGGAACGGCCCCTACGGGCCCGCACAGCACTCGGTCACACTAAAGCCGCAGCTGGCGCCCTCTGCAGGACCGGCTCGGGCTCTCCGGAACGGTCAGCCCGGCCCCTGCGTGCCCAAACAGGACTCGGTCACACAGCAGGTGCGGCTGGTGGCCTGTACAGGACCAGCTCAGGCTCGCCGGACTGGTCACCCGGACCCCTGCGTGCCCCCACCGGACTCGGCCTCCTCCGCAGCTGTGGCTGGAGCATGCTGAAGGAGCCGGTCCGGGTCGCCGGACCGCTCAGACCGGCCCGTGCGGGCCCGCACAGCACTCGGTCACACTAAAGCCGCAGCTGGCTCCCTCTGCACGACCGGCTCGGGCACGCCAGACCGGTCAGACCGGCCCCTGGGGACCCCCACAACACTTGGTCACACTAAAGCCGCAGCTGGAGGCCCCTGCAGGACCGGCTCGGGCTTGCCAGACCGGTCGGCCCTGCCCCTGTGTGCCCCAACAGGACTCGGTCTCACTACAGGTGCGGCTGGAGGCCTGTACAGGACAGTCTCAGGCTTGCTGGACCGGTCAACCCAACCCCTGCGTGCCCCCACCGGACTCGGCCACCTCCGAGGCTGCGGCTGGAGCATGCTAAAGGAGCCGGTCCTGGTCGCCGGACCGCTCAGACCGGCCCGTGCGGGCCCGCACAGCACTCGGTCACACTAAAGCCGCAGCTGGAGGCCTCTGCAGGACCGGCTCGGGCTTGCCAGACCGGTCGGCCCAGCCCCTGCGTGCCCCACAGGACTCGGTTTCATTGCAGGTGCGGCTGCAGGCCTCTGCAGGACCGGCTCGGGCTCTCCGGAACGGTCAGCCCGGCCCCTGCGTGCCCCCACCCGACTGGGCCACCTCCGCAGCTGTGGCTGGAGCATGCTGAAGGAGCCGGTCCCGGTCGCCGGACCGGTCGGAACGGCCCCTGCGGGCCCGCACTGCACTCGGTCACACTAAAGCCGCAGCTGGCGCCCTCTGCAGGACCGGCTCGGGCTCGCCAGACCGGTCGGACCGGCCCCTGTGGACCCCCACAACACTTGGTCACACTAAAGCCGCAGCTGGAGGCCTCTGCAGGACCGGCTCGGGCTCTCCGGAACAGTCAGCCCGGCCCCTGCGTGCCCAAACAGGACTCGGTCTCACTGCAGGTGCGGCTGGTGGCCTGTACAGGACCAGCTCAGGCTCGCCGGACCGGTCACCCGGACCCCTGCGTGCCCCCACCGGACTCGGCCTCCTCCGCAGCTGTGGCTGGAGCATGCTGAAGGAGCCGGTCCTGGTCGCCGGACCGCTCAGACCGGCCCGTGCGGGCCCGCACAGCACTCGGTCACACTAAAGCCGCAGCTGGCTCCCTCTGCACGACCGGCTCGGGCACGCCAGACCGGTCGGACCGGCCCCTGGGGACCCCCACAACACTTGGTCACACTAAAGCCGCAGCTGGAGGCCTCTGCAGGACCGGCTCGGGCTCTCCGGAACTGTCGGCCCGGCCCCTGTGTGCCCAACAGGACTCGGTCTCACTGCAGGTGCGGCTGGTGGCCTGTACAGGACCAGCTCAGGCTCGCCGGACTGGTCACCCGGACCCCTGCGTGCCCCCACCGGACTCGGCCTCCTCCGCAGCTGTGGCTGGAGCATGCTGAAGGAGCCGGTCCCGGTCGCCGGACCGCTCAGACCGGCCCGTGCGGGCCTGCACAGCACTCGGTCACACTAAAGCCGCAGCGGGCTCCCTCTGCACGACCGGCTCGGGCTCGCCAGACCGGTCAGACCGGCCCCTGGGGACCCCCACTACACTTGGTCACACTAAAGCCGCAGCTGGAGGCCTGTGCAGGACCGGCTCGGGCTTGCCAGACCGGTCGGCCCAGCCCCTGCGTGCCCCACAGGACTCGGTTTCATTGCAGGTGCGGCTGCAGGCCTCTGCAGGACCGGCTCGGGCTCTCCGGAACGGTCAGCCCAGCCCCTGCGTGCTTCCACCCGACTCGGCCTCCTCCGCAGCTGCAGCTGGAGCATGCTGAAGGAGCCGGTCCCGGTCGCCGGACCGCTCGGACCGGCCCCTGCGGGCCCGCACAGCACTCGGTCACACTAAAGCCGCAGCTGGCGCCCTCTGCACGACCGGCTCGGGCTCGCCAGACCGGTCGGACCGGCCCCTGGGGACCCCCACAACACTTGGTCACACTAAAGCCGCAGCTGGAGGCCTCTGCAGGACCGGCTCGGGCCCTCCGGAATGGTCGGCCTGGCCCTTGTGTGCCCCACAGGACTCGGTCTCACTGCAGGTGCGGCTGGTGGCCTGTACAGGACCAGCTCAGGCTCGCCGGACTGGTCACCCAGACCCCTGCGTGCCCCCACCGGACTCGGCCTCCTCCGCAGCTGTGGCTGGAGCATGCTGAAGGAGCCGGTCCCGGTCGCCGGACCGCTCAGACCGGCCCGTGCGGGCCCGCACAGCACTCGGTCACACTAAAGCCGCAGCTGGCTCCCTCTGCAGGACCGGCTCGGGCTCGCCAGACCGGTCGGACCGGCCCATGGGGACCCCCACAACACTTGGTCACACTAAAGCCGCAGCTGGAGGCCTCTGCAGGACCGGCTCGGGCTCTCCGGAACTGTCGGCACGGCCCCTGTGTGCCCCACAGGACTCGGTCTCACTGCAGGTGCGGCTGGTGGCCTCTGCAGGACCGGCTCGGGCCCTCCGGAATGGTCGGCCTGGCCCCTGTGTGCCCCACAGGACTCGGTCTCACTGCAGGTGCGGCTGGTGGCCTGTACAGGACCAGCTCAGGCTCGCCGGACTGGTCACCCGGACCCCTGCGTGCCCCCACCGGACTCGGCCTCCTCCGCAGCTGCGGCTGGAGCATGCTGAAGGAGCCGGTCCCGGTCGCCGGACCGCTCAGACCAGCCCGTGCGGGCCCGCACAGCACTCGGTCACACTAAAGCCGCAGCTGGCTCCCTCTGCACGACCGGCTCGGGCACGCCAGACCGGTCAGACCGGCCCCTGGGGACCCCCACAACACTTGGTCACACTAAAGCCGCAGCTGGAGGCCTCTGCAGGACCGGCTCGGGCTTGCCAGACCGGTCAGCCCAGCCCCTGCGTGCCCCACAGGACTCGGTCTCACTGCAGGTGCGGCTGCAGGCCTCTGCAGGACCGGCTCGGGCTCTCCGGAACGGTCAGCCCGGCCCCTGCGTGCCCCCACCCGACTCGGCCTCCTCCGCAGCTGCGGCTGGAGCATGCTGAAGGAGCCGGTCCCGGTCGCCGGACCGCTCGGACCGGCCCCTGCGGGCCCGCACAGCACTCGGTCACACTAAAGCCGCAGCTGGCGCCCTCTGCAGGACCGGCTCGGGCTCGCCAGACCGGTCGGACCGGCCCCTGGGGACCCCCACAACACTTGGTCACACTAAAGCCGCAGCTGGAGGCCTCTGCAGGACCGGCTCGGGCTCTCCGGAACTGTCGGCCCGGCCCCTGTGTGCCCCACAGGACTCGGTCTCACTGCAGGTGCGGCTGGTGGCCTCTGCAGGACCGGCTCGGGCCCTCCGGAATGGTCGGCCTGGCCCCTGTGTGCCCCACAGGACTCGGCCTCCTCCGCAGCTGTGGCTGGAGCATGCTGAAGGAGCCGGTCCCGGTCGCCGGACCGCTCAGACCGGCCCGTGCGGGCCCGCACAGCACTCGGTCACACTAAAGCCGCAGCGGGCTCCCTCTGCACGACCGGCTCGGGCTCGCCAGACCGGTCAGACCGGCCCCTGGGGACCCCCACAACACTTGGTCACACTAAAGCCGCAGCTGGAGGCCTCTACAGGACCGGCTCGGGCTCTCCGGAACTGTCGGCCCGGCCCCTGTGTGCCCCACAGGACTCGGTCTCACTGCAGGTGCGGCTGGTGGCCTGTACAGGACCAGCTCAGGCTCGCCGGACTGGTCACCCGGACCCCTGCGTGCCCCCACCGGACTCGGCCTCCTCCTCAGCTGTGGCTGGAGCATGCTGAAGGAGCCGGTCCCGGTCGCCGGACCGCTCAGACCGGCCCGTGCGGGCCCGCACAGCACTCGGTCACACTAAAGCCGCAGCGGGCTCCCTCTGCACGACCGGCTCGGGCTCGCCAGACCGGTCAGACCGGCCCCTGGGGACCCCCACTACACTTGGTCACACTAAAGCCGCAGCTGGAGGCCTCTGCAGGACCGGCTCGGGCTTGCCAGACCGGTCGGCCCAGCCCCTGCGTGCCCCACAGGACTCGGTTTCATTGCAGGTGCGGCTGCAGGCCTCTGCAGGACCGGCTCGGGCTCTCCGGAACGGTCAGCCCAGCCCCTGCGTGCCCCCACCCGACTCGGCCTCCTCCGCAGCTGCGGCTGGAGCATGCTGAAGGAGCCGGTCCCGGTCGCCGGACCGCTCGGACCGGCCCCTGCGGGCCCGCACAGCACTCGGTCACACTAAAGCCGCAGCTGGCGCCCTCTGCAGGACCGGCTCGGGCTCGCCAGACCGGTCGGACCGGCCCCTGGGGACCCCCACAACACTTGGTCACACTAAAGCCGCAGCTGGAGGCCTCTGCAGGACCGGCTCGGGCTCTCCGGAACTGTCGGCCCGGCCCCTGTGTGCCCCACAGGACTCGGTCTCACTGCAGGTGCGGCTGGTGGCCTCTGCAGGACCGGCTCGGGCTCTCCGGAACGGTCAGCCCGGCCCCTGCGTGCCCCCACCCGACTCGGCCTCCTCCGCAGCTGCGGCTGGAGCATGCTGAAGGAGCCGGTCCCGGTCGCCGGACCGCTCGGACCGGCCCCTGCGGGCCCGCACAGCACTCGGTCACACTAAAGCCGCAGCTGGCGCCCTCTGTAGGACCGGCTCGGGCTCGCCAGACCGGTCGGACCGGCCCCTGGGGACCCCCACAACACTTGCTCACACTAAAGCCGCAGCTGGAGGCCTCTGCAGGACCGGCTCGGGCTCTCCGGAACGGTCAGCCCGGCCCCTGCGTGCCCAAACAGGACTCGGTCACACAGCAGCCGTGGCTGGAGGCCTCTGCAGGACCAGCTTGGGCTCGCCGTACCGGTCGGCCCAGCCCCTGTGTGCCCCACAGGACTCGGTTTCATTGCAGGTGCAGCTGCAGGCCTCTGCAGGACTGGCTCGGGCCCTCCGGAACGGTCAGCCCGGCCCCTGCGTGCCCCCACCCGACTCGGCCACCTCCGCAGCTGCGGCTGGAGCATGCTGAAGGAGCCGGTCCCGGTCGCCGGACCGGTCGGAACGGCCCCTACGGGCCCGCACAGCACTCGGTCACACTAAAGCCGCAGCTGGCGCCCTCTGCAGGACCGGCTCGGGCTCTCCGGAGCGGTCAGCCCGGCCCCTGCGTGCCCAAACAGGACTCGGTCACACAGCAGGTGCGGCTGGTGGCCTGTACAGGACCAGCTCAGGCTCGCCGGACTGGTCACCCGGACCCCTGCGTGCCCCCACCGGACTCGGCCTCCTCCGCAGCTGTGGCTGGAGCATGCTGAAGGAGCCGGTCCTGGTCGCCGGACCGCTCAGACCGGCCCGTGCGGGCCCGCACAGCACTCGGTCACACTAAAGCCGCAGCTGGCTCCCTCTGCACGACCGGCTCGGGCACGCCAGACCGGTCAGACCGGCCCCTGGGGACCCCCACAACACTTGGTCACACTAAAGCCGCAGCTGGAGGCCTCTGCAGGACCGGCTCGGGCTTGCCAGACCGGTCGGCCCAGCCCCTGCGTGCCCCACAGGACTCGGTTTCATTGCAGGTGCGGCTGCAGGCCTCTGCAGGACCGGCTCGGGCTCTCCGGAACGGTCAGCCCAGCCCCTGCGTGCCCCCACCCGACTCGGCCTCCTCCGCAGCTGCGGCTGGAGCATGCTGAAGGAGCCGGTCCCGGTCGCCGGACCGCTCGGACCGGCCCCTGCGGGCCCGCACAGCACTCGGTCACACTAAAGCCGCAGCTGGCGCCCTCTGCAGGACCGGCTCGGGCTCGCCAGACCGGTCGGACCGGCCCCTGGGGACCCCCACAACACTTGGTCACACTAAAGCCGCAGCTGGAGGCCTCTGCAGGACCGGCTCGGGCTCTCCGGAACGGTCAGCCCGGCCCCTGCGTGCCCAAACAGGACTCGGTCACACAGCAGGTGCGGCTGGTGGCCTGTACAGGACCAGCTCAGGCTCGCCGGACTGGTCACCCGGACCCCTGCGTGCCCCCACCGGACTCGGCCTCCTCCGCAGCTGTGGCTGGAGCATGCTTAAGGAGCCGGTCCTGGTCGCCGGACCGCTCAGACCGGCCCGTGCGGGCCCGTACAGCACTCGGTCACACTAAAGCCGCAGCTGGCTCCCTCTGCACGACCGGCTCGGGCACGCCAGACCGGTCAGACCGGCCCCTGGGGACCCCCACAACACTTGGTCACACTAAAGCCGCAGCTGGAGGCCTCTACAGGACCGGCTCGGGCTCTCCGGAACTGTCGGCCCGGCCCCTGCGTGCCCCACAGGACTCGGTCTCACTGCAGGTGCGGCTGGTGGCCTGTACAGGACCAGCTCAGGCTCGCCGGACTGGTCTCCCGGACCCCTGCGTGCCCCCACCGGACTCGGCCTCCTCCGCAGCTGTGGCTGGAGCATGCTGAAGGAGCCGGTCCCGGTCGCCGGACCGCTCAGACCGGCCCGTGCGGGCCCGCACAGCACTCGGTCACACTAAAGCCGCAGCGGGCTCCCTCTGCACGACCGGCTCGGGCTCGCCAGACCGGTCAGACCGGCCCCTGGGGACCCCCACTACACTTGGTCACACTAAAGCCGCAGCTGGAGGCCTCTGCAGGACCGGCTCGGGCTTGCCAGACCGGTCGGCCCAGCCCCTGCGTGCCCCACAGGACTCGATTTCATTGCAGGTGCGGCTGCAGGCCTCTGCAGGACCGGCTCGGGCTCTCCGGAACGGTCAGCCCGGCCCCTGCGTGCCCCCACCCGACTCGGCCTCCTCCGCAGCTGTGGCTGGAGCATGCTGAAGGAGCCGGTCCCGGTCGCCGGACCGCTCAGACCGGCCCGTGCGGGCCCGCACAGCACTCGGTCACACTAAAGCCGCAGCGGGCTCCCTCTGCACGACCGGCTTGGGCTCGCCAGACCGGTCAGACCGGCCCCTGGGGACCCCCACAACACTTGGTCACACTAAAGCCGCAGCTGGAGGCCTCTGCAGGACCGGCTCGGGCTTGCCAGACCGGTCGGCCCAGCCCCTGCGTGCCCCACAGGACTCGGTTTCATTGCAGGTGCGGCTGCAGGCCTCTGCAGGACCGGCTCGGGCTCTCCGGAACGGTCAGCCCAGCCCCTGCGTGCCCCCACCCGACTCGGCCTCCTCCGCAGCTGCGGCTGGAGCATGCTGAAGGAGCCGGTCCCAGTCGCCGGACCGCTCGGACCGGCCCCTGCGGGCCCGAACAGCACTCGGTCACACTAAAGCCGCAGCTGGCGCCCTCTGCAGGACCGGCTCGGGCTCGCCAGACCGGTCGGACCGGCCCCTGGGGACCCCCACTACACTTGGTCACACTAAAGCCGCAGCTGGAGGCCTCTGCAGGACCGGCTCGGGCTCTCCGGAACTGTCGGCCCGGCCCCTGTGAGCCCCACAGGACTCGGTCTCACTGCAGGTGCGGCTGGTGGCCTCTGCAGGACCGGCTCGGGCCCTCCGGAATGGTCGGCCTGGCCCCTGTGTGCCCCACAGGACTCGGTCTCACTGCAGGTGCGGCTGGTGGCCTGTACAGGACCAGCTCAGGCTCGCCGGACTGGTCACCCGGACCCCTGCGTGCCCCCACCGGACTCGGCCTCCTCCGCAGCTGTGGCTGGAGCATGCTGAAGGAGCCGGTCCCGGTCGCCGGACCGCTCAGACCGGCCCGTGCGGGCCCGCACAGCACTCGGTCACACTAAAGCCGCAGCTGGCTCCCTCTGCACGACCGGCTCGGGCACGCCAGACCGGTCAGACCGGCCCCTGGGGACCCCCACTACACTTGGTCACACTAAAGCCGCAGCTGGAGGCCTCTGCAGGACCGGCTCGGGCTTGCCAGACCGGTCGGCCCAGCCCCTGCGTGCCCCACAGGACTCGGTTTCATTGCAGGTGCGGCTGGAGGCCTCTGCAGGACCGGCTCGGGCTCTCCGGAACGGTCAGCCCGGCCCCTGCGTGCCCCCACCCGACTCGGCCACCTCCGCAGCTGCGGCTGGAGCATGCTGAAGGAGCCGGTCCCGGTCGCCGGACCGCTCGGAACGGCCCCTGCGGGCCCGCACAGCACTCGGTCACACTAAAGCCGCAGCTGGCGCCCTCTGCACGACCGGCTCGGGCTCGCCAGACCGGTCGGACCGGCCCCTGGGGACCCCCACAACACTTGCTCACACTAAAGCCGCAGCTGGAGGCCTCTGCAGGACCGGCTCGGGCTCTCCGGAACGGTCAGCCCGGCCCCTGCGTGCCCAAACAGGACTCGGTCTCACTGCAGGTGCGGCTGGTGGCCTGTACAGGACCAGCTCAGGCTCGCCGGACTGGTCACCCGGACCCCTGCGTGCCCCCACCGGACTTGGCCTCCTCCGCAGCTGTGGCTGGAGCATGCTGAAGGAGCCGGTCCTGGTCGCCGGACCGGTCAGACCGGCCCGTGCCGGCCCGCACAGCACTCGGTCACACTAAAGCCGCAGCTGGCTCCCTCTGCACGACCGGCTCGGGCACGCCAGACCGGTCAGACCGGCCCCTGGGGACCCCCACAACACTTGGTCACACTAAAGCCGCAGCTGGAGGCCTCTGCAGGACCGGCTCGGGCTCTCCGGAACTGTCGGCCCGGCCCCTGTGTGCCCCACAGGACTCGGTCTCACTGCAGGTGCGGCTGGTGGCCTCTGCAGGACCGGCTCGGGCCCTCCGGAACGGTCAGCCCGGCCCCTGCGTGCCCCCACCCGACTCGGCCTCCTCCGCAGCTGCGGCTGGAGCATGCTGAAGGAGCCGGTCCCGGTCGCCGGACCGCTCGGACCGGCCCCTGCGGGCCCGCACAGCACTCGGTCACACTAAAGCCGCAGCTGGCGCCCTCTGCAGGACCGGCTCGGGCTCGCCAGACCGGTCGGACCGGCCCCTGGGGACCCCCACAACACTTGGTCACACTAAAGCCGCAGCTGGAGGCCTCTGCAGGACCGGCTCGGGCTCTCCGGAACTGTCGGCCCGGCCCCTGTGTGCCCCACAGGACTCGGTCTCACTGCAGGTGCGGCTGGTGGCCTGTACAGGACTAGCTCAGGCTCGCCGGACTGGTCACCCGGAGCCCTGCGTGCCTCCACCGGACTCGGCCTCCTCCGCAGCTGCGGCTGGAGCATGCTGAAGGAGCCGGTCCCGGTCGCCGGACCGCTCGGACCGGCCCCTGCGGGCCCGCACAGCACTCGGTCACAGTAAAGCCGCAGCTGGCGCCCTCTGCAGGACCGGCTCGGGCTCGCCAGACCGGTCGGACCGGCCCCTGGGGACCCCCACAACACTTGGTCACACTAAAGCCGCAGCTGGAGGCCTCTGCAGGACCGGCTCGGGCTCTCCGGAACTGTCGGCCCGGCCCCTGTGTGCCCCACAGGACTCGGTCTCACTGCAGGTGCGGCTGGTGGCCTGTACAGGACTAGCTCAGGCTCGCCGGACTGGTCACCCGGAGCCCTGCGTGCCTCCACCGGACTCGGCCTCCTCCGCAGCTGTGGCTGGAGCATGCTGAAGGAGCCGGTCCTGGTCGCCGGACCGGTCAGACCGGCCCGTGCGGGCCCGCACAGCACTCGGTCACACTAAAGCCGCAGCTGGCTCCCTCTGCACGACCGGCCCGGGCTCGCCAGACCGGTCAGACCGGCCCCTGGGGACCCCCACAACACTTGGTCACACTAAAGCCGCAGCTGGAGGCCTCTGCAGGACCGGCTCGGGCTTGCCAGACCGGTCGGCCCAGCCCCTGCGTGCCCCACAGGACTCGGTTTCATTGCAGGTGCGGCTGCAGGCCTCTGCAGGACCGGCTCGGGCTCTCCGGAACGGTCAGCCCGGCCCCTGCGTGCCCCCACCCGACTCGGCCACCTCCGCAGCTGCGGCTGGAGCATGCTGAAGGAGCCGGTCCCGGTCGCCGGACCGCTCAGACCGGCCCGTGCGGGCCCGCACAGCACTCGGTCACACTAAAGCCGCAGCTGGCTCCCTCTGCACGACCGGCTCGGGCTCGCCAGACCGGTCAGACCGGCCCCTGGGGACCCCCACTACACTTGGTCACACTAAAGCCGCAGCTGGAGGCCTCTGCAGGACCGGCTCGGGCTTGCCAGACCGGTCGGCCCAGCCCCTGCGTGCCCCACAGGACTCGGTTTCATTGCAGGTGCGGCTGCAGGCCTCTGCAGGACCGGCTCGGGCTCTCCGGAACGGTCAGCCCGGCCCCTGCGTGCCCCCACCCGACTCGGCCTCCTCCGCAGCTGCGGCTGGAGCATGCTGAAGGAGCTGGTCCCGGTCGCCGGACCGCTCGGACCGGCCCCTGCGGGCCCGCACAGCACTCGGTCACACTAAAGCCGCAGCTGGCGCCCTCTGCAGGACCGGCTCGGGCTCGCCAGACCGGTCGGACCGGCCCCTGGGGACCCCCACAACACTTGGTCACACTAAAGCCGCAGCTGGAGGCCTCTGCAGGACCGGCTCGGGCTCTCCGGAACTGTCGGCCCGGCCCCTGTGTGCCCCACAGGACTCGGTCTCACTGCAGGTGCGGCTGGTGGCCTGTACAGGACTAGCTCAGGCTCGCCGGACTGGTCACCCGGAGCCCTGCGTGCCTCCACCGGACTCGGCCTCCTCCGCAGCTGCGGCTGGAGCATGCTGAAGGAGCCGGTCCCGGTCGCCGGACCGCTCGGACCGGCCCCTGCGGGCCCGCACAGCACTCGGTCACACTAAAGCCGCAGCTGGCGCCCTCTGCAGGACCGGCTCGGGCTCGCCAGACCGGTCGGACCGGCCCCTGGGGACCCCCACAACACTTGGTCACACTAAAGCCGCAGCTGGAGGCCTCTGCAGGACCGGCTCGGGCTCTCCGGAACTGTCGGCCCGGCCCCTGTGTGCCCCACAGGACTCGGTCTCACTGCAGGTGCGGCTGGTGGCCTGTACAGGACTAGCTCAGGCTCGCCGGACTGGTCACCCGGAGCCCTGCGTGCCTCCACCGGACTCGGCCTCCTCCGCAGCTGTGGCTGGAGCATGCTGAAGGAGCCGGTCCTGGTCGCCGGACCGGTCAGACCGGCCCGTGCGGGCCCGCACAGCACTCGGTCACACTAAAGCCGCAGCTGGCTCCCTCTGCACGACCGGCCCGGGCTCGCCAGACCGGTCAGACCGGCCCCTGGGGACCCCCACAACACTTGGTCACACTAAAGCCGCAGCTGGAGGCCTCTGCAGGACCGGCTCGGGCTTGCCAGACCGGTCGGCCCAGCCCCTGCGTGCCCCACAGGACTCGGTTTCATTGCAGGTGCGGCTGCAGGCCTCTGCAGGACCGGCTCGGGCTCTCCGGAACGGTCAGCCCGGCCCCTGCGTGCCCCCACCCGACTCGGCCACCTCCGCAGCTGCGGCTGGAGCATGCTGAAGGAGCCGGTCCCGGTCGCCGGACCGCTCAGACCGGCCCGTGCGGGCCCGCACAGCACTCGGTCACACTAAAGCCGCAGCTGGCTCCCTCTGCACGACCGGCTCGGGCTCGCCAGACCGGTCAGACCGGCCCCTGGGGACCCCCACTACACTTGGTCACACTAAAGCCGCAGCTGGAGGCCTCTGCAGGACCGGCTCGGGCTTGCCAGACCGGTCGGCCCAGCCCCTGCGTGCCCCACAGGACTCGGTTTCATTGCAGGTGCGGCTGCAGGCCTCTGCAGGACCGGCTCGGGCTCTCCGGAACGGTCAGCCCGGCCCCTGCGTGCCCCCACCCGACTCGGTCACACAGCAGTAGTGGCTGGAGGCCTGTGCAGGACCGGCTCGGGCTCGCCGTACCAGTCGGCCCAGCCCCTGTGTGCCCCGCAGGACTCGGTCTCACTGCCGGTTCGGCTGGTGGCCTGTACAGGACTGGCTCAGGCTTTCCTTACCGGTCACCCTGACCCCTGCGTGCCCCCACCGGACTCGGCCACCTCCGCAGCTGTGGCTGGAGCATGCTGAAGGAGTTGGTCCCCATCGCCGGACCGGTCTGATCGGCCCCTGCGGGCCCGCACAGCACTCGGGCACACTAAAACCGCAGCTGGTGCCCTCTGCAGGACCGTCTCGGGCTCGCCAGACCGGTCGGACCGGCCCCTGGGGACCCCCACAACGCTCTGTCACACTAAAGCCGCAGCTGGAGGCCTCTGCAAGACCGGCTCGGGCTTGCCAGACCGGTCAGCCTGGCCTTTGCGAGCCCCAACAGGACTCGGTCAAACTGCAGCAGTGGCTGGAGGCCTCTGCTGGACTGGCTCCGGCTCTCTGGAACGGTCAGCCCGGCCCCTGTGTGCCCCCACAGGACTAGGTCTCACTGCAGGTGCGGCTGCAGGCCTCTGCAGGACCGGCTCGGGCTCGCCGGACCGGTCGGCCCGGCCCCTGCATGCCCCAACAGGACTCGGTCACACTGCAGCCGTGGCTGGAGGCCTACGCAGGACCGGCTCGGACCCTCCGGACCGGTCGGCCCGGCCCTTGCAGGCCCCGAAAAAAATCAGTCTCATGGCAGCCGCAGCTGGAGATCGCTGCCGGGGCGTCTCGTGTACTCCTACACGGTCTACTTGGCCCCGACCGACTCGCACACTCCGCGGCGGCAGCTCGATGTCTCTGCCGGGGAGGCTCGAAAACGGCGGAACGGTCTACCAGGCCCCGGCAGACTTCCTCCGGCTCGGTCGCTCCGCGGCGGCGGCTAGAGGTCGCTGCCGGGGCGGCTGGGTCACGGCGGAACGGTCTACCCGGCCCCGGAGGGTTCCGAGCCGCTTCACCTGTTGTGGCCGCTAGAGGTCGCTGCCGCGTAAATGTCGGGTACGCCGGCCACGTCTACCCGGCCAGGACGGACTTGGTCGCTTCGAAGCGGCGGCGGCTGGAGGTTGCTGCGGGAGCCGCTCCCGGTCGCCGGACCGGTCGGCCCGGCCCCTGCGGGCGCCGAGCGACACGGTCGTTCGGCGGCGGCGGCTGGGTACCGTGGCAGGATCGGCTCGGGTTCGCCGGACCGGTGGTCCCGGCCCGACGGGCACGGGCCACTCGGCCGTTCCGCGGCAATGGGTGGAGACTCGTGTAAGACCGTCTCACGTTCGCCGGATCGGTCGGCCAGGCCCCGTCCGACTCGGTCGCTTCGCGGAGGTAGCTGGAGGTCGCTGCCGTGGCGGCTGGGGCACGGCGGGACGGTCTACCTTGCCCCGGCAGGCTGCGTCCGGCTCGGTCGCTCCGCGGCGGCAGCTAGAGGTCGCTGCCGGGGCGGCTGGGGCACGGCGGAAAGGTCTACCCGGCCCCGGCGGGCCCCGTCCGCCTCGGTCGCTCCGCGGCGGCTGCTAGGGGTCGCTGCCGGGCTGCTGGGGCACGGCGGAAAGGTCTACCCGGCCCCGGCGGGCCCCGTCCGGCTCGGTCGCTCCGCGGCGGCTGCTAGAGGTCGCTGCCGGGGCGGCTGGGGCACGGCGGAAAGGTCTACCCGGCCCCGGCGGGCCCCGTCCGGCTCGGTCGCTCCGCGGCGGCGTCTAGAGGTCGCTGCCGTGTCGACTCGGAAACGGCAGAACGGTCTACCGGGCCCCGGCAGGCTGCGTCCGGCTCGGTCGCTTCGCGGCGGCGGCTAGAGGTCGCTGCCGGGGCGGCTGGGGCACGGCGGGACGGTCTACCCGGGTCCGGCAGGCTTCGTCCGCCTCGGTCGCTCCGCGGAGGAGGCTAGGGGTCGCTGCCGGGGCGGTTCGGAAACGGCGGGACGGTCTACCCGGCTCCGGCGGTCCCCGTCCGGCTCGGTCTCTCCGCGGCGGCGTCTAGGGGTCGCTGCCGGGGCGTCTCGGAAGCGGCGGGACGGTCTACCGGGCTCCGGCAGGCTTCGTCCGGCTCGGTCGCTCCGCGGCGGCGGCTAGAGGTCGCTGCCGGGGCGGCTGGGTCACGGCGGAACGGTCTACCCGGCCCCGGAGGGTTCCGAGCCGCTTCACCTGTTGTGGCCGCTAGAGGTCGCTGCCGCGTAAATGTCGGGTACGCCGGCCACGTCTACCCGGCCAGGACGGACTTGGTCGCTTCGCGGCGGCGGCTGGAGGTTGCTGCGGGAGCCGCTCCCGGTCGCCGGACCGGTCGGCCCGGCCCCTGCGGGCGCCGAGCGACACGGTCGTTCGGCGGCGGCGGCTGGGGACCGTGGCAGGAGCGGCTCGGGCTCGCCGGACCGGTGGTCCCGGCCCCGTCCGACTCGGTCGCTTCGCGGAGGTGGCTGGAGGTCGCTGCCGTGGCGGCTGGGGCACGGCGGAACGGTCTACCCGGCTCCGGCGGGCTGCGTCCGCCTCGGTCGCTGCCGTGGCGGCTGCTAGGGGTCGCTGCCGGGGTGGCTGGGGCACGGCGGAACGGTCTACCCGGGTCCGGCGGGCCCCGTCCGCCTCGGTCGCTCCGCGGAGGAGGCTAGGGGTCGCTGCCGGGGCGTCTCGGAAACGGCGGGACGGTCTACCTTGCCCCGGCAGGCTTCGTCCGGCTCGGTCGCTCCGCGGCGGCGGCTAGAGGTCGCTGCCGTGTCGGCTCGGAAACGGCGGAACGGTCTACCCGGCCCCGGCAGGCTGCGTCCGGCTCGGTCGCTCCGCGGCGGCGGCTAGAGGTCGCTGCCGGGGCGGCTGGGAAACGGCGGGACGGTCTACCCGGCCCCGGCGGTCCCCGTCCGACTTGGTCGCTCCGCGGCGGCGGCTAGAGGTCGCTGCCGGGGCGTCTCGGAAACGGCGGGACGGTCTACCTTGCCCCGGCAGGCTTCGTCCGGCTCGGTCTCTCCGCGGCGGCGGCGGCTAGGGGTCGCTGCCGGGGCGGCTCGGAAACGGCGGCACGGTCTACCCGGCTCCGGCGGTCCCCGTCCGGCTCGGTCGCTCCGCGGCGGCGGCTAGGGGTCGCTGCCGGGGCGGCTGGGTCACGGCGGAACGGTCTACCCCGCCCCGGAGGGTTCCGAGCCGCTTCACCTGTTGTGGCCGCTAGAGGTCGCTGCCGCGTAAATGTCGGGTACGCCGGCCACGTCTACCCGGCCAGGACGGACTTGGTCGCTTCGCGGCGGCGGCTGGAGGTTGCTGCGGGAGCCGCTCCCGGTCGCCGGACCGGTCGGCCCGGCCCCTGCGGGCGCCGAGCGACACGGTCGTTCGGCGGCGGCGGCTGGGGACCGTGGCAGGAGCGGCTCGGGCTCGCCGGACCGGTGGTCCCGGCCCCGTCCGACTCGGTCGCTTCGCGGAGGTGGCTGGAGGTCGCTGCCGTGGCGGCTGGGGCACGGCGGAACGGTCTACCCGGCTCCGGCGGGCTGCGTCCGCCTCGGTCGCTGCCGTGGCGGCTGCTAGGGGTCGCTGCCGGGGTGGCTGGGGCACGGCGGAACGGTCTACCCGGGTCCGGCGGGCCCCGTCCGCCTCGGTCGCTCCGCGGAGGAGGCTAGGGGTCGCTGCCGGGGCGTCTCGGAAACGGCGGGACGGTCTACCTTGCCCCGGCAGGCTTCGTCCGGCTCGGTCGCTCCGCGGCGGCGGCTAGAGGTCGCTGCCGTGTCGGCTCGGAAACGGCGGAACGGTCTACCCGGCCCCGGCAGGCTGCGTCCGGCTCGGTCGCTCCGCGGCGGCGGCTAGAGGTCGCTGCCGGGGCGGCTGGGAAACGGCGGGACGGTCTACCCGGCTCCGGCGGGCCCCGTCCGACTTGGTCGCTCCGCGGAGGAGGCTAGGGGTCGCTGCCGGGGCGTCTCGGAAACGGCGGGACGGTCTACCTTGCCCCGGCAGGTTTCGTCCGGCTCGGTCTCTCCGCGGCGGCAGCGGCTAGGGGTCGCTGCCGGGGCGGCTCGGAAACGGCGGCACGGTCTACCCGGCTCCGGCGGTCCCCGTCCGGCTCGGTCGCTCCGCGGCGGCGGCTAGGGGTCGCTGCCGGGGCGGCTGGGGCACGGCGGAACGGTCTACCTGGTCCCGGCGGGCACCGTCCGGCTCGGTCTCTCCGCGGCGGCAGCGGCTAGGGGTCGCTGCCGGGGCGGCTCGGAAACGGCGGCACGGTCTACCCGGCTCCGGCGGTCCCCGTCCGGCTCGGTCGCTCCGCGGCGGCGGCTAGGGGTCGCTGCCGGGGCGGCTGGGGCACGGCGGAACGGTCTACCTGGTCCCGGCGGGCACCGTCCGGCTCGGTCTCTCCGCGGCGGCGGCGGCTAGGGGTCGCTGCCGGGGCGTCTCGGAAACGGCGGAACGGTCTACCCGGGTGCTACCGTCTCGCGCTCTCCGCGGCGGCGGCTAGAGGTCGCTGCCGGGGCGGCTTGCGATCCGCGTCCAGGTCTACCCCGTTTCGGATTGTCTTGGCCGCTCTGGCTGTGGGGGGGGCGCTACAGCTCCGGAGCTGCCAGAGGCGTCGCTGTAATTTTGTACCTCCAGTTACGTCGAGGTAAACCTCGGCTGCCGTCGGAGCCGCTGCCGGTAGTCGGCGCCTATGGGACTAGAACGTTTTTTTCGGATGCCTTATATGTTCGTCTGTAGGAGCGAGTGAGGACTCGGCTCCGGTAGTGGCGGTGAGCGGGCGCTCGCGAGCAGGGTTGACCGGCCGGCCGCCTAGAGAGGGGATCGGCGGCGGCGGCGGCGGCTTTCTCGGGCATCGGTTCGTTCGATCGGTCCGGTCGCTTCGGTTTGTCCGTCGCTCCTCATCCCGCAGCTCTGTCCTGGGCTAAGGCGGTTTTGCAGGCGAGCAGCGAAAAAAAGCCGGAGAAGGCGAGAGAGAGGCAAGAGGCAAGCCGGCTCCCGCGCCGCCAGGGCGAAGGCGAGAGAGAGAGGGAGAGAGAGACGAGAAGGGCACGGGCCGGTCTGCCGGCACCCGAACGTAGGATGGCCGGGGGCGTCCCCGGCGGGTCCCGCCGCGATGGAAGAGGGGGACCCGGAGGTCGTAGGTCGTGGCGGCGTCGCCTCGTCCTCCTTTCGCACCGCATTCTCACCCGCACGCGGGAGCCCCGGCCGATTCGTGGCGCTCCTCGGGCGCGTCGGGGAGGCTTCCCGGCGGGCCGGCTCTATCCCGCTCCCCGGCTCGTTCGGGGTGGCGTGGGGCGGGCCGGTGTTCAGGCACGGGCGAGCACCTCTCGTCGGACGTTGCCCACGCACACCCACCTGCACGTGCGCGTGCGGTCTTTCCGCCGCGCCTGGGGGAAGGGCTCGCGCCTTCTCCCTCCTTCCTTTCTCCTCCCCCCCACCCCCTTTCTCCCACCGATCGATGAGGCCACTCGGGTCGCGTCGGAGAGGGCCCCCGGCGGGCCGGCGCTCCGCGCTCCCTGTCCCAGGGAAGCCGCGGCGGCGTCCGGTGTTCAGGCACGGGCGGCCTCCTCTCCAGTTCGCTTCCCGTCGTTCGCGAGGTGAGGCGCTCGCCCGCTTGGGCCGAGGGCGGCGGCGGCGGCGGCTTCGGGGCGCGTGGCCTCGCCGTGCCGACTCGTCTGTCCGCCCGCCCTGTCGGTGCCCCAGGGCTCGCCCGACCGAATCCAGCTGTGTGACGGCCGAGCGGCCCCGCGAGCCGCAGGCGTACCCATTTCGTTGTGAGCGAAGCGTCGGCGCTGCCCTCGTTTCGGGGCCCGGCGAGTGCCGGCCGCGAGCAGCAAGCCGGCGGGGTGGCAACCGAGGGAAACCGCGGGGAACCGAGGCGAAGCGAGCAGCAGCAGAAGAAGAAGGAACGAGAAGACAACGGGGGGCTGCGCCCGGCCGAGCGGGCGAGCCCGGAGCAGCGCGGCGCGTCCCGCTCCGGATCCGTCGGGGTGTGGGGGCCGGGGGCGTCCGCCGGCCTCTCCTCCGCCTTCGGGCCGCCGCAGCCCGTGTCGGTTTTCCTGCCGCGTCCCCGCCCGCTGCGGAGCGTGCCGCCCCGGGAAAGGGTCTCCGATCGTGGGGTCGCGCCCGTCTCGAGGTCGCGTTCTCCTCTAGCACATCCGTCTCCGGCGGCGGGGCTTCGTTTCCCCGTCCGCTTCTCCGCCGGTCCCGGAGGGCGGGTCAGCCCCGGCCGGCCGTGCGGCGCGAGCGCGAGTCCGGCTCCCGCGGGGGGGGCCCGGAGCGTGCCGCCGAAAGCAGCTGCGCAGCGGTCCCCGCTCCTTCCCCGCGGGGGGGAGGTCGGCGGGGCCGCCCCGGGGATCGGGCGCGCCTCTCCGTCGTGGTCGGCGAGCGAGCGAGCGAGGGAACGACGGAGGGCCGCCCGCCCCGCCGAGAGGCGTTCGCCCCGGCGGCCGCCGCCGTCGACCCGGCAAGGGCCAGACGGGAAAGCCGAGCGAGCAGGCGAGAGAGAGAGAGAGGGAAGGAGCGAGAGCGGTCGGCGGCGGGCCGGGCCCGTCGGGTCGTGCCCCGTGGCGCGGCTACCTGGTTGATCCTGCCAGTAGCATATGCTTGTCTCAAAGATTAAGCCATGCATGTCTAAGTACACACGGGCGGTACAGTGAAACTGCGAATGGCTCATTAAATCAGTTATGGTTCCTTTGGTCGCTCCCCTCCCGTTACTTGGATAACTGTGGTAATTCTAGAGCTAATACATGCCGACGAGCGCCGACCTCCGGGGACGCGTGCATTTATCAGACCAAAACCAACCCGGGCTCGCCCGGCGGCTTTGGTGACTCTAGATAACCTCGAGCCGATCGCACGCCCCCGTGGCGGCGACGACCCATTCGAATGTCTGCCCTATCAACTTTCGATGGTACTGTCTGTGCCTACCATGGTGACCACGGGTAACGGGGAATCAGGGTTCGATTCCGGAGAGGGAGCCTGAGAAACGGCTACCACATCCAAGGAAGGCAGCAGGCGCGCAAATTACCCACTCCCGACCCGGGGAGGTAGTGACGAAAAATAACAATACAGGACTCTTTCGAGGCCCTGTAATTGGAATGAGTCCACTTTAAATCCTTTAACGAGGATCCATTGGAGGGCAAGTCTGGTGCCAGCAGCCGCGGTAATTCCAGCTCCAATAGCGTATATTAAAGTTGCTGCAGTTAAAAAGCTCGTAGTTGGATCTTGGGATCGAGCTGGCGGTCCGCCGCGAGGCGAGCTACCGCCTGTCCCAGCCCCTGTCTCTCGGCGCCCCCTCGATGCTCTTAACTGAGTGTCCCGCGGGGCCCGAAGCGTTTACTTTGAAAAAATTAGAGTGTTCAAAGCAGGCTGGCCGCCGGAATACTCCAGCTAGGAATAATGGAATAGGACTCCGGTTCTATTTTGTTGGTTTTCGGAAACGGGGCCATGATTAAGAGGGACGGCCGGGGGCATTCGTATTGTGCCGCTAGAGGTGAAATTCTTGGACCGGCGCAAGACGAACTAAAGCGAAAGCATTTGCCAAGAATGTTTTCATTAATCAAGAACGAAAGTCGGAGGTTCGAAGACGATCAGATACCGTCGTAGTTCCGACCATAAACGATGCCGACTCGCGATCCGGCGGCGTTATTCCCATGACCCGCCGGGCAGCTCCCGGGAAACCCAAGTCTTTGGGTTCCGGGGGGAGTATGGTTGCAAAGCTGAAACTTAAAGGAATTGACGGAAGGGCACCACCAGGAGTGGAGCCTGCGGCTTAATTTGACTCAACACGGGAAACCTCACCCGGCCCGGACACGGACAGGATTGACAGATTGAGAGCTCTTTCTCGATTCCGTGGGTGGTGGTGCATGGCCGTTCTTAGTTGGTGGAGCGATTTGTCTGGTTAATTCCGATAACGAACGAGACTCTGGCATGCTAACTAGTTACGCGACCCCCGAGCGGTCGGCGTCCAACTTCTTAGAGGGACAAGTGGCGTTCAGCCACCCGAGATTGAGCAATAACAGGTCTGTGATGCCCTTAGATGTCCGGGGCTGCACGCGCGCTACACTGACTGGCTCAGCTTGTGTCTACCCTACGCCGGCAGGCGCGGGTAACCCGTTGAACCCCATTCGTGATGGGGATCGGGGATTGCAATTATTCCCCATGAACGAGGAATTCCCAGTAAGTGCGGGTCATAAGCTCGCGTTGATTAAGTCCCTGCCCTTTGTACACACCGCCCGTCGCTACTACCGATTGGATGGTTTAGTGAGGTCCTCGGATCGGCCCCGGCGGGGTCGGCCACGGCCCTGCCGGAGCGTCGAGAAGACGGTCGAACTTGACTATCTAGAGGAAGTAAAAGTCGTAACAAGGTTTCCGTAGGTGAACCTGCCGAAGGATCATTACCGGGGCCGAGGCCGGGCGTCCGGCCGAGCCGTGGCACGAGCGCGCGCGGGCGCGCAGCCTTCCCTTCCCTTCCCCGAGCCCGCTCCGCGCGGAGCGCGGCTCCTCTCCCCCGGTCGAAACGGGGAAAGAAAAAAAAAACACCGCAAGTCGCTCCGCGCGCCTGCCGGCGAGAGAGAAGGGAGACGAGGGCGCGGAGCGCAGCTCCGGGGGGGGAGGCGCGTGTGGGGCGCTCCGGCGCTCCGGCGCGTCTCTCCCCCCGGCGCCGGTCCGCCGTCGGTCCGCACGCCGCGGGTCCGGTCCGTCCGGTCGCCTCGCCGGCGCGCGCCCGCGCGCGCGCGTCCCGCGGGCCTCGCCCGGGTCGCCGCGCTCCGGAGCGTCCCGCGGCCGAGTCCCGCTCCGACCGCGGGGTCGGGGTCGGGAGGTGGCGGCGGTGCGGAGGGTGGAAGGACGGCTCCCCGCTTCGTCGCTCGGCCGGAAACTCGCCACCGGCCCCCGCCGCTGTCGACGCCGGCACCCCGAGTCCGCTCGGAGGGAAGCCGCGCGGGCGGCCGCGCGCGGGGGAGGCGGCGGGCGGCGGGTCCGAGCGCGGGGCGCGGGAAGTCGGCCGCTTCCCCCGGCCTCACCCCCCACCCCCTTCGCCCGGCCCGTCGCGGGGACGGGGCCGGGTCGCGGGCGGCTGCGGAGCCGGCCGACTCCGGGCGAGCGCCGGAGGGACGCGCGCGCCGCGTACGCGCGGCAGGCGCGAGGTGCCCCGGGCGGCTTCGGTCCCGCGCGGGCGGTCCGAGCCTCGCGGCTCCTCCCGGGTGCAGCTGCCGCCCGGCGCCGGGTTGCCGAGGGAAACCCCGGGCCCCGGGAGGAACGCGAGGTGGTGGCGGCGGACGTCGGGCGCGCCCCCGCGGGCGGACGCTCCCCCGAGGGGCGCCGGGGCCGGCTGGCGGGTGCCGGGTCTCCCCTCGGCGCCCCGTCCCGCCCCGCCGAGCGGGGCGGGCGGGGGAGGCACCCCCGCGGGGCCTTCGGGTCGTTTCCCTCACCCCAGGGCCAGGTACCTAGCGTCCGCGCCTCCGCGCGTCCGGGGGGCGGGGAGGAAGGAGCGCGGCGCCGGTCCCGAGCGGGCCGCGTCGCCCACACCCCCCTCCTCCCCCCGGGCCGCGGAGCCGGGCGGAGGTTTAAAGACTCGGGCGGCCCGCGGCGCGCGCCGCGAGGTCGGGGGCCGGGGGCGGTCTTCTGCCCGCCGGCGGGACGCCGGGATGGAAGAGAGGAGTCCGGGCGGGGCGCGGCGGCGCGCCCCGCCGGCCCTCTCCCTCCCGAGCCCGCCGGCGGCGTCGGCCGTCGCCGCGCCCTCGGTCCTCCGCGGGGCGGGCCCGGGCCGGAGAGGGGGTCATCCCGTCCCCCCTCTCCGCGGCCTCGGTCTCGGGCGGAGAGCTCGGCGCGCGCGCGGGCGCGCGCTCGCTCCGGCCGGCCTCGCCCGCGTACGGAGCGGGCCGAGACGCGGGTCTCGGCCCGGCGCCCGCCGCTCCCCGCGGCGGTGCGTTCCGCGGCCTCCCCGCCGCGCGCGGCCGGCGGGACGGCGAGCCGGCCGTCCCGCCCGCGCCAGCCGCGGCGCCGGCGGTTCCGCTCCGCCGGTCCGCCCGGCGTGCGTCCGCACGCCCGGCCTCCTGCCCTCCCTCGGGGCCTCGCCGCCGTTTCCCCCTTCCGTCGCAAGCCGCGTCCTCTCCTTCGTCCCCGCCGCCGTCGCCTCCCACCGCGCTTTCGCCCTCGGCCTCGCCGGCCGCGCCGGTCGTGCGAGCGGGAGGTCCGGCGTGGGGCGTCCCGCAGCCGGTCTCCGCGCGGAGGCGCGGGGAGCGGGCGCCGCTCCCGAATCCGTCCCCGTCCCGCCCGCCGCCGTGCGCGCGTCCGCCGCGGGCGCGCCGCCAGGGCGAGCGAGAGGAGGAGGCGTCGGAGGACGAGGGGCGGGGGAGGAAGGTGAGAGGCGGCGGGGGCGTTTCGGTGCGCGCGTCTCCCGCACGGCGAGGAAGGGGCCGAGGTCGGCGCGGGCGCCGTCGGGCGGTCCGGCGCGGGCGCGGGCCGGCGGCGCCGCCGGCGCGGGCGGGGGCCTGGTCTGCTCCCGTCCCCGTCGGTCGCGGCGGCGGCGGCGGCGGTCCGTCGCGGCAGCGGGGCTTCGGCCGGGGCGGCGCGCGCCGTCCCGCGGGCGTCCGCGGCTCCTCCGCCCGGGCCGGGCCGAGCCGGGCGCCTGGTCCGTCCCCGAAGCGAGACAGGGTCGTTTCCCCAGGTCGGGAGCGAGGGCTCCCCGCCCTTCTCGTTCGGGTCGCGCTTCATTGCCGGCCGGCCGGCCGGCCGTCGCCGGCTTTTTTTTTCCCTCCCGCATCCGATATTCGTGTGCTCGTACGGTCAGCGGAGGCGACGCTCGTCCGCCCCGCGGTCGCCCCGGCGTCGGGGCTGGCCGCGGGCGCGGGCCGAGCGCCTTCGGACAAGGCGAGAGAGAACGAGAGCGGTCCCCCGCGCGCGCGGGGCGGTGCCGAAAGTCAGACAACTCTTAGCGGTGGATCACTCGGCTCGTGCGTCGATGAAGAACGCAGCTAGCTGCGAGAATTAATGTGAATTGCAGGACACATTGATCATCGACACTTCGAACGCACTTGCGGCCCCGGGTTCCTCCCGGGGCTACGCCTGCCTGAGCGTCGCTTGACGGTCAATCGCCGACGGCCGCCGTCCGCGGCGGCCGCGCGGCGCGGCTGGGGCGCCTCGCAGGCCCGCGCGCCCCGCCGGAGGCGGGTCGCGGGGGGGGGCCGCCGTCCGTCCGTCCGTCCGCCCGTCGGTCGGTCGGTTCGGGCGCCCGGATTCCCTCCCCCGCACCCCCTCCGAGCGGCGTCGCGCCGCGGGCCTTCGTCCCCCTAAGTGGAGACCCAGGTCGGGGAGCTCGCCGAGCTCCCCGCGCTCCCGGAGCGCCCGCTTTGGCCGAGCTCGTCCCCACGGGGCGGCCGGGCTTTCCGGTCGGTCGCGCGGCGCAGCGCGGCGGGGCCGGACGTTCGTTCGTTCGTTCGTTCGTCCGGCCCCCGCCCCGGAGGAGCGCACCTCGCCCTCCCCGGCCCCGCGCGCGGCTGCCTGCGGGTCGCGTTACCGGCGGCGGTAACGCGCCGTGCTGCCGCGCGCGTGGCGGTCCGGGTCGGGGCGAGGCTGCCGGCCTCCGGTCGTCCGCCCGTCCGTCCGGCCGAGCCCGGCGCGCGTCCCCGCGGGTCCGTCTCCGGCCACCGTGCGCCGGCGGCGGCGGCGGCGGTGCGAACCGCCGGCGGCGCGCCGGCTCCCCCGTCCGGGCGTTCCTCCCTCGGCAGCGCCGGGAGCAGCCGCTTGGCGTCCGAAGGCGGGTGGCCGGGCGAGCGCGGGCTCGCCCGGGGCCCGGCGTTCGGGCCCCGTTTCCGATCGCGACCTCAGGTCAGACGTGGCGACCCGCTGAATTTAAGCATATTAGTCAGCGGAGGAAAAGAAACTAACGAGGATTCCCTCAGTAACGGCGAGTGAAGAGGGAAGAGCCCAGCGCCGAATCCCCGCCCCGCGGTGGGGCGCGGGAGGTGTGGCGTACGGAAGCCCCCATCCCCGGCGCCGCTCTCGGGGGGCCCAAGTCCTTCTGATCGAGGCCCAGCCCGCGGACGGTGTGAGGCCGGTAGCGGCCCCCCGGCGCGCCGGGCCCGGGGCTTCTCGGAGTCGGGTTGCTTGGGAATGCAGCCCAAAGCGGGTGGTAAACTCCATCTAAGGCTAAATACCGGCACGAGACCGATAGCCAACAAGTACCGTAAGGGAAAGTTGAAAAGAACTTTGAAGAGAGAGTTCAAGAGGGCGTGAAACCGTTAAGAGGTAAACGGGTGGGGTCCGCGCAGTCGGCCCGGAGGATTCAACCCGGCGGGCCAAGGTCGGCCGGCGCGGGCGCCGTCGGATCCCCGCCTCCGCCTCCCCTCCGTCCCTCCCCTTCGCCGGGGCGGGGCGGGCCCAGGGGGGCGGGCGGGCCGGGGACCGCCGCCCGGCCGGCGTCCGGCCCCCGTCGGGCGCATTTCCTCCGCGGCGGTGCGCCGCGACCGGCTCCGGGACGGCTGGGAAGGGCTGCCGGCGGGCAGGTGGCCCGGCGCCGCGCGAGCGGCCGCCGGGTGTTATAGCCGCCGGGCCCGGATCGTCGCCGAATCCCGGGGCCGAGGGAGAGGACCGCCGCCGCGCCCTCCCCCGGAGGGGGCGGCCCCCGGAGGGCCCCCCGCGGCCGGACCGGCGTCGGGCCGGCCGCGCCGCGCGCGCGTCCGCGCCGCCGCCGTACGCCGCCGCTCGCTCTCTCTCCGTTCCCCGCCCCGGGTCCGTCCCGGGGCGCGGGGGCGGGGGGGTCGGGTGTCCGGCGCGCGGCTCGGCGCGGCGCCGCGCGTGTGGCGCGCGCCTCCAGCCCGGCGCGGGCGAGGCCGCGGGGGGCGCCGGGGGGGAACCTTCCCCCTTCTGTTCGGGCCGCCTCCGTTCCCGCGGGGGCGGCCCGTTCGGGGGACGGGCCCGCCGGCCCCCGGCGCCGCTGTCCGACCGGGGCGGACTGCGCTCAGTGCGCCCCGACCGCGCGGCGCCGCCGGGCCGGGCTCGGGCCACGCCAGGGCGCCCGGGGTCCGCGGCGACGTCGGCTACCCACCCGACCCGTCTTGAAACACGGACCAAGGAGTCTAGCACGCGCGCGAGTCGGCGGCTCGCGCGAAAGCCCGCGGCGCAATGAAGGTGAGGGCCGGCGCGCGCCGGCTGAGGTGGGATCCCGGGGCGGCAGGCCGGAAGGCCCCGGGCGCACCACCGGCCCGTCTCGCCCGCCTCGCCGGGGAGGTGGAGCATGAGCGCGCGTGCTAGGACCCGAAAGATGGTGAACTATGCCTGGGCAGGGCGAAGCCAGAGGAAACTCTGGTGGAGGTCCGTAGCGGTCCTGACGTGCAAATCGGTCGTCCGACCCGGGTATAGGGGCGAAAGACTAATCGAACCATCTAGTAGCTGGTTCCCTCCGAAGTTTCCCTCAGGATAGCTGGCGCTCGGGGCGGCGGTGCAGTTTTACCCGGTAAAGCGAATGATTAGAGGTCTTGGGGCCGAAACGATCTCAACCTATTCTCAAACTTTCAATGGGTAAGACGCCCGGCTCGCTGGCGTGGAGCCGGGCCGTGGAATGCGAGCGCTCAGTGGGCCACTTTTGGTAAGCAGAACTGGCGCTGCGGGATGAACCGAACGCCGGGTTAAGGCGCCCGATGCCGACGCTCATCAGAGCCCAGAAAAGGTGTTGGTTGATCTAGACAGCAGGACGGTGGCCATGGAAGTCGGAACCCGCTAAGGAGTGTGTAACAACTCACCTGCCGAATCAACTAGCCCTGAAAATGGATGGCGCTGGAGCGTCGGGCCCATACCCGGCCGTCGCCGGCGGTGCGGAGCCGCGGGGGCTACGCCGCGACGAGTAGGAGGGCCGCTGCGGTGCGCCTGGAAGCCTGGGGCGCGGGCCCGGGTGGAGCCGCCGCAGGTGCAGATCTTGGTGGTAGTAGCAACTATTCAAACGAGAGCTTTGAAGGCCGAAGTGGAGCAGGGTTCCATGTGAACAGCAGTTGAACATGGGTCAGTCGGTCCTAAGCGATAGGCGAGCGCCGTTCCGAAGGGACGGGCGATGGCCTCCGTTGCCCTCAGCCGATCGAAAGGGAGTCGGGTTCAGATCCCCGAATCCGGAGCGGCGGAGACGGCGCCGCGAGGCGCCCAGTGCGGTAACGCAAGCGATCCCGGAGAAGCCGGCGGGAGCCCCGGGGAGAGTTCTCTTTTCTTTGTGAAGGGCCGGGCGCCCTGGAACGGGTTCGCCCCGAGAGAGGGGCCCGCGCCTTGGAAAGCGTCGCGGTTCCGGCGGCGTCCGGTGAGCTCTCGCTGGCCCGTGAAAATCCGGGGGAGAGGGTGTAAATCTCGCGCCGGGCCGTACCCATATCCGCAGCAGGTCTCCAAGGTGAACAGCCTCTGGCATGTTGGACCAATGTAGGTAAGGGAAGTCGGCAAGCCGGATCCGTAACTTCGGGATAAGGATTGGCTCTAAGGGCTGGGTCGGTCGGGCTGGGGCGCGAAGCGGGGCTGGGCGCGCGCCGCGGCTGGACGAGGCGCCGCCCGCCCCCGCCCCCCCTTTCCCCGCTCCCGCTCGCCGGGGCGCCGGGGGGGGGGTCAGCGGGCGGCGCGGCGGCGGCGACTCTGGACGCGCGCCGGGCCCTTCCCGTGGATCGCCCCAGCTGCGGCGGGCGCCGCTCGCCCCCCTCCTTGCCCCTCCGCCCCCCCGCTCCCGGCGCCCCTCCCGTCGGCCGTCGTCCCGGCCGCCCCCCGTCCCGAGCGCCCTCCCCGCGAGGGCGCGAGGGGCGGCGGCGGCGGCCGCGGGCGCGGCGGCGGCGGGGGGGGCCCGCCGGCGGCGCCGGGCGGGGCGGTCCCGGGCGGGGGGGGTCTCCGGGCCGGCGCCCCGCCTCGGCCGGCGCCTAGCAGCCGGCTTAGAACTGGTGCGGACCAGGGGAATCCGACTGTTTAATTAAAACAAAGCATCGCGAAGGCCCGCGGCGGGTGTTGACTCTGAATGTCAAAGTGAAGAAATTCAATGAAGCGCGGGTAAACGGCGGGAGTAACTATGACTCTCTTAAGGTAGCCAAATGCCTCGTCATCTAATTAGTGACGCGCATGAATGGATGAACGAGATTCCCACTGTCCCTACCTACTCTCCAGCGAAACCACAGCCAAGGGAACGGGCTTGGCGGAATCAGCGGGGAAAGAAGACCCTGTTGAGCTTGACTCTAGTCTGGCGCTGTGAAGAGACATGAGAGGTGTAGAATAAGTGGGAGGCCCCGCGGTCGCGCGACCCGCGCCGCGGCCCGGCCGCCGGTGAAATACCACTACTCTGATCGTTTTTTCACTTACCCGGTGAGGCGGGGGGGCGAGCCCCGAGGGGCTCTCGCTTCTGGCGCCAAGCGCCCGGCGCGCGCCGGGCGCGACCCGCTCCGGGGACAGCGTCAGGTGGGGAGTTTGACTGGGGCGGTACACCTGTCAAAGCGTAACGCAGGTGTCCTAAGGCGAGCTCAGGGAGGCCAGAAACCTCCCGTGGAGCAGAAGGGCAAAAGCTCGCTTGATCTTGATTTTCAGTACGAATACAGACCGTGAAAGCGGGGCCTCACGATCCTTCTGACTTTTTGGGTTTTAAGCAGGAGGTGTCAGAAAAGTTACCACAGGGATAACTGGCTTGTGGCGGCCAAGCGTTCATAGCGACGTCGCTTTTTGATCCTTCGATGTCGGCTCTTCCTATCATTGTGAAGCAGAATTCACCAAGCGTTGGATTGTTCACCCACTAATAGGGAACGTGAGCTGGGTTTAGACCGTCGTGAGACAGGTTAGTTTTACCCTACTGATGATGTGTTGTTGCGCTAGTAATCCTGCTCAGTACGAGAGGAACCGCAGGTTCAGACATTTGGTGTATGTGCTTGGCTGAGGAGCCACTGGAGCGAGGCTACCATCTGTGGGATTATGACTGAACGCCTCTAAGTCAGAATCCCCCCTAAACGTAGCGATACCGCAGCGCCGAGGCGCCTCGGTGGGCTCGCGATAGCCGGCCGCCGCCCCCCTCGGGCGGGCGGTCGGTGCGGAGCGCCGCTCGTGGTCGGGACCGGAGCGCGGACAGATGTGGCGCCGCCTCTCCCCCGCCGCGTACCGCATGTTCGTGGGGAACCCGGTGCTAAATCATTCGTAGACGACCTGATTCTGGGTCGGGGTTTCGTACGTAGCAGAGCAGCTCCCTCGCTGCGATCTATTGAGAGTCAGCCCTCGACACAAGCTTTTGTCGGAGCGCGGAGCGCGCGCGCGCGCGCGCGTGGCGGCGCCCCGGCGCGGGGCCGGGTCCGGCGGGCCAGTCGGTCGGCTCCCCGCGCCGCTCCGTTTGTTCCTGGGTTCGTTCGTTCGTTCGTTCCTTCCTTCCCCGGCCCCGCGCCGGCGCCGGCGCGGGGTTGGAAAGAGGGGGAGAGGGGCGGGGGGCGCGGCCGGCCCCCTTCCCCGTTTCCGTCCCCGCGGCGCGTGCCGTGGGACGGGCTCCCTCCGTTTTACCCGAGCCCGGGGGTTGACCTGGCGGCCGGGCTAGGGGGCGCTCCGCGTCCCCCTTCGGGGGGTTGACCTGTCGGGCGTTTTTTTTTTATTTTTTTCTCCCTAGGCGGGTCCGGGGGTAGACCTGTCGGCCGGCCGGCCCGGCCCAGCACGCCCCCCCCGTCGGTAAGTGGCTGCGGTGCCGAGGTGGCGGGTAGACCTGGCGGCCGGCAGTACGAACCCCCCCCCCCCCCCCCCCCCCCCCCCACCCCGTTTTTGTTATTTTTGAGGGTTTTTTTCCTTCCTGTTTTTTTTTTTTAATTTTCTTTAAATTGTTTTTTTTCCTTTTCTTTTATTTAATTTTTTCTTTTTTTCTTCATTTTTTAATTAATTTTTTTTTAGTTAGTATTTTAAGAATTCCTTTTTTGTCCCTATTTTCAATTTTTATTTTTTTTCTTGGATTCGTCAGTCGATTTATTTTTTATTTTTATTTTTTTTTTTACATTTTCGGGGATTTAAAAAAAAAATTTTTTTTTTTTATTCATTCGTTCGTGCATTTCTTTCCGTGTGTGTGCGCGCGTGAGTAGGCCCGGCCCGGCCCGGCCCAGCACGCCCCCCCCCCCCGTCGGGAAGTGGCTGCGGTGCCGAGGCAGTGGGTAGACCTGGCGGCCGGCTTTACGACCACCACCCCCCCCCTCGCCGTTTTTGTTCTTATTTTTGACTTCTTTTTAAATTTTTTTTAAATTGACTTTTTTTTAAATTTTTTTTTAAATTGCCTTTTTCTTTTTTTTTTAATTTTTTTTTCATTTTTTAATTAATTATTTTTTTTTAGTTAATATTTTAAGAATTCCTTTTTTTGTCCCTATTTTCAATTTTTAATTTTTTTTATTCATTCGTTCGTGCATTTCTTTCCGTGTGCGTGCGCGCGTGAGTAGGCCCGGCCCGGCCCAGCCCAGCACGCCCCCCCCCCTCTGTCGGGAAGTGGCTGCAGTGCCGAGGTGGCGGGTAGACCTGGCAGACGGAGCTTCAACACCAACCCCCCCCCCCCCAGCACCCCGCCCTGGCAACCTTTTCTGCATTTTTTTTTAATGATTTTTGATTTTTCTTTTCCCGTTTTTTTTTTTTTTAAATTGATTTTTTTTCCCCTATTCCCCCCCCCCCCCTTTTTTTTTTTTTTTTAAAAAAAAAAAAAAGGTGTCCCCCCCTTCCGATGGCTCGGCCGGATAGTGACTCGACTTGGCGTCCGGTGCCCGGCACCCCCCCCCCCAATCCCCTGTATCCGCTCCACCTGATCTGCCCCCCGCTCCTTTTTCTCTTTTTCTCCCCCACATTCGTTCCTTCGTTTGTTCGCTCGCTCTTTATTTCTCTCTCTCTCTCTCATCTCTCTCTCACTCTCTCCATCTTTCTTTCTTTCCTTCTTTGCCTGGGTTCCCCCCCCCCCCCTTACTTTCTTCTTAAATACGTCCGTCCATCCGTGCCATTGGTCGTTTGCTCAGTCGTCGCCCGTTTCTTCCTCGTTCTCGGGGGAAAGTCGGACGGGAGAGGGTCACGGGATTGTTATTTATTTATTCCTCCCTCCCCCCCCCCCCCCCCCCCCTTTCTCTCGCCGTTCATGCCCGCTCCCCTCCTCCCCCCCCACTCGACTCCCGAAGGGCTGGACCCGACGGCTCGGCCGGCCAGTGGCGAGACAGAAGGGTCGGCGCCCCAGCCCCACCCCCCGGATCCGCCCCGCCTGATCGGCCCCCTTTTGTTCGTCTTTCCCCGCCGTTTTTTTGGTTTTGTTTTGTTTTTGTTTTCTGAACGCTGCTTCTCCACACCCCGCCACCCCCCCCCCCGCTTTGATTTTTTTTTCCCGTGCCTTTCTTTCCTCCGGCGGGCACGACCTGATGTCTCGACCGGTCCCCGCGCCGCTCACACCCGCACCCCCCCCCGCGCAACTCCTCCCTGCCCTCCCCCAACCGTGGACCCACCCCCAGCCCGCCACGCGCTTTCTTTCCATTACACTCTTGCTTTCCGTTTTTTTTTTTTTTTTCTACCGGGGGGGGGGGGGTGCGGGGAACGGGCACGTACGTTCGCACTCCCGTAGTCGCTCGCTCTTCTTCCACTTCTCCCTCTAGTAGCGCCCCGCCGGGGGAGCCGAGGGTCCCCTCCCCGAGCGCCGGCGGTCGGACCCGACGGCTCGGACGCCGGCCGGGTGGGAGGCGAGGCGAGGCGAGGCGAGGCGAGGCGAGGCTGCCTCTCCGCCCTCCTGCCGGCCGGGAGAGAGGCTCGCTGCGAGCCCGGTGACCGGACGAGTCGAACAGACGAGTCCCAGGCGGCCCGCGGCCCGCGGCCCGCGGCCCCCGCCGCCGCCGCCGCCGCCGCCGCCGCCGCCGCCGCCGCCGCCACCACCACCACCACCCCCGGGACGGACGACGCCGTCTCCCCCCGGCCTACCTCTGCTACCGTCTTCCCGCCATTATTTTGCGTTCCCGCGCCGACACGAGCGACCGCCCGCATGCGAACCGGCCGTACTTTCCCGCCCGCCCGGCCCCCTTGTGGGCGGAGCCCCCGCCCACACGCCCGCGCCTGCGCACCTCCTCGGGAGGTCGGGGCCGTGTCCGCCGGTCGGGTGGAGGGGGACCGGGGGGAGGGGGGGGGTTACGCGGCGCCCTGGCTAAGGTGTCCCGGCGTTGATGTGGCGCGCGAGCGAGCCGCACGCGAATGGGGTTTCAAAGACCCAGCTTGAAACCGCCTAAAGAGTTACCTAGCCCCCGAGCGAAAAGGAAACGAAACCAAAACGTTCAAACCGGCAAACGAACGGGCACACTGAGGGACAGAGAAATAAACAAAGAAGCAAACACGAGACGAACGCTGCCGTAAGAAGTCGCTGCCGAGCGGCTCGAAAGCAGCGGAACGGTCTACCCGGCCCCAGAGGGCTCCGAGCCGTTCACACGGCGGAGACCGCTAGAGGTCGCTGGCCCGGCCCTCTCCGGGCTGGCAGGCCACGTCTACCCGGCCAGGACGGACTTGGTCGCTCCGCGGCGGCGGCTGGAGGCCTCTGCGGGAGCCGCTCCCGGTCGCCGGACCGGTCGGCCCGGCCGCTCCGTGCACGCACAGGACTCGGTCTCACTGCAGCCGCGGCTGGAGGCCTCTGCAGGACCGGCTCGGGCTCTCCGGAACGGTCGGCCCGGCCCCTGCGTGCCCAAAAAGGACTCGGTCACACAGCAGCAGTGGCTGGAGGCCTCTGCAGGACCGGCTCGGGCCCTCCGGAACGGTCGGCCCGGCCCCTGAGTGCCCCAAAAGGACTCGGTCACACTGCAGGTGCGGCTGGAGGCCGCTGCTGGACCGGCTCCGGCTCTCCGGAATGGTCAGCCCGGCCCCTGTGTGCTCCCACAGGACTCGGTGTCACTGCAGGTGTGGCTGGTGGCCTGTACAGGACCGACTCTGGGTCAGCGGACCGGTCGGCCCGGCCCCTGCGTGCCTCCACAGGACTCGGTCTCACTACAGGTGCGGCTGGAGGCCTGTACAGGCCCGTCTCAGGCTTGCTGGACCGGTCAACCCAACCCCTGCGTGCCCCCACCCGACTCGGCCACCTCCGCAGCTGCGGCTGGAGCATGCTGAAGGAGCCGATCCCGGTCACCGGACCGCTCGCACCGGCCCCTGCGGGCCCGCACAGCACTCGGTCACACTAAAGCCGCAGCTGGAGGCCTCTGCAGGACCGGCTCCGGCTCTCCGGAACGGTCAGCCCGGCCCCTGCGTGCCCAAACAGGACTCGGTCACACAGCAGCCGTGGCTGGAGGCCTCTGCAGGACCGGCTTGGGCTCGCCGTACCGGTCGGCCCAGCCCCTGTGTGCCCCACAGGACTCGGTTTCATTGCAGGTGCGGCTGCAGGCCTCTGCAGGACTGGCTCGGGCCCTCCGGAACGGTCAGCCCGGCACCTGCGTGCCCCCACCTGACTCGGCCACCACCGCAGCTGCGGCTGGAGCATGCTGAAGGAGCCGGTCCCGGTCGCCGGACCGCTCAGACCGGCCCGTGCGGGCCCGCACAGCACTCGGTCACACTAAAGCCGCAGCTGGCTCCCTCTGCACGACCGGCTCGGACACGCCAGACCGGTCAGACCGGCCCCTGGGGACCCCCACAACACTTGGTCACACTAAAGCCGCAGCTGGAGGCCTCTGCAGGACCGGCTCGGGCTTGCCAGACCGGTCGGCCCAGCCCCTGCGTGCCCCACAGGACTCGGTCTCACTGCAGGTGCGGCTGCAGGCCTCTGCAGGACCGGCTCGGGCTCTCCGGAACGGTCAGCCCGGCCCCTGCGTGCCCCCACCCGACTCGGCCTCCTCCGCAGCTGCGGCTGGAGCATGCTGAAGGAGCCGGTCCCGGTCGCCGGACCGCTCGGACCGGCCCCTGCGGGCCCGCACAGCACTCGGTCACACTAAAGCCGCAGCTGGCGCCCTCTGCACGACCGGCTCGGGCTCGCCAGACCGGTCGGACCGGCCCCTGGGGACCCCCACAACACTTGCTCACACTAAAGCCGCAGCTGGAGGCCTCTGCAGGACCGGCTCGGGCTCTCCGGAACGGTCAGCCCGGCCCCTGCGTGCCCAAACAGGACTCGGTCACACAGCAGCCGTGGCTGGAGGCCTCTGCAGGACCGGCTTGGGCTCGCCGTACCGGTCGGCCCAGCCCCTGTGTGCCCCACAGGACTCGGTTTCATTGCAGGTGCGGCTGCAGGCCTCTGCAGGACTGGCTCGGGCCCTCCGGAACGGTCAGCCCGGCCCCTGCGTGCCCCCACCCGACTCGGCCACCTCCGCAGCTGCGGCTGGAGCATGCTGAAGGAGCCGGTCCCGGTCGCCGGACCGGTCGGAACGGCCCCTACGGGCCCGCACAGCACTCGGTCACACTAAAGCTGCAGCTGGCGCCCTCTGCAGGACCGGCTCGGGCTCTCCGGAACGGTCAGCCCGGCCCCTGCGTGCCCAAACAGGACTCGGTCACACAGCAGGTGCGGCTGGTGGCCTGTACAGGACCAGCTCAGGCTCGCCGGACTGGTCACCCGGACCCCTGCGTGCCCCCACCGGACTCGGCCTCCTCCGCAGCTGTGGCTGGAGCATGCTGAAGGAGCCGGTCCGGGTCGCCGGACCGCTCAGACCGGCCCGTGCGGGCCCGCACAGCACTCGGTCACACTAAAGCCGCAGCTGGCTCCCTCTGCACGACCGGCTCGGGCACGCCAGACCGGTCAGACCGGCCCCTGGGGACCCCCACAACACTTGGTCACACTAAAGCCGCAGCTGGAGGCCCCTGCAGGACCGGCTCGGGCTTGCCAGACCGGTCGGCCCTGCCCCTGTGTGCCCCAACAGGACTCGGTCTCACTACAGGTGCGGCTGGAGGCCTGTACAGGACAGTCTCAGGCTTGCTGGACCGGTCAACCCAACCCCTGCGTGCCCCCACCGGACTCGGCCACCTCCGAGGCTGCGGCTGGAGCATGCTAAAGGAGCCGGTCCTGGTCGCCGGACCGCTCAGACCGGCCCGTGCGGGCCCGCACAGCACTCGGTCACACTAAAGCCGCAGCTGGAGGCCTCTGCAGGACCGGCTCGGGCTTGCCAGACCGGTCGGCCCAGCCCCTGCGTGCCCCACAGGACTCGGTTTCATTGCAGGTGCGGCTGCAGGCCTCTGCAGGACCGGCTCGGGCTCTCCGGAACGGTCAGCCCGGCCCCTGCGTGCCCCCACCCGACTCGGCCACCTCCGCAGCTGTGGCTGGAGCATGCTGAAGGAGCCGGTCCCGGTCGCCGGACCGGTCGGAACGGCCCCTGCGGGCCCGCACTGCACTCGGTCACACTAAAGCCGCAGCTGGCGCCCTCTGCAGGACCGGCTCGGGCTCGCCAGACCGGTCGGACCGGCCCCTGGGACCCCCACAACACTTGGTCACACTAAAGCCGCAGCTGGAGGCCTCTGCAGGACCGGCTCGGGCTCTCCGGAACAGTCAGCCCGGCCCCTGCGTGCCCAAACAGGACTCGGTCTCACTGCAGGTGCGGCTGGTGGCCTGTACAGGACCAGCTCAGGCTCGCCGGACCGGTCACCCGGACCCCTGCGTGCCCCCACCGGACTCGGCCTCCTCCGCAGCTGTGGCTGGAGCATGCTGAAGGAGCCGGTCCTGGTCGCCGGACCGCTCAGACCGGCCCGTGCGGGCCCGCACAGCACTCGGTCACACTAAAGCCGCAGCTGGCTCCCTCTGCACGACCGGCTCGGGCACGCCAGACCGGTCGGACCGGCCCCTGGGGACCCCCACAACACTTGGTCACACTAAAGCCGCAGCTGGAGGCCTCTGCAGGACCGGCTCGGGCTCTCCGGAACTGTCGGCCCGGCCCCTGTGTGCCCAACAGGACTCGGTCTCACTGCAGGTGCGGCTGGTGGCCTGTACAGGACCAGCTCAGGCTCGCCGGACTGGTCACCCGGACCCCTGCGTGCCCCCACCGGACTCGGCCTCCTCCGCAGCTGTGGCTGGAGCATGCTGAAGGAGCCGGTCCCGGTCGCCGGACCGCTCAGACCGGCCCGTGCGGGCCTGCACAGCACTCGGTCACACTAAAGCCGCAGCGGGCTCCCTCTGCACGACCGGCTCGGGCTCGCCAGACCGGTCAGACCGGCCCCTGGGGACCCCCACTACACTTGGTCACACTAAAGCCGCAGCTGGAGGCCTGTGCAGGACCGGCTCGGGCTTGCCAGACCGGTCGGCCCAGCCCCTGCGTGCCCCACAGGACTCGGTTTCATTGCAGGTGCGGCTGCAGGCCTCTGCAGGACCGGCTCGGGCTCTCCGGAACGGTCAGCCCAGCCCCTGCGTGCTTCCACCCGACTCGGCCTCCTCCGCAGCTGCGGCTGGAGCATGCTGAAGGAGCCGGTCCCGGTCGCCGGACCGCTCGGACCGGCCCCTGCGGGCCCGCACAGCACTCGGTCACACTAAAGCCGCAGCTGGCGCCCTCTGCAGGACCGGCTCGGGCTCGCCAGACCGGTCGGACCGGCCCCTGGGGACCCCCACAACACTTGGTCACACTAAAGCCGCAGCTGGAGGCCTCTGCAGGACCGGCTCGGGCCCTCCGGAATGGTCGGCCTGGCCCTTGTGTGCCCCACAGGACTCGGTCTCACTGCAGGTGCGGCTGGTGGCCTCTGCAGGACCGGCTCGGGCCCTCCGGAATGGTCGGCCTGGCCCCTGTGTGCCCCACAGGACTCGGTCTCACTGCAGGTGCGGCTGGTGGCCTGTACAGGACCAGCTCAGGCTCGCCGGACTGGTCACCCGGACCCCTGCGTGCCCCCACCGGACTCGGCCTCCTCCGCAGCTGTGGCTGGAGCATGCTGAAGGAGCCGGTCCCGGTCGCCGGACCGCTCAGACCGGCCCGTGCGGGCCCGCACAGCACTCGGTCACACTAAAGCCGCAGCTGGCTCCCTCTGCAGGACCGGCTCGGGCTCGCCAGACCGGTCGGACCGGCCCATGGGGACCCCCACAACACTTGGTCACACTAAAGCCGCAGCTGGAGGCCTCTGCAGGACCGGCTCGGGCTCTCCGGAACTGTCGGCACGGCCCCTGTGTGCCCCACAGGACTCGGTCTCACTGCAGGTGCGGCTGGTGGCCTCTGCAGGACCGGCTCGGGCCCTCCGGAATGGTCGGCCTGGCCCCTGTGTGCCCCACAGGACTCGGTCTCACTGCAGGTGCGGCTGGTGGCCTGTACAGGACCAGCTCAGGCTCGCCGGACTGGTCACCCGGACCCCTGCGTGCCCCCACCGGACTCGGCCTCCTCCGCAGCTGCGGCTGGAGCATGCTGAAGGAGCCGGTCCCGGTCGCCGGACCGCTCAGACCAGCCCGTGCGGGCCCGCACAGCACTCGGTCACACTAAAGCCGCAGCTGGCTCCCTCTGCACGACCGGCTCGGGCACGCCAGACCGGTCAGACCGGCCCCTGGGGACCCCCACAACACTTGGTCACACTAAAGCCGCAGCTGGAGGCCTCTGCAGGACCGGCTCGGGCTTGCCAGACCGGTCAGCCCAGCCCCTGCGTGCCCCACAGGACTCGGTCTCACTGCAGGTGCGGCTGCAGGCCTCTGCAGGACCGGCTCGGGCTCTCCGGAACGGTCAGCCCGGCCCCTGCGTGCCCCCACCCGACTCGGCCTCCTCCGCAGCTGCGGCTGGAGCATGCTGAAGGAGCCGGTCCCGGTCGCCGGACCGCTCGGACCGGCCCCTGCGGGCCCGCACAGCACTCGGTCACACTAAAGCCGCAGCTGGCGCCCTCTGCAGGACCGGCTCGGGCTCGCCAGACCGGTCGGACCGGCCCCTGGGGACCCCCACAACACTTGGTCACACTAAAGCCGCAGCTGGAGGCCTCTGCAGGACCGGCTCGGGCTCTCCGGAACTGTCGGCCCGGCCCCTGTGTGCCCCACAGGACTCGGTCTCACTGCAGGTGCGGCTGGTGGCCTCTGCAGGACCGGCTCGGGCCCTCCGGAATGGTCGGCCTGGCCCCTGTGTGCCCCACAGGACTCGGCCTCCTCCGCAGCTGTGGCTGGAGCATGCTGAAGGAGCCGGTCCCGGTCGCCGGACCGCTCAGACCGGCCCGTGCGGGCCCGCACAGCACTCGGTCACACTAAAGCCGCAGCGGGCTCCCTCTGCACGACCGGCTCGGGCTCGCCAGACCGGTCAGACCGGCCCCTGGGGACCCCCACAACACTTGGTCACACTAAAGCCGCAGCTGGAGGCCTCTACAGGACCGGCTCGGGCTCTCCGGAACTGTCGGCCCGGCCCCTGTGTGCCCCACAGGACTCGGTCTCACTGCAGGTGCGGCTGGTGGCCTGTACAGGACCAGCTCAGGCTCGCCGGACTGGTCACCCGGACCCCTGCGTGCCCCCACCGGACTCGGCCTCCTCCGCAGCTGTGGCTGGAGCATGCTGAAGGAGCCGGTCCCGGTCGCCGGACCGCTCAGACCGGCCCGTGCGGGCCCGCACAGCACTCGGTCACACTAAAGCCGCAGCGGGCTCCCTCTGCACGACCGGCTCGGGCTCGCCAGACCGGTCAGACCGGCCCCTGGGGACCCCCACTACACTTGGTCACACTAAAGCCGCAGCTGGAGGCCTCTGCAGGACCGGCTCGGGCTTGCCAGACCGGTCGGCCCAGCCCCTGCGTGCCCCACAGGACTCGGTTTCATTGCAGGTGCGGCTGCAGGCCTCTGCAGGACCGGCTCGGGCTCTCCGGAACGGTCAGCCCAGCCCCTGCGTGCCCCCACCCGACTCGGCCTCCTCCGCAGCTGCGGCTGGAGCATGCTGAAGGAGCCGGTCCCGGTCGCCGGACCGCTCGGACCGGCCCCTGCGGGCCCGCACAGCACTCGGTCACACTAAAGCCGCAGCTGGCGCCCTCTGCAGGACCGGCTCGGGCTCGCCAGACCGGTCGGACCGGCCCCTGGGGACCCCCACAACACTTGGTCACACTAAAGCCGCAGCTGGAGGCCTCTGCAGGACCGGCTCGGGCTCTCCGGAACTGTCGGCCCGGCCCCTGTGTGCCCCACAGGACTCGGTCTCACTGCAGGTGCGGCTGGTGGCCTCTGCAGGACCGGCTCGGGCTCTCCGGAACGGTCAGCCCGGCCCCTGCGTGCCCCCACCCGACTCGGCCTCCTCCGCAGCTGCGGCTGGAGCATGCTGAAGGAGCCGGTCCCGGTCGCCGGACCGCTCGGACCGGCCCCTGCGGGCCCGCACAGCACTCGGTCACACTAAAGCCGCAGCTGGCGCCCTCTGTAGGACCGGCTCGGGCTCGCCAGACCGGTCGGACCGGCCCCTGGGGACCCCCACAACACTTGCTCACACTAAAGCCGCAGCTGGAGGCCTCTGCAGGACCGGCTCGGGCTCTCCGGAACGGTCAGCCCGGCCCCTGCGTGCCCAAACAGGACTCGGTCACACAGCAGCCGTGGCTGGAGGCCTCTGCAGGACCAGCTTGGGCTCGCCGTACCGGTCGGCCCAGCCCCTGTGTGCCCCACAGGACTCGGTTTCATTGCAGGTGCAGCTGCAGGCCTCTGCAGGACTGTCTCGGGCCCTCCGGAACGGTCAGCCCGGCCCCTGCGTGCCCCCACCCGACTCGGCCACCTCCGCAGCTGCGGCTGGAGCATGCTGAAGGAGCCGGTCCCGGTCGCCGGACCGGTCGGAACGGCCCCTACGGGCCCGCACAGCACTCGGTCACACTAAAGCCGCAGCTGGCGCCCTCTGCAGGACCGGCTCGGGCTCTCCGGAGCGGTCAGCCCGGCCCCTGCGTGCCCAAACAGGACTCGGTCACACAGCAGGTGCGGCTGGTGGCCTGTACAGGACCAGCTCAGGCTCGCCGGACTGGTCACCCGGACCCCTGCGTGCCCCCACCGGACTCGGCCTCCTCCGCAGCTGTGGCTGGAGCATGCTGAAGGAGCCGGTCCTGGTCGCCGGACCGCTCAGACCGGCCCGTGCGGGCCCGCACAGCACTCGGTCACACTAAAGCCGCAGCTGGCTCCCTCTGCACGACCGGCTCGGGCACGCCAGACCGGTCAGACCGGCCCCTGGGGACCCCCACAACACTTGGTCACACTAAAGCCGCAGCTGGAGGCCTCTGCAGGACCGGCTCGGGCTTGCCAGACCGGTCGGCCCAGCCCCTGCGTGCCCCACAGGACTCGGTTTCATTGCAGGTGCGGCTGCAGGCCTCTGCAGGACCGGCTCGGGCTCTCCGGAACGGTCAGCCCAGCCCCTGCGTGCCCCCACCCGACTCGGCCTCCTCCGCAGCTGCGGCTGGAGCATGCTGAAGGAGCCGGTCCCGGTCGCCGGACCGCTCGGACCGGCCCCTGCGGGCCCGCACAGCACTCGGTCACACTAAAGCCGCAGCTGGCGCCCTCTGCAGGACCGGCTCGGGCTCGCCAGACCGGTCGGACCGGCCCCTGGGGACCCCCACAACACTTGGTCACACTAAAGCCGCAGCTGGAGGCCTCTGCAGGACCGGCTCGGGCTCTCCGGAACTGTCGGCCCGGTCCCTGTGTGCCCCACAGGACTCGGTCTCACTGCAGGTGCGGCTGGTGGCCTCTGCAGGACCGGCTCGGGCCCTCCGGAATGGTCGGCCTGGCCCCTGTGTGCCCCACAGGACTCGGTCTCACTGCAGGTGCGGCTGGTGGCCTGTACAGGACCAGCTCAGGCTCGCCGGACTGGTCACCCGGACCCCTGCGTGCCCCCACCGGACTCGGCCTCCTCCGCAGCTGTGGCTGGAGCATGCTGAAGGAGCCGGTCCCGGTCGCCGGACCGCTCAGACCGGCCCGTGCGGGCCCGCACAGCACTCGGTCACACTAAAGCCGCAGCGGGCTCCCTCTGCACGACCGGCTCGGGCTCGCCAGACCGGTCAGACCGGCCCCTGGGGACCCCCACTACACTTGGTCACACTAAAGCCGCAGCTGGAGGCCTCTGCAGGACCGGCTCGGGCTTGCCAGACCGGTCGGCCCAGCCCCTGCGTGCCCCACAGGACTCGGTTTCATTGCAGGTGCGGCTGCAGGCCTCTGCAGGACCGGCTCGGGCTCTCCGGAACGGTCAGCCCGGCCCCTGCGTGCCCCCACCCGACTCGGCCACCTCCGCAGCTGCGGCTGGAGCATGCTGAAGGAGCCGGTCCCGGTCGCCGGACCGCTCAGACCGGCCCGTGCGGGCCCGCACAGCACTCGGTCACACTAAAGCCGCAGCTGGCTCCCTCTGCACGACCGGCTCGGGCTCGCCAGACCGGTCAGACCGGCCCCTGGGGACCCCCACTACACTTGGTCACACTAAAGCCTCAGCTGGAGGCCTCTGCAAGACCGTCTCGGGCTTGCCAGACCGGTCGGCCCGGCCCCTGTGTGCCCCAGCAGGACTCGGTCAAACTGCAGCAGTGGCTGCAGGCCTCTGCAGGACCGGCTCGTGCCCCCCTGAACGGTCAGCCCGACGCCTGCGTGCCCCAACAGGACTCGGTCACACAGCAGTAGTGGCTGGAGGCCTGTGCAGGACCGGCTCGGGCTCGCCGTACCAGTCGGCCCAGCCCCTGTGTGCCCCGCAGGACTTGGTCTCACTGCCGGTTCGGCTGGTGGCCTGTACAGGACCGGCTCAGGCTTTCCTTACCGGTCACCCTGACCCCTGCGTGCCCCCACCCGACTCGGCCACCTCCGCAGCTGTGGCTGGAGCATGCTGAAGGAGTTGGTCCCCATCGCCGGACCGCTCGGACCGGCCCCTGCGGGCCCGCACAGCACTCGGGCACACTAAAACCGCAGCTGGTGCCCTCTGCAGGACCGTCTCGGGCTCGCCAGACCGGTCGGACCGGCCCCTGGGGACCCCCACAACGCTCTGTCACACTAAAGCCGCAGCTGGAGGCCTCTGCAAGACCGGCTCGGGCTTGCCAGACCGGTCAGCCCGGCCTTTGCGTGCCCCAACAGGACTCGGTCAAACTGCAGCAGTGGCTGGAGGCCTCTGCTGGACTGGCTCCGGCTCTCTGGAACGGTCAGCCCGGCCCCTGTGTGCCCCCACAGGACTAGGTCTCACTGCAGGTGCGGCTGCAGGCCTCTGCAGGACCGGCTCGGGCTCGCCGGACCGGTCGGCCCGGCCCCTGCATGCCCCAACAGGACTCGGTCACACTGCAGCCGTGGCTGGAGGCCTACGCAGGACCGGCTCGGACCCTCCGGACCGGTCGGCCCGGCCCTTGCAGGCCCCGAAAAAAATCAGTCTCATGGCAGCCGCAGCTGGAGATCGCTGCCGGGGCGTCTCGTGTACTCCTACACGGTCTACTTGGCCCCGACCGACTCGCACACTCCGCGGCGGCAGCTCGATGTCTCTGCCGGGGCGGCTCGAAAACGGCGGAACGGTCTACCAGGCCCCGGCAGACTTCCTCCGGCTCGGTCGCTCCGCGGCGGCGGCTAGAGGTCGCTGCCGGGGCGGCTGGGTCACGGCGGAACGGTCTACCCGGCCCCGGAGGGTTCCGAGCCGCTTCACCTGTTGTGGCCGCTAGAGGTCGCTGCCGCGTAAATGTCGGGTACGCCGGCCACGTCTACCCGGCCAGGACGGACTTGGTCGCTTCGCGGCGGCGGCTGGAGGTTGCTGCGGGAGCCGCTCCCGGTCGCCGGACCGGTCGGCCCGGCCCCTGCGGGCGCCGAGCGACACGGTCGTTCGGCGGCGGCGGCTGGGTACCGTGGCAGGATCGGCTCGGGTTCGCCGGACCGGTGGTCCCGGCCCGACGGGCACGGGCCACTCGGCCGTTCCGCGGCAATGGGTGGAGACTCGTGTAAGACCGTCTCACGTTCGCCGGATCGGTCGGCCAGGCCCCGTCCGACTCGGTCGCTTCGCGGAGGTAGCTGGAGGTCGCTGCCGTGGCGGCTGGGGCACGGCGGGACGGTCTACCTTGCCCCGGCAGGCTGCGTCCGGCTCGGTCGCTCCGCGGAGGAGGCTAGGGGTCGCTGCCGGGGCGGTTCGGAAACGGCGGGACGGTCTACCCGGCTCCGGCGGTCCCCGTCCGGCTCGGTCTCTCCGCGGCGGCGTCTAGGGGTCGCTGCCGGGGCGTCTCGGAAGCGGCGGGACGGTCTACCGGGCTCCGGCAGGCTTCGTCCGGCTCGGTCGCTCCGCGGCGGCGGCTAGAGGTCGCTGCCGGGGCGGCTGGGTCACGGCGGAACGGTCTACCCCGCCCCGGAGGGTTCCGAGCCGCTTCACCTGTTGTGGCCGCTAGAGGTCGCTGCCGCGTAAATGTCGGGTACGCCGGCCACGTCTACCCGGCCAGGACGGACTTGGTCGCTTCGCGGCGGCGGCTGGAGGTTGCTGCGGGAGCCGCTCCCGGTCGCCGGACCGGTCGGCCCGGCCCCTGCGGGCGCCGAGCGACACGGTCGTTCGGCGGCGGCGGCTGGGGACCGTGGCAGGAGCGGCTCGGGCTCGCCGGACCGGTGGTCCCGGCCCCGTCCGACTCGGTCGCTTCGCGGAGGTGGCTGGAGGTCGCTGCCGTGGCGGCTGGGGCACGGCGGAACGGTCTACCCGGCTCCGGCGGGCTGCGTCCGCCTCGGTCGCTGCCGTGGCGGCTGCTAGGGGTCGCTGCCGGGGTGGCTGGGGCACGGCGGAACGGTCTACCCGGGTCCGGCGGGCCCCGTCCGCCTCGGTCGCTCCGCGGAGGAGGCTAGGGGTCGCTGCCGGGGCGTCTCGGAAACGGCGGGACGGTCTACCTTGCCCCGGCAGGCTTCGTCCGGCTCGGTCGCTCCGCGGCGGCGGCTAGAGGTCGCTGCCGTGTCGGCTCGGAAACGGCGGAACGGTCTACCCGGCCCCGGCAGGCTGCGTCCGGCTCGGTCGCTCCGCGGCGGCGGCTAGAGGTCGCTGCCGGGGCGGCTGGGAAACGGCGGGACGGTCTACCCGGCCCCGGCGGTCCCCGTCCGACTTGGTCGCTCCGCGGCGGCGGCTAGAGGTCGCTGCCGGGGCGTCTCGGAAACGGCGGGACGGTCTACCTTGCCCCGGCAGGCCTCGTCCGGCTCGGTCGCTCCGCGGCGGCGGCTAGGGGTCGCTGCCGTGTCGGCTCGGAAACGGCGGAACGGTCTACCCGGCCCCGGCAGGCTGCGTCCGGCTCGGTCGCTCCGCGGCGGCGGCTAGAGGTCGCTGCCGGGGCGGCTGGGAAACGGCGGGACGGTCTACCCGGCTCCGGCGGGCCCCGTCCGACTTGGTCGCTCCGCGGAGGAGGCTAGGGGTCGCTGCCGGGGCGTCTCGGAAACGGCGGGACGGTCTACCTTGCCCCGGCAGGTTTCGTCCGGCTCGGTCTCTCCGCGGCGGCGGCGGCTAGGGGTCGCTGCCGGGGCGGCTCGGAAACGCCGGCACGGTCTACCTGGCTCCGGCGGTCCCCGTCCGGCTCGGTCGCTCCGCGGCGGCGGCTAGGGGTCGCTGCCGGGGCGGCTGGGGCACGGCGGAACGGTCTACCTGGTCCCGGCGGGCACCGTCCGGCTCGGTCTCTCCGCGGCGGCGGCGGCTAGGGGTCGCTGCCGGGGCGTCTCGGAAACGGCGGAACGGTCTACCCGGGTGCTACCGTCTCGCGCTCTCCGCGGCGGCGGCTAGAGGTCGCTGCCGGGGCGGCTTGCGATCCGCGTCCAGGTCTACCCCGTTTCGGATTGTCTTGGCCGCTCTGGCTGTGGGGGGGGCGCTACAGCTCCGGAGCTGCCAGAGGCGTCGCTGTAATTTTGTACCTCCAGTTACGTCGAGGTAAACCTCGGCTGCCGTCGGAGCCGCTGCCGGTAGTCGGCGCCTATGGGACTAGAACGTTTTTTTCGGATGCCTTATATGTTCGTCTGTAGGAGCGAGTGAGGACTCGGCTCCGGTAGTGGCGGTGAGCGGGCGCTCGCGAGCAGGGTTGACCGGCCGGCCGCCTAGAGAGGGGATCGGCGGCGGCGGCGGCGGCTTTCTCGGGCATCGGTTCGTTCGATCGGTCCGGTCGCTTCGGTTTGTCCGTCGCTCCTCATCCCGCAGCTCTGTCCTGGGCTAAGGCGGTTTTGCAGGCGAGCAGCGAAAAAAAGCCGGAGAAGGCGAGAGAGAGGCAAGAAGCAAGCCGGCTCCCGCGCCGCCAGGGCGAAGGCGAGAGAGAGAGGGAGACGAGAAGGGCACGGGCCGGTCTGCCGGCACCCGAACGTAGGATGGCCGGGGGCGTCCCCGGCGGGTCCCGCCGCGATGGAAGAGGGGGACCCGGAGGTCGTAGGTCGTGGCGGCGTCGCCTCGTCCTCCTTTCGCACCGCATTCTCACCCGCACGCGGGAGCCCCGGCCGATTCGTGGCGCTCCTCGGGCGCGTCGGGGAGGCTTCCCGGCGGGCCGGCTCTATCCCGCTCCCCGGCTCGTTCGGGGTGGCGTGGGGCGGGCCGGTGTTCAGGCACGGGCGAGCACCTCTCGTCGGACGTTGCCCACGCACACCCACCTGCACGTGCGCGTGCGGTCTTTCCGCCGCGCCTGGGGGAAGGGCTCGCGCCTTCTCCCTCCTTTCTTTCTCCTCCCCCCACCCCCTTTCTCCCACCGATCGATGAGGCCACTCGGGTCGCGTCGGAGAGGGCCCCCGGCGGGCCGGCGCTCTGCGCTCCCTGTCCCAGGGAAGCCGCGGCGGCGTCCGGTGTTCAGGCACGGGCGGCCTCCTCTCCAGTTCGCTTCCCGTCGTTCGCGAGGTGAGGCGCTCGCCCGCTTGGGCCGAGGGCGGCGGCGGCGGCGGCTTCGGGGCGCGTGGCCTCGCCGTGCCGACTCGTCTGTCCGCCCGCCCTGTCGGTGCCCCAGGGCTCGCCCGACCGAATCCAGCTGTGTGACGGCCGAGCGGCCCCGCGAGCCGCAGGCGTACCTATTTCGTTGTGAGCGAGGCGTCGGCGCTGCCCTCGTTTCGGGGCCCGGCGAGTGCCGGCCGCGAGCAGCAAGCCGGCGGGGTGGCAACCGAGGGAAACCGCGGGGAACCGAGGCGAAGCGAGCAGCAGCAGAAGAAGGAACGAGAAGACAACGGGGGGCTGCGCCCGGCCGAGCGGGCGAGCCCGGAGCAGCGCGGTGCGTCCCGCTCCGGATCCGTCGGGGTGTGGGGGCCGGGGGCGTCCGCCGGCCTCTCCTCCGCCTTCGGGCCGCCGCAGCCCGTGTCGGTTTTCCTGCCGCGTCCCCGCCCGCTGCGGAGCGTGCCGCCCCGGGAAAGGGTCTCCGATCGTGGGGTCGCGCCCGTCTCGAGGTCGCGTTCTCCTCTAGCACGTCCGTCTCCGGCGGTGGGGCTTCGTTTCCCCGTCCGCTTCTCCGCCGGTCCCGGAGGGCGGGTCAGCCCCGGCCGGCCGTGCGGCGCGAGCGCGAGTCCGGCTCCCGCGGGGGGGCCCGGAGCGTGCCGCCGAAAGCAGCTGCGCAGCGGTCCCCGCTCCTTCCCCGCGGGGGGGAGGTCGGCGGGGCCGCCCCGGGGATCGGGCGCGCCTCTCCGTCGTGGTCGGCGAGCGAGCGAGCGAGCGAGGGAACGACGGAGGGCCGCCCGCCCCGCCGAGAGGCGTTCGCCCCGGCGGCCGCCGCCGTCGACCCGGCAAGGGCCAGACGGGAAAGCCGAGCGAGCAGGCGAGAGAGAGAGAGAGGGAAGGAGCGAGAGCGGTCGGCGGCGGGCCGGGCCCGTCGGGTCGTGCCCCGTGGCGCGGCTACCTGGTTGATCCTGCCAGTAGCATATGCTTGTCTCAAAGATTAAGCCATGCATGTCTAAGTACACACGGGCGGTACAGTGAAACTGCGAATGGCTCATTAAATCAGTTATGGTTCCTTTGGTCGCTCCCCTCCCGTTACTTGGATAACTGTGGTAATTCTAGAGCTAATACATGCCGACGAGCGCCGACCTCCGGGGACGCGTGCATTTATCAGACCAAAACCAACCCGGGCTCGCCCGGCGGCTTTGGTGACTCTAGATAACCTCGAGCCGATCGCACGCCCCCGTGGCGGCGACGACCCATTCGAATGTCTGCCCTATCAACTTTCGATGGTACTGTCTGTGCCTACCATGGTGACCACGGGTAACGGGGAATCAGGGTTCGATTCCGGAGAGGGAGCCTGAGAAACGGCTACCACATCCAAGGAAGGCAGCAGGCGCGCAAATTACCCACTCCCGACCCGGGGAGGTAGTGACGAAAAATAACAATACAGGACTCTTTCGAGGCCCTGTAATTGGAATGAGTCCACTTTAAATCCTTTAACGAGGATCCATTGGAGGGCAAGTCTGGTGCCAGCAGCCGCGGTAATTCCAGCTCCAATAGCGTATATTAAAGTTGCTGCAGTTAAAAAGCTCGTAGTTGGATCTTGGGATCGAGCTGGCGGTCCGCCGCGAGGCGAGCTACCGCCTGTCCCAGCCCCTGTCTCTCGGCGCCCCCTCGATGCTCTTAACTGAGTGTCCCGCGGGGCCCGAAGCGTTTACTTTGAAAAAATTAGAGTGTTCAAAGCAGGCTGGCCGCCGGAATACTCCAGCTAGGAATAATGGAATAGGACTCCGGTTCTATTTTGTTGGTTTTCGGAAACGGGGCCATGATTAAGAGGGACGGCCGGGGGCATTCGTATTGTGCCGCTAGAGGTGAAATTCTTGGACCGGCGCAAGACGAACTAAAGCGAAAGCATTTGCCAAGAATGTTTTCATTAATCAAGAACGAAAGTCGGAGGTTCGAAGACGATCAGATACCGTCGTAGTTCCGACCATAAACGATGCCGACTCGCGATCCGGCGGCGTTATTCCCATGACCCGCCGGGCAGCTCCCGGGAAACCCAAGTCTTTGGGTTCCGGGGGAGTATGGTTGCAAAGCTGAAACTTAAAGGAATTGACGGAAGGGCACCACCAGGAGTGGAGCCTGCGGCTTAATTTGACTCAACACGGGAAACCTCACCCGGCCCGGACACGGACAGGATTGACAGATTGAGAGCTCTTTCTCGATTCCGTGGGTGGTGGTGCATGGCCGTTCTTAGTTGGTGGAGCGATTTGTCTGGTTAATTCCGATAACGAACGAGACTCTGGCATGCTAACTAGTTACGCGACCCCCGAGCGGTCGGCGTCCAACTTCTTAGAGGGACAAGTGGCGTTCAGCCACCCGAGATTGAGCAATAACAGGTCTGTGATGCCCTTAGATGTCCGGGGCTGCACGCGCGCTACACTGACTGGCTCAGCTTGTGTCTACCCTACGCCGGCAGGCGCGGGTAACCCGTTGAACCCCATTCGTGATGGGGATCGGGGATTGCAATTATTCCCCATGAACGAGGAATTCCCAGTAAGTGCGGGTCATAAGCTCGCGTTGATTAAGTCCCTGCCCTTTGTACACACCGCCCGTCGCTACTACCGATTGGATGGTTTAGTGAGGTCCTCGGATCGGCCCCGGCGGGGTCGGCCACGGCCCTGCCGGAGCGTCGAGAAGACGGTCGAACTTGACTATCTAGAGGAAGTAAAAGTCGTAACAAGGTTTCCGTAGGTGAACCTGCGGAAGGATCATTACCGGGGCCGAGGCCGGGCGTCCGGCCGAGCCGTGGCACGAGCGCGCGCGGGCGCGCAGCCTTCCCTTCCCTTCCCCGAGCCCGCTCCGCGCGGAGCGCGGCTCCTCTCCCCCGGTCGAAACGGGGAAAGAAAAAAAAAACACCGCAAGTCGCTCCGCGCGCCTGCCGGCGAGAGAGAAGGGAGACGAGGGCGCGGAGCGCAGCTCCGGGGGGGGAGGCGCGTGTGGGGCGCTCCGGCGCTCCGGCGCGTCTCTCCCCCCCGGCGCCGGTCCGCCGTCGGTCCGCACGCCGCGGGTCCGGTCCGTCCGGTCGCCTCGCCGGCGCGCGCCCGCGCGCGCGCGTCCCGCGGGCCTCGCCCGGGTCGCCGCGCTCCGGAGCGTCCCGCGGCCGAGTCCCGCTCCGACCGCGGGGTCGGGGTCGGGAGGTGGCGGCGGTGCGGAGGGTGGAAGGACGGCTCCCCGCTTCGTCGCTCGGCCGGAAACTCGCCACCGGCCCCCGCCGCTGTCGACGCCGGCACCCCGAGTCCGCTCGGAGGGAAGCCGCGCGGGCGGCCGCGCGCGGGGGAGGCGGCGGGCGGCGGGTCCGAGCGCGGGGCGCGGGAAGTCGGCCGCTTCCCCCGGCCTCACCCCCCACCCCCTTCGCCCGGCCCGTCGCGGGGACGGGGCCGGGTCGCGGGCGGCTGCGGAGCCGGCCGACTCCGGGCGAGCGCCGGAGGGACGCGCGCGCCGCGTACGCGCGGCAGGCGCGAGGTGCCCCGGGCGGCTTCGGTCCCGCGCGGGCGGTCCGAGCCTCGCGGCTCCTCCCGGGTGCAGCTGCCGCCCGGCGCCGGGTTGCCGAGGGAAACCCCGGGCCCCGGGAGGAACGCGAGGTGGTGGCGGCGGACGTCGGGCGCGCCCCCGCGGGCGGACGCTCCCCCGAGGGGCGCCGGGGCCGGCTGGCGGGTGCCGGGTCTCCCCTCGGCGCCCCGTCCCGCCCCGCCGAGCGGGGCGGGCGGGGGAGGCACCCCCGCGGGGCCTTCGGGTCGTTTCCCTCACCCCAGGGCCAGGTACCTAGCGTCCGCGCCTCCGCGCGTCCGGGGGGCGGGGAGGAAGGAGCGCGGCGCCGGTCCCGAGCGGGCCGCGTCGCCCACACCCCCCTCCTCCCCCCGGGCCGCGGAGCCGGGCGGAGGTTTAAAGACTCGGGCGGCCCGCGGCGCGCGCCGCGAGGTCGGGGGCCGGGGGCGGTCTTCTGCCCGCCGGCGGGACGCCGGGATGGAAGAGAGGAGTCCGGGCGGGGCGCGGCGGCGCGCCCCGCCGGCCCTCTCCCTCCCGAGCCCGCCGGCGGCGTCGGCCGTCGCCGCGCCCTCGGTCCTCCGCGGGGCGGGCCCGGGCCGGAGAGGGGGTCATCCCGTCCCCCCTCTCCGCGGCCTCGGTCTCGGGCGGAGAGCTCGGCGCGCGCGCGGGCGCGCGCTCGCTCCGGCCGGCCTCGCCCGCGTACGGAGCGGGCCGAGACGCGGGTCTCGGCCCGGCGCCCGCCGCTCCCCGCGGCGGTGCGTTCCGCGGCCTCCCCGCCGCGCGCGGCCGGCGGGACGGCGAGCCGGCCGTCCCGCCCGCGCCAGCCGCGGCGCCGGCGGTTCCGCTCCGCCGGTCCGCCCGGCGTGCGTCCGCACGCCCGGCCTCCTGCCCTCCCTCGGGGCCTCGCCGCCGTTTCCCCCTTCCGTCGCAAGCCGCGTCCTCTCCTTCGTCCCCGCCGCCGTCGCCTCCCACCGCGCTTTCGCCCTCGGCCTCGCCGGCCGCGCCGGTCGTGCGAGCGGGAGGTCCGGCGTGGGGCGTCCCGCAGCCGGTCTCCGCGCGGAGGCGCGGGGAGCGGGCGCCGCTCCCGAATCCGTCCCCGTCCCGCCCGCCGCCGTGCGCGCGTCCGCCGCGGGCGCGCCGCCAGGGCGAGCGAGAGGAGGAGGCGTCGGAGGACGAGGGGCGGGGGAGGAAGGTGAGAGGCGGCGGGGGCGTTTCGGTGCGCGCGTCTCCCGCACGGCGAGGAAGGGGCCGAGGTCGGCGCGGGCGCCGTCGGGCGGTCCGGCGCGGGCGCGGGCCGGCGGCGCCGCCGGCGCGGGCGGGGGCCTGGTCTGCTCCCGTCCCCGTCGGTCGCGGCGGCGGCGGCGGCGGCGGTCCGTCGCGGCAGCGGGGCTTCGGCCGGGGCGGCGCGCGCCGTCCCGCGGGCGTCCGCGGCTCCTCCGCCCGGGCCGGGCCGAGCCGGGCGCCTGGTCCGTCCCCGAAGCGAGACAGGGTCGTTTCCCCAGGTCGGGAGCGAGGGCTCCCCGCCCTTCTCGTTCGGGTCGCGCTTCATTGCCGGCCGGCCGGCCGTCGCCGGCTTTTTTTTTCCCTCCCGCATCCGATATTCGTGTGCTCGTACGGTCAGCGGAGGCGACGCTCGTCCGCCCCGCGGTCGCCCCGGCGTCGGGGCTGGCCGCGGGCGCGGGCCGAGCGCCTTCGGGCAAGGCGAGAGAGAACGAGAGCGGTCCCCCCGCGCGCGCGGGGCGGTGCCGAAAGTCAGACAACTCTTAGCGGTGGATCACTCGGCTCGTGCGTCGATGAAGAACGCAGCTAGCTGCGAGAATTAATGTGAATTGCAGGACACATTGATCATCGACACTTCGAACGCACTTGCGGCCCCGGGTTCCTCCCGGGGCTACGCCTGCCTGAGCGTCGCTTGACGGTCAATCGCCGACGGCCGCCGTCCGCGGCGGCCGCGCGGCGCGGCTGGGGCGCCTCGCAGGCCCGCGCGCCCCGCCGGAGGCGGGTCGCGAGGGGGGGGGCCGCCGTCCGTCCGTCCGTCCGCCCGTCGGTCGGTCGGTTCGGGCGCCCGGATTCCCTCCCCCGCACCCCCTCCGAGCGGCGTCGCGCCGCGGGCCTTCGTCCCCCTAAGTGGAGACCCAGGTCGGGGAGCTCGCCGAGCTCCCCGCGCTCCCGGAGCGCCCGCTTTGGCCGAGCTCGTCCCCACGGGGCGGCCGGGCTTTCCGGTCGGTCGCGCGGCGCAGCGCGGCGGGGCCGGACGTTCGTTCGTTCGTTCGTTCGTCCGGCCCCCCGCCCCGGAGGAGCGCACCTCGCCCTCCCCGGCCCCGCGCGCGGCTGCCTGCGGGTCGCGTTACCGGCGGCGGTAACGCGCCGTGCTGCCGCGCGCGTGGCGGTCCGGGTCGGGGCGAGGCTGCCGGCCTCCGGTCGTCCGCCCGTCCGTCCGGCCGAGCCCGGCGCGCGTCCCCGCGGGTCCGTCTCCGGCCACCGTGCGCCGGCGGCGGCGGCGGCGGCGGTGCGAACCGCCGGCGGCGCGCCGGCTCCCCCGTCCGGGCGTTCCTCCCTCGGCAGCGCCGGGAGCAGCCGCTTGGCGTCCGAAGGCGGGTGGCCGGGCGAGCGCGGGCTCGCCCGGGGCCCGGCGTTCGGGCCCCGTTTCCGATCGCGACCTCAGGTCAGACGTGGCGACCCGCTGAATTTAAGCATATTAGTCAGCGGAGGAAAAGAAACTAACGAGGATTCCCTCAGTAACGGCGAGTGAAGAGGGAAGAGCCCAGCGCCGAATCCCCGCCCCGCGGTGGGGCGCGGGAGGTGTGGCGTACGGAAGCCCCCATCCCCGGCGCCGCTCTCGGGGGGCCCAAGTCCTTCTGATCGAGGCCCAGCCCGCGGACGGTGTGAGGCCGGTAGCGGCCCCCCGGCGCGCCGGGCCCGGGGCTTCTCGGAGTCGGGTTGCTTGGGAATGCAGCCCAAAGCGGGTGGTAAACTCCATCTAAGGCTAAATACCGGCACGAGACCGATAGCCAACAAGTACCGTAAGGGAAAGTTGAAAAGAACTTTGAAGAGAGAGTTCAAGAGGGCGTGAAACCGTTAAGAGGTAAACGGGTGGGGTCCGCGCAGTCGGCCCGGAGGATTCAACCCGGCGGGCCAAGGTCGGCCGGCGCGGGCGCCGTCGGATCCCCGCCTCCGCCTCCCCTCCGTCCCTCCCCTTCGCCGGGGCGGGGCGGGCCCAGGGGGGGCGGGCGGGCCGGGGACCGCCGCCCGGCCGGCGTCCGGCCCCCGTCGGGCGCATTTCCTCCGCGGCGGTGCGCCGCGACCGGCTCCGGGACGGCTGGGAAGGGCTGCCGGCGGGCAGGTGGCCCGGCGCCGCGCGAGCGGCCGCCGGGTGTTATAGCCGCCGGGCCCGGATCGTCGCCGAATCCCGGGGCCGAGGGAGAGGACCGCCGCCGCGCCCTCCCCCGGAGGGGGCGGCCCCCCGGAGGGCCCCCCGCGGCCGGACCGGCGTCGGGCCGGCCGCGCCGCGCGCGCGTCCGCGCCGCCGCCGTACGCCGCCGCTCGCTCTCTCTCCGTTCCCCGCCCCGGGTCCGTCCCGGGGCGCGGGGCGGGGGGGGTCGGGTGTCCGGCGCGCGGCTCGGCGCGGCGCCGCGCGTGTGGCGCGCGCCTCCAGCCCGGCGCGGGCGAGGCCGCGGGGGGCGCCGGGGGGGAACCTTCCCCCTTCTGTTCGGGCCGCCTCCGTTCCCGCGGGGGCGGCCCGTTCGGGGGACGGGCCCGCCGGCCCCCGGCGCCGCTGTCCGACCGGGGCGGACTGCGCTCAGTGCGCCCCGACCGCGCGGCGCCGCCGGGCCGGGCTCGGGCCACGCCAGGGCGCCCGGGGTCCGCGGCGACGTCGGCTACCCACCCGACCCGTCTTGAAACACGGACCAAGGAGTCTAGCACGCGCGCGAGTCGGCGGCTCGCGCGAAAGCCCGCGGCGCAATGAAGGTGAGGGCCGGCGCGCGCCGGCTGAGGTGGGATCCCGGGGCGGCAGGCCGGAAGGCCCCGGGCGCACCACCGGCCCGTCTCGCCCGCCTCGCCGGGGAGGTGGAGCATGAGCGCGCGTGCTAGGACCCGAAAGATGGTGAACTATGCCTGGGCAGGGCGAAGCCAGAGGAAACTCTGGTGGAGGTCCGTAGCGGTCCTGACGTGCAAATCGGTCGTCCGACCCGGGTATAGGGGCGAAAGACTAATCGAACCATCTAGTAGCTGGTTCCCTCCGAAGTTTCCCTCAGGATAGCTGGCGCTCGGGGCGGCGGTGCAGTTTTACCCGGTAAAGCGAATGATTAGAGGTCTTGGGGCCGAAACGATCTCAACCTATTCTCAAACTTTCAATGGGTAAGACGCCCGGCTCGCTGGCGTGGAGCCGGGCCGTGGAATGCGAGCGCTCAGTGGGCCACTTTTGGTAAGCAGAACTGGCGCTGCGGGATGAACCGAACGCCGGGTTAAGGCGCCCGATGCCGACGCTCATCAGAGCCCAGAAAAGGTGTTGGTTGATCTAGACAGCAGGACGGTGGCCATGGAAGTCGGAACCCGCTAAGGAGTGTGTAACAACTCACCTGCCGAATCAACTAGCCCTGAAAATGGATGGCGCTGGAGCGTCGGGCCCATACCCGGCCGTCGCCGGCGGTGCGGAGCCGCGGGGGCTACGCCGCGACGAGTAGGAGGGCCGCTGCGGTGCGCCTGGAAGCCTGGGGCGCGGGCCCGGGTGGAGCCGCCGCAGGTGCAGATCTTGGTGGTAGTAGCAACTATTCAAACGAGAGCTTTGAAGGCCGAAGTGGAGCAGGGTTCCATGTGAACAGCAGTTGAACATGGGTCAGTCGGTCCTAAGCGATAGGCGAGCGCCGTTCCGAAGGGACGGGCGATGGCCTCCGTTGCCCTCAGCCGATCGAAAGGGAGTCGGGTTCAGATCCCCGAATCCGGAGCGGCGGAGACGGGCGCCGCGAGGCGCCCAGTGCGGTAACGCAAGCGATCCCGGAGAAGCCGGCGGGAGCCCCGGGGAGAGTTCTCTTTTCTTTGTGAAGGGCCGGGCGCCCTGGAACGGGTTCGCCCCGAGAGAGGGGCCCGCGCCTTGGAAAGCGTCGCGGTTCCGGCGGCGTCCGGTGAGCTCTCGCTGGCCCGTGAAAATCCGGGGGAGAGGGTGTAAATCTCGCGCCGGGCCGTACCCATATCCGCAGCAGGTCTCCAAGGTGAACAGCCTCTGGCATGTTGGACCAATGTAGGTAAGGGAAGTCGGCAAGCCGGATCCGTAACTTCGGGATAAGGATTGGCTCTAAGGGCTGGGTCGGTCGGGCTGGGGCGCGAAGCGGGGCTGGGCGCGCGCCGCGGCTGGACGAGGCGCCGCCCGCCCCCGCCCCCCCTTTCCCCGCTCCCGCTCGCCGGGGCGCCGGGGGGGGGGTCAGCGGGCGGCGCGGCGGCGGCGACTCTGGACGCGCGCCGGGCCCTTCCCGTGGATCGCCCCAGCTGCGGCGGGCGCCGCTCGCCCCCCTCCTTGCCCCTCCGCCCCCCCGCTCCCGGCGCCCCTCCCGTCGGCCGTCGTCCCGGCCGCCCCCCGTCCCGAGCGCCCTCCCCGCGAGGGCGCGAGGGGCGGCGGCGGCGGCCGCGGGCGCGGCGGCGGCGGGGGGGGCCCGCCGGCGGCGCCGGGCGGGGCGGTCCCGGGCGGGGGGGGTCTCCGGGCCGGCGCCCCGCCTCGGCCGGCGCCTAGCAGCCGGCTTAGAACTGGTGCGGACCAGGGGAATCCGACTGTTTAATTAAAACAAAGCATCGCGAAGGCCCGCGGCGGGTGTTGACGCGATGTGATTTCTGCCCAGTGCTCTGAATGTCAAAGTGAAGAAATTCAATGAAGCGCGGGTAAACGGCGGGAGTAACTATGACTCTCTTAAGGTAGCCAAATGCCTCGTCATCTAATTAGTGACGCGCATGAATGGATGAACGAGATTCCCACTGTCCCTACCTACTCTCCAGCGAAACCACAGCCAAGGGAACGGGCTTGGCGGAATCAGCGGGGAAAGAAGACCCTGTTGAGCTTGACTCTAGTCTGGCGCTGTGAAGAGACATGAGAGGTGTAGAATAAGTGGGAGGCCCCGCGGTCGCGCGACCCGCGCCGCGGCCCGGCCGCCGGTGAAATACCACTACTCTGATCGTTTTTTCACTTACCCGGTGAGGCGGGGGGGCGAGCCCCGAGGGGCTCTCGCTTCTGGCGCCAAGCGCCCGGCGCGCGCCGGGCGCGACCCGCTCCGGGGACAGCGTCAGGTGGGGAGTTTGACTGGGGCGGTACACCTGTCAAAGCGTAACGCAGGTGTCCTAAGGCGAGCTCAGGGAGGCCAGAAACCTCCCGTGGAGCAGAAGGGCAAAAGCTCGCTTGATCTTGATTTTCAGTACGAATACAGACCGTGAAAGCGGGGCCTCACGATCCTTCTGACTTTTTGGGTTTTAAGCAGGAGGTGTCAGAAAAGTTACCACAGGGATAACTGGCTTGTGGCGGCCAAGCGTTCATAGCGACGTCGCTTTTTGATCCTTCGATGTCGGCTCTTCCTATCATTGTGAAGCAGAATTCACCAAGCGTTGGATTGTTCACCCACTAATAGGGAACGTGAGCTGGGTTTAGACCGTCGTGAGACAGGTTAGTTTTACCCTACTGATGATGTGTTGTTGCGCTAGTAATCCTGCTCAGTACGAGAGGAACCGCAGGTTCAGACATTTGGTGTATGTGCTTGGCTGAGGAGCCACTGGAGCGAGGCTACCATCTGTGGGATTATGACTGAACGCCTCTAAGTCAGAATCCCCCCTAAACGTAGCGATACCGCAGCGCCGAGGCGCCTCGGTGGGCTCGCGATAGCCGGCCGCCGCCCCCCTCGGGCGGGCGGTCGGTGCGGAGCGCCGCTCGTGGTCGGGACCGGAGCGCGGACAGATGTGGCGCCGCCTCTCCCCCGCCGCGTACCGCATGTTCGTGGGGAACCCGGTGCTAAATCATTCGTAGACGACCTGATTCTGGGTCGGGGTTTCGTACGTAGCAGAGCAGCTCCCTCGCTGCGATCTATTGAGAGTCAGCCCTCGACACAAGCTTTTGTCGGAGCGCGGAGCGCGCGCGCGCGCGCGCGTGGCGGCGCCCCGGCGCGGGGCCGGGTCCGGCGGGCCAGTCGGTCGGCTCCCCGCGCCGCTCCGTTTGTTCCTGGGTTCGTTCGTTCGTTCGTTCCTTCCTTCCCCGGCCCCGCGCCGGCGCCGGCGCGGGGTTGGAAAGAGGGGGAGAGGGGCGGGGGGCGCGGCCGGCCCCCTTCCCCGTTTCCGTCCCCGCGGCGCGTGCCGTGGGACGGGCTCCCTCCGTTTTACCCGAGCCCGGGGGTTGACCTGGCGGCCGGGCTAGGGGGCGCTCCGCGTCCCCCTTCGGGGGGTTGACCTGTCGGGCGTTTTTTTTTTCTCCCTAGGCGGGTCCGGGGGTAGACCTGTCGGCCGGCCGGCCCGGCCCAGCACGCCCCCCCCGTCGGTAAGTGGCTGCGGTGCCGAGGTGGCGGGTAGACCTGGCGGCCGGCAGTACGAACCCCCCCCCCCCCCCCCCCCCCACCCCGTTTTTGTTATTTTTGAGGGTTTTTTTCCTTCCTGTTTTTTTTTTTTAATTTTCTTTAAATTGTTTTTTTTCCTTTTCTTTTATTTAATTTTTTCTTTTTTTCTTCATTTTTTAATTAATTTTTTTTTAGTTAGTATTTTAAGAATTCCTTTTTTGTCCCTATTTTCAATTTTTATTTTTTTTCTTGGATTCGTCAGTCGATTTATTTTTTATTTTTATTTTTTTTTTTTACATTTTCGGGGTTTTGATTTAAAAAAAAAAATTATTTTTTTTTTATTCATTCGTTCGTGCATTTCTTTCCGTGTGCGTGTGCGCGTGAGTAGGCCCGGCCCGGCCCGGCCCGGCCCGGCCCGGCCCAGCACGCCCCCCCGTCGGTAAGTGGCTGCGGTGCCGAGGCAGTGGGTAGACCTGGCGGCCGGCTTTACGACCACCACCCCCCCCTCGCCATTTTTGTTCTTATTTTTGACTTTTTAAAAAAAAAAAAATTAAATTGACTTTAATTTTTTTTTAAATTGCCTTTTCCTTTTTTTTTTTAATTTTTTTTTCATTTTTTAATTAATTTTTTTTTTTTAGTTAGTATTTTAAGAATTCCTTTTTTGTCCCTATTTTCAATTTTTAATTTTTTTCTTGGATTCGTCAGTCGATTTATTTTTTATTATTTTTATATTTTTTTTACATTTTCGGTTTTTTATTTTTTTTTAATTTTTTTAATTTTTTTTTTTATTCATTCGTTCGTGCATTTCTTTCCGTGTGTGTGCGCGCGTGAGTAGGCCCGGCCCGGCCCGGCCCAGCACGCCCCCCCCCCCCGTCGGGAAGTGGCTGCGGTGCCGAGGCAGTGGGTAGACCTGGCGGCCGGCTTTACGACCACCACCCCCCCCCTCGCCGTTTTTGTTCTTATTTTTGACTTCTTTTTAAATTTTTTTTAAATTGACTTTTTTTTAAATTTTTTTTTAAATTGCCTTTTTCTTTTTTTTTTAATTTTTTTTTCATTTTTTAATTAATTATTTTTTTTTAGTTAATATTTTAAGAATTCCTTTTTTTGTCCCTATTTTCAATTTTTAATTTTTTTTATTCATTCGTTCGTGCATTTCTTTCCGTGTGCGTGCGCGCGTGAGTAGGCCCGGCCCGGCCCAGCCCAGCACGCCCCCCCCCTCTGTCGGGAAGTGGCTGCAGTGCCGAGGTGGCGGGTAGACCTGGCAGACGGAGCTTCAACACCAACCCCCCCCCCCCCAGCACCCCGCCCTGGCAACCTTTTCTGCATTTTTTTTTAATGATTTTTGATTTTTCTTTTCCCGTTTTTTTTTTTTTTAAATTGATTTTTTTTCCCCTATTCCCCCCCCCCCCCCTTTTTTTTTTTTTTTTAAAAAAAAAAAAAGGTGTCCCCCCCTTCCGATGGCTCGGCCGGATAGTGACTCGACTTGGCGTCCGGTGCCCGGCACCCCCCCCCCCAATCCCCTGTATCCGCTCCACCTGATCTGCCCCCCGCTCCTTTTTCTCTTTTTCTCCCCCACATTCGTTCCTTCGTTTGTTCGCTCGCTCTTTATTTCTCTCTCTCTCTCTCATCTCTCTCTCACTCTCTCCATCTTTCTTTCTTTCCTTCTTTGCCTGGGTTCCCCCCCCCCCCCTTACTTTCTTCTTAAATACGTCCGTCCATCCGTGCCATTGGTCGTTTGCTCAGTCGTCGCCCGTTTCTTCCTCGTTCTCGGGGGAAAGTCGGACGGGAGAGGGTCACGGGATTGTTATTTATTTATTCCTCCCTCCCCCCCCCCCCCCCCCCCTTTCTCTCGCCGTTCATGCCCGCTCCCCTCCTCCCCCCCCACTCGACTCCCGAAGGGCTGGACCCGACGGCTCGGCCGGCCAGTGGCGAGACAGAAGGGTCGGCGCCCCAGCCCCACCCCCCGGATCCGCCCCGCCTGATCGGCCCCCTTTTGTTCGTCTTTCCCCGCCGTTTTTTTGGTTTTGTTTTGTTTTTGTTTTCTGAACGCTGCTTCTCCACACCCCGCCACCCCCCCCCCCGCTTTGATTTTTTTTTCCCGTGCCTTTCTTTCCTCCGGCGGGCACGACCTGATGTCTCGACCGGTCCCCGCGCCGCTCACACCCGCACCCCCCCCCCGCGCAACTCCTCCCTGCCCTCCCCCAACCGTGGACCCACCCCCAGCCCGCCACGCGCTTTCTTTCCATTACACTCTTGCTTTCCGTTTTTTTTTTTTTTTTCTACCGGGGGGGGGGGGGTGCGGGGAACGGGCACGTACGTTCGCACTCCCGTAGTCGCTCGCTCTTCTTCCACTTCTCCCTCTAGTAGCGCCCCGCCGGGGGAGCCGAGGGTCCCCTCCCCGAGCGCCGGCGGTCGGACCCGACGGCTCGGACGCCGGCCGGGTGGGAGGCGAGGCGAGGCGAGGCGAGGCGAGGCGAGGCTGCCTCTCCGCCCTCCTGCCGGCCGGGAGAGAGGCTCGCTGCGAGCCCGGTGACCGGACGAGTCGAACAGACGAGTCCCAGGCGGCCCGCGGCCCGCGGCCCCGCCCCCCGCCGCCGCCGCCGCCGCCGCCGCCGCCGCCGCCGCCGCCGCCACCACCACCACCACCCCCGGGACGGACGACGCCGTCTCCCCCCGGCCTACCTCTGCTACCGTCTTCCCGCCATTATTTTGCGTTCCCGCGCCGACACGAGCGACCGCCCGCATGCGAACCGGCCGTACTTTCCCGCCCGCCCGGCCCCCTTGTGGGCGGAGCCCCCGCCCACACGCCCGCGCCTGCGCACCTCCTCGGGAGGTCGGGGCCGTGTCCGCCGGTCGGGTGGAGGGGGACCGGGGGGAGGGGGGGGGGTTACGCGGCGCCCTGGCTAAGGTGTCCCGGCGTTGATGTGGCGCGCGAGCGAGCCGCACGCGAATGGGGTTTCAAAGACCCAGCTTGAAACCGCCTAAAGAGTTACCTAGCCCCCGAGCGAAAAGGAAACGAAACCAAAACGTTCAAACCGGCAAACGAACGGGCACACTGAGGGACAGAGAAATAAACAAAGAAGCAAACACGAGACGAACGCTGCCGTAAGAAGTCGCTGCCGAGCGGCTCGAAAGCAGCGGAACGGTCTACCCGGCCCCAGAGGGCTCCGAGCCGTTCACACGGCGGAGACCGCTAGAGGTCGCTGGCCCGGCCCTCTCCGGGCTGGCAGGCCACGTCTACCCGGCCAGGACGGACTTGGTCGCTCCGCGGCGGCGGCTGGAGGCCTCTGCGGGAGCCGCTCCCGGTCGCCGGACCGGTCGGCCCGGCCGCTCCGTGCACGCACAGGACTCGGTCTCACTGCAGCCGCGGCTGGAGGCCTCTGCAGGACCGGCTCGGGCTCTCCGGAACGGTCGGCCCGGCCCCTGCGTGCCCAAAAAGGACTCGGTCACACAGCAGCAGTGGCTGGAGGCCTCTGCAGGACCGGCTCGGGCCCTCCGGAACGGTCGGCCCGGCCCCTGAGTGCCCCAAAAGGACTCGGTCACACTGCAGGTGCGGCTGGAGGCCGCTGCTGGACCGGCTCCGGCTCTCCGGAATGGTCAGCCCGGCCCCTGTGTGCTCCCACAGGACTCGGTGTCACTGCAGGTGTGGCTGGTGGCCTGTACAGGACCGACTCTGGGTCAGCGGACCGGTCGGCCCGGCCCCTGCGTGCCTCCACAGGACTCGGTCTCACTACAGGTGCGGCTGGAGGCCTGTACAGGCCCGTCTCAGGCTTGCTGGACCGGTCAACCCAACCCCTGCGTGCCCCCACCCGACTCGGCCACCTCCGCAGCTGCGGCTGGAGCATGCTGAAGGAGCCGATCCCGGTCACCGGACCGCTCGCACCGGCCCCTGCGGGCCCGCACAGCACTCGGTCACACTAAAGCCGCAGCTGGAGGCCTCTGCAGGACCGGCTCCGGCTCTCCGGAACGGTCAGCCCGGCCCCTGCGTGCCCAAACAGGACTCGGTCACACAGCAGCCGTGGCTGGAGGCCTCTGCAGGACCGGCTTGGGCTCGCCGTACCGGTCGGCCCAGCCCCTGTGTGCCCCACAGGACTCGGTTTCATTGCAGGTGCGGCTGCAGGCCTCTGCAGGACTGGCTCGGGCCCTCCGGAACGGTCAGCCCGGCACCTGCGTGCCCCCACCTGACTCGGCCACCACCGCAGCTGCGGCTGGAGCATGCTGAAGGAGCCGGTCCCGGTCGCCGGACCGCTCAGACCGGCCCGTGCGGGCCCGCACAGCACTCGGTCACACTAAAGCCGCAGCTGGCTCCCTCTGCACGACCGGCTCGGACACGCCAGACCGGTCAGACCGGCCCCTGGGGACCCCCACAACACTTGGTCACACTAAAGCCGCAGCTGGAGGCCTCTGCAGGACCGGCTCGGGCTTGCCAGACCGGTCGGCCCAGCCCCTGCGTGCCCCACAGGACTCGGTCTCACTGCAGGTGCGGCTGCAGGCCTCTGCAGGACCGGCTCGGGCTCTCCGGAACGGTCAGCCCGGCCCCTGCGTGCCCCCACCCGACTCGGCCTCCTCCGCAGCTGCGGCTGGAGCATGCTGAAGGAGCCGGTCCCGGTCGCCGGACCGCTCGGACCGGCCCCTGCGGGCCCGCACAGCACTCGGTCACACTAAAGCCGCAGCTGGCGCCCTCTGCACGACCGGCTCGGGCTCGCCAGACCGGTCGGACCGGCCCCTGGGGACCCCCACAACACTTGCTCACACTAAAGCCGCAGCTGGAGGCCTCTGCAGGACCGGCTCGGGCTCTCCGGAACGGTCAGCCCGGCCCCTGCGTGCCCAAACAGGACTCGGTCACACAGCAGCCGTGGCTGGAGGCCTCTGCAGGACCGGCTTGGGCTCGCCGTACCGGTCGGCCCAGCCCCTGTGTGCCCCACAGGACTCGGTTTCATTGCAGGTGCAGCTGCAGGCCTCTGCAGGACTGGCTCGGGCCCTCCGGAACGGTCAGCCCGGCCCCTGCGTGCCCCCACCCGACTCGGCCACCTCCGCAGCTGCGGCTGGAGCATGCTGAAGGAGCCGGTCCCGGTCGCCGGACCGGTCGGAACGGCCCCTACGGGCCCGCACAGCACTCGGTCACACTAAAGCCGCAGCTGGCGCCCTCTGCAGGACCGGCTCGGGCTCTCCGGAACGGTCAGCCCGGCCCCTGCGTGCCCAAACAGGACTCGGTCACACAGCAGGTGCGGCTGGTGGCCTGTACAGGACCAGCTCAGGCTCGCCGGACTGGTCACCCGGACCCCTGCGTGCCCCCACCGGACTCGGCCTCCTCCGCAGCTGTGGCTGGAGCATGCTGAAGGAGCCGGTCCGGGTCGCCGGACCGCTCAGACCGGCCCGTGCGGGCCCGCACAGCACTCGGTCACACTAAAGCCGCAGCTGGCTCCCTCTGCACGACCGGCTCGGGCACGCCAGACCGGTCAGACCGGCCCCTGGGGACCCCCACAACACTTGGTCACACTAAAGCCGCAGCTGGAGGCCCCTGCAGGACCGGCTCGGGCTTGCCAGACCGGTCGGCCCTGCCCCTGTGTGCCCCAACAGGACTCGGTCTCACTACAGGTGCGGCTGGAGGCCTGTACAGGACAGTCTCAGGCTTGCTGGACCGGTCAACCCAACCCCTGCGTGCCCCCACCGGACTCGGCCACCTCCGAGGCTGCGGCTGGAGCATGCTAAAGGAGCCGGTCCTGGTCGCCGGACCGCTCAGACCGGCCCGTGCGGGCCCGCACAGCACTCGGTCACACTAAAGCCGCAGCTGGAGGCCTCTGCAGGACCGGCTCGGGCTTGCCAGACCGGTCGGCCCAGCCCCTGCGTGCCCCACAGGACTCGGTTTCATTGCAGGTGCGGCTGCAGGCCTCTGCAGGACCGGCTCGGGCTCTCCGGAACGGTCAGCCCGGCCCCTGCGTGCCCCCACCCGACTCGGCCACCTCCGCAGCTGTGGCTGGAGCATGCTGAAGGAGCCGGTCCCGGTCGCCGGACCGGTCGGAACGGCCCCTGCGGGCCCGCACTGCACTCGGTCACACTAAAGCCGCAGCTGGCGCCCTCTGCAGGACCGGCTCGGGCTCGCCAGACCGGTCGGACCGGCCCCTGTGGACCCCCACAACACTTGGTCACACTAAAGCCGCAGCTGGAGGCCTCTGCAGGACCGGCTCGGGCTCTCCGGAACAGTCAGCCCGGCCCCTGCGTGCCCAAACAGGACTCGGTCTCACTGCAGGTGCGGCTGGTGGCCTGTACAGGACCAGCTCAGGCTCGCCGGACCGGTCACCCGGACCCCTGCGTGCCCCCACCGGACTCGGCCTCCTCCGCAGCTGTGGCTGGAGCATGCTGAAGGAGCCGGTCCTGGTCGCCGGACCGCTCAGACCGGCCCGTGCGGGCCCGCACAGCACTCGGTCACACTAAAGCCGCAGCTGGCTCCCTCTGCACGACCGGCTCGGGCACGCCAGACCGGTCGGACCGGCCCCTGGGGACCCCCACAACACTTGGTCACACTAAAGCCGCAGCTGGAGGCCTCTGCAGGACCGGCTCGGGCTCTCCGGAACTGTCGGCCCGGCCCCTGTGTGCCCAACAGGACTCGGTCTCACTGCAGGTGCGGCTGGTGGCCTGTACAGGACCAGCTCAGGCTCGCCGGACTGGTCACCCGGACCCCTGCGTGCCCCCACCGGACTCGGCCTCCTCCGCAGCTGTGGCTGGAGCATGCTGAAGGAGCCGGTCCCGGTCGCCGGACCGCTCAGACCGGCCCGTGCGGGCCTGCACAGCACTCGGTCACACTAAAGCCGCAGCGGGCTCCCTCTGCACGACCGGCTCGGGCTCGCCAGACCGGTCAGACCGGCCCCTGGGGACCCCCACTACACTTGGTCACACTAAAGCCGCAGCTGGAGGCCTGTGCAGGACCGGCTCGGGCTTGCCAGACCGGTCGGCCCAGCCCCTGCGTGCCCCACAGGACTCGGTTTCATTGCAGGTGCGGCTGCAGGCCTCTGCAGGACCGGCTCGGGCTCTCCGGAACGGTCAGCCCAGCCCCTGCGTGCTTCCACCCGACTCGGCCTCCTCCGCAGCTGCGGCTGGAGCATGCTGAAGGAGCCGGTCCCGGTCGCCGGACCGCTCGGACCGGCCCCTGCGGGCCCGCACAGCACTCGGTCACACTAAAGCCGCAGCTGGCGCCCTCTGCAGGACCGGCTCGGGCTCGCCAGACCGGTCGGACCGGCCCCTGGGGACCCCCACAACACTTGGTCACACTAAAGCCGCAGCTGGAGGCCTCTGCAGGACCGGATCGGGCCCTCCGGAATGGTCGGCCTGGCCCTTGTGTGCCCCACAGGACTCGGTCTCACTGCAGGTGCGGCTGGTGGCCTCTGCAGGACCGGCTCGGGCCCTCCGGAATGGTCGGCCTGGCCCCTGTGTGCCCCACAGGACTCGGTCTCACTGCAGGTGCGGCTGGTGGCCTGTACAGGACCAGCTCAGGCTCGCCGGACTGGTCACCCGGACCCCTGCGTGCCCCCACCGGACTCGGCCTCCTCCGCAGCTGTGGCTGGAGCATGCTGAAGGAGCCGGTCCCGGTCGCCGGACCGCTCAGACCGGCCCGTGCGGGCCCGCACAGCACTCGGTCACACTAAAGCCGCAGCTGGCTCCCTCTGCAGGACCGGCTCGGGCTCGCCAGACCGGTCGGACCGGCCCATGGGGACCCCCACAACACTTGGTCACACTAAAGCCGCAGCTGGAGGCCTCTGCAGGACCGGCTCGGGCTCTCCGGAACTGTCGGCACGGCCCCTGTGTGCCCCACAGGACTCGGTCTCACTGCAGGTGCGGCTGGTGGCCTCTGCAGGACCGGCTCGGGCCCTCCGGAATGGTCGGCCTGGCCCCTGTGTGCCCCACAGGACTCGGTCTCACTGCAGGTGCGGCTGGTGGCCTGTACAGGACCAGCTCAGGCTCGCCGGACTGGTCACCCGGACCCCTGCGTGCCCCCACCGGACTCGGCCTCCTCCGCAGCTGCGGCTGGAGCATGCTGAAGGAGCCGGTCCCGGTCGCCGGACCGCTCAGACCAGCCCGTGCGGGCCCGCACAGCACTCGGTCACACTAAAGCCGCAGCTGGCTCCCTCTGCACGACCGGCTCGGGCACGCCAGACCGGTCAGACCGGCCCCTGGGGACCCCCACAACACTTGGTCACACTAAAGCCGCAGCTGGAGGCCTCTGCAGGACCGGCTCGGGCTTGCCAGACCGGTCAGCCCAGCCCCTGCGTGCCCCACAGGACTCGGTCTCACTGCAGGTGCGGCTGCAGGCCTCTGCAGGACCGGCTCGGGCTCTCCGGAACGGTCAGCCCGGCCCCTGCGTGCCCCCACCCGACTCGGCCTCCTCCGCAGCTGCGGCTGGAGCATGCTGAAGGAGCCGGTCCCGGTCGCCGGACCGCTCGGACCGGCCCCTGCGGGCCCGCACAGCACTCGGTCACACTAAAGCCGCAGCTGGCGCCCTCTGCAGGACCGGCTCGGGCTCGCCAGACCGGTCGGACCGGCCCCTGGGGACCCCCACAACACTTGGTCACACTAAAGCCGCAGCTGGAGGCCTCTGCAGGACCGGCTCGGGCTCTCCGGAACTGTCGGCCCGGCCCCTGTGTGCCCCACAGGACTCGGTCTCACTGCAGGTGCGGCTGGTGGCCTCTGCAGGACCGGCTCGGGCCCTCCGGAATGGTCGGCCTGGCCCCTGTGTGCCCCACAGGACTCGGCCTCCTCCGCAGCTGTGGCTGGAGCATGCTGAAGGAGCCGGTCCCGGTCGCCGGACCGCTCAGACCGGCCCGTGCGGGCCCGCACAGCACTCGGTCACACTAAAGCCGCAGCGGGCTCCCTCTGCACGACCGGCTCGGGCTCGCCAGACCGGTCAGACCGGCCCCTGGGGACCCCCACAACACTTGGTCACACTAAAGCCGCAGCTGGAGGCCTCTACAGGACCGGCTCGGGCTCTCCGGAACTGTCGGCCCGGCCCCTGTGTGCCCCACAGGACTCGGTCTCACTGCAGGTGCGGCTGGTGGCCTGTACAGGACCAGCTCAGGCTCGCCGGACTGGTCACCCGGACCCCTGCGTGCCCCCACCGGACTCGGCCTCCTCCGCAGCTGTGGCTGGAGCATGCTGAAGGAGCCGGTCCCGGTCGCCGGACCGCTCAGACCGGCCCGTGCGGGCCCGCACAGCACTCGGTCACACTAAAGCCGCAGCGGGCTCCCTCTGCACGACCGGCTCGGGCTCGCCAGACCGGTCAGACCGGCCCCTGGGGACCCCCACTACACTTGGTCACACTAAAGCCGCAGCTGGAGGCCTCTGCAGGACCGGCTCGGGCTTGCCAGACCGGTCGGCCCAGCCCCTGCGTGCCCCACAGGACTCGGTTTCATTGCAGGTGCGGCTGCAGGCCTCTGCAGGACCGGCTCGGGCTCTCCGGAACGGTCAGCCCAGCCCCTGCGTGCCCCCACCCGACTCGGCCTCCTCCGCAGCTGCGGCTGGAGCATGCTGAAGGAGCCGGTCCCGGTCGCCGGACCGCTCGGACCGGCCCCTGCGGGCCCGCACAGCACTCGGTCACACTAAAGCCGCAGCTGGCGCCCTCTGCAGGACCGGCTCGGGCTCGCCAGACCGGTCGGACCGGCCCCTGGGGACCCCCACAACACTTGGTCACACTAAAGCCGCAGCTGGAGGCCTCTGCAGGACCGGCTCGGGCTCTCCGGAACTGTCGGCCCGGCCCCTGTGTGCCCCACAGGACTCGGTCTCACTGCAGGTGCGGCTGGTGGCCTCTGCAGGACCGGCTCGGGCTCTCCGGAACGGTCAGCCCGGCCCCTGCGTGCCCCCACCCGACTCGGCCTCCTCCGCAGCTGCGGCTGGAGCATGCTGAAGGAGCCGGTCCCGGTCGCCGGACCGCTCGGACCGGCCCCTGCGGGCCCGCACAGCACTCGGTCACACTAAAGCCGCAGCTGGCGCCCTCTGTAGGACCGGCTCGGGCTCGCCAGACCGGTCGGACCGGCCCCTGGGGACCCCCACAACACTTGCTCACACTAAAGCCGCAGCTGGCGCCCTCTGCAGGACCGGCTCGGGCTCTCCGGAGCGGTCAGCCCGGCCCCTGCGTGCCCAAACAGGACTCGGTCACACAGCAGGTGCGGCTGGTGGCCTGTACAGGACCAGCTCAGGCTCGCCGGACTGGTCACCCGGACCCCTGCGTGCCCCCACCGGACTCGGCCTCCTCCGCAGCTGTGGCTGGAGCATGCTGAAGGAGCCGGTCCTGGTCGCCGGACCGCTCAGACCGGCCCGTGCGGGCCCGCACAGCACTCGGTCACACTAAAGCCGCAGCTGGCTCCCTCTGCACGACCGGCTCGGGCACGCCAGACCGGTCAGACCGGCCCCTGGGGACCCCCACAACACTTGGTCACACTAAAGCCGCAGCTGGAGGCCTCTGCAGGACCGGCTCGGGCTTGCCAGACCGGTCGGCCCAGCCCCTGCGTGCCCCACAGGACTCGGTTTCATTGCAGGTGCGGCTGCAGGCCTCTGCAGGACCGGCTCGGGCTCTCCGGAACGGTCAGCCCAGCCCCTGCGTGCCCCCACCCGACTCGGCCTCCTCCGCAGCTGCGGCTGGAGCATGCTGAAGGAGCCGGTCCCGGTCGCCGGACCGCTCGGACCGGCCCCTGCGGGCCCGCACAGCACTCGGTCACACTAAAGCCGCAGCTGGCGCCCTCTGCAGGACCGGCTCGGGCTCGCCAGACCGGTCGGACCGGCCCCTGGGGACCCCCACAACACTTGGTCACACTAAAGCCGCAGCTGGAGGCCTCTGCAGGACCGGCTCGGGCTCTCCGGAACTGTCGGCCCGGTCCCTGTGTGCCCCACAGGACTCGGTCTCACTGCAGGTGCGGCTGGTGGCCTCTGCAGGACCGGCTCGGGCCCTCCGGAATGGTCGGCCTGGCCCCTGTGTGCCCCACAGGACTCGGTCTCACTGCAGGTGCGGCTGGTGGCCTGTACAGGACCAGCTCAGGCTCGCCGGACTGGTCACCCGGACCCCTGCGTGCCCCCACCGGACTCGGCCTCCTCCGCAGCTGTGGCTGGAGCATGCTGAAGGAGCCGGTCCCGGTCGCCGGACCGCTCAGACCGGCCCGTGCGGGCCCGCACAGCACTCGGTCACACTAAAGCCGCAGCGGGCTCCCTCTGCACGACCGGCTCGGGCTCGCCAGACCGGTCAGACCGGCCCCTGGGGACCCCCACTACACTTGGTCACACTAAAGCCGCAGCTGGAGGCCTCTGCAGGACCGGCTCGGGCTTGCCAGACCGGTCGGCCCAGCCCCTGCGTGCCCCACAGGACTCGGTTTCATTGCAGGTGCGGCTGCAGGCCTCTGCAGGACCGGCTCGGGCTCTCCGGAACGGTCAGCCCGGCCCCTGCGTGCCCCCACCCGACTCGGCCACCTCCGCAGCTGCGGCTGGAGCATGCTGAAGGAGCCGGTCCCGGTCGCCGGACCGCTCAGACCGGCCCGTGCGGGCCCGCACAGCACTCGGTCACACTAAAGCCGCAGCTGGCTCCCTCTGCACGACCGGCTCGGGCTCGCCAGACCGGTCAGACCGGCCCCTGGGGACCCCCACTACACTTGGTCACACTAAAGCCTCAGCTGGAGGCCTCTGCAAGACCGTCTCGGGCTTGCCAGACCGGTCGGCCCGGCCCCTGTGTGCCCCAGCAGGACTCGGTCAAACTGCAGCAGTGGCTGCAGGCCTCTGCAGGACCGGCTCGTGCCCCCCTGAACGGTCAGCCCGACGCCTGCGTGCCCCAACAGGACTCGGTCACACAGCAGTAGTGGCTGGAGGCCTGTGCAGGACCGGCTCGGGCTCGCCGTACCAGTCGGCCCAGCCCCTGTGTGCCCCGCAGGACTTGGTCTCACTGCCGGTTCGGCTGGTGGCCTGTACAGGACCGGCTCAGGCTTTCCTTACCGGTCACCCTGACCCCTGCGTGCCCCCACCCGACTCGGCCACCTCCGCAGCTGTGGCTGGAGCATGCTGAAGGAGTTGGTCCCCATCGCCGGACCGCTCGGACCGGCCCCTGCGGGCCCGCACAGCACTCGGGCACACTAAAACCGCAGCTGGTGCCCTCTGCAGGACCGTCTCGGGCTCGCCAGACCGGTCGGACCGGCCCCTGGGGACCCCCACAACGCTCTGTCACACTAAAGCCGCAGCTGGAGGCCTCTGCAAGACCGGCTCGGGCTTGCCAGACCGGTCAGCCCGGCCTTTGCGTGCCCCAACAGGACTCGGTCAAACTGCAGCAGTGGCTGGAGGCCTCTGCTGGACTGGCTCCGGCTCTCTGGAACGGTCAGCCCGGCCCCTGTGTGCCCCCACAGGACTAGGTCTCACTGCAGGTGCGGCTGCAGGCCTCTGCAGGACCGGCTCGGGCTCGCCGGACCGGTCGGCCCGGCCCCTGCATGCCCCAACAGGACTCGGTCACACTGCAGCCGTGGCTGGAGGCCTACGCAGGACCGGCTCGGACCCTCCGGACCGGTCGGCCCGGCCCTTGCAGGCCCCGAAAAAAATCAGTCTCATGGCAGCCGCAGCTGGAGATCGCTGCCGGGGCGTCTCGTGTACTCCTACACGGTCTACTTGGCCCCGACCGACTCGCACACTCCGCGGCGGCAGCTCGATGTCTCTGCCGGGGCGGCTCGAAAACGGCGGAACGGTCTACCAGGCCCCGGCAGACTTCCTCCGGCTCGGTCGCTCCGCGGCGGCGGCTAGAGGTCGCTGCCGGGGCGGCTGGGTCACGGCGGAACGGTCTACCCGGCCCCGGAGGGTTCCGAGCCGCTTCACCTGTTGTGGCCGCTAGAGGTCGCTGCCGCGTAAATGTCGGGTACGCCGGCCACGTCTACCCGGCCAGGACGGACTTGGTCGCTTCGCGGCGGCGGCTGGAGGTTGCTGCGGGAGCCGCTCCCGGTCGCCGGACCGGTCGGCCCGGCCCCTGCGGGCGCCGAGCGACACGGTCGTTCGGCGGCGGCGGCTGGGTACCGTGGCAGGATCGGCTCGGGTTCGCCGGACCGGTGGTCCCGGCCCGACGGGCACGGGCCACTCGGCCGTTCCGCGGCAATGGGTGGAGACTCGTGTAAGACCGTCTCACGTTCGCCGGATCGGTCGGCCAGGCCCCGTCCGACTCGGTCGCTTCGCGGAGGTAGCTGGAGGTCGCTGCCGTGGCGGCTGGGGCACGGCGGGACGGTCTACCTTGCCCCGGCAGGCTGCGTCCGGCTCGGTCGCTCCGCGGAGGAGGCTAGGGGTCGCTGCCGGGGCGGTTCGGAAACGGCGGGACGGTCTACCCGGCTCCGGCGGTCCCCGTCCGGCTCGGTCTCTCCGCGGCGGCGTCTAGGGGTCGCTGCCGGGGCGTCTCGGAAGCGGCGGGACGGTCTACCGGGCTCCGGCAGGCTTCGTCCGGCTCGGTCGCTCCGCGGCGGCGGCTAGAGGTCGCTGCCGGGGCGGCTGGGTCACGGCGGAACGGTCTACCCCGCCCCGGAGGGTTCCGAGCCGCTTCACCTGTTGTGGCCGCTAGAGGTCGCTGCCGCGTAAATGTCGGGTACGCCGGCCACGTCTACCCGGCCAGGACGGACTTGGTCGCTTCGCGGCGGCGGCTGGAGGTTGCTGCGGGAGCCGCTCCCGGTCGCCGGACCGGTCGGCCCGGCCCCTGCGGGCGCCGAGCGACACGGTCGTTCGGCGGCGGCGGCTGGGGACCGTGGCAGGAGCGGCTCGGGCTCGCCGGACCGGTGGTCCCGGCCCCGTCCGACTCGGTCGCTTCGCGGAGGTGGCTGGAGGTCGCTGCCGTGGCGGCTGGGGCACGGCGGAACGGTATACCCGGCTCCGGCGGGCTGCGTCCGCCTCGGTCGCTGCCGTGGCGGCTGCTAGGGGTCGCTGCCGGGGTGGCTGGGGCACGGCGGAACGGTCTACCCGGCCCCGGCGGGCCCCGTCCGCCTCGGTCGCTCCGCGGAGGAGGCTAGGGGTCGCTGCCGGGGCGTCTCGGAAACGGCGGGACGGTCTACCTTGCCCCGGCAGGCTTCGTCCGGCTCGGTCGCTCCGCGGCGGCGGCTAGAGGTCGCTGCCGTGTCGGCTCGGAAACGGCGGAACGGTCTACCCGGCCCCGGCAGGCTGCGTCCGGCTCGGTCGCTCCGCGGCGGCGGCTAGAGGTCGCTGCCGGGGCGGCTGGGAAACGGCGGGACGGTCTACCCGGCCCCGGCGGTCCCCGTCCGACTTGGTCGCTCCGCGGCGGCGGCTAGAGGTCGCTGCCGGGGCGTCTCGGAAACGGCGGGACGGTCTACCTTGCCCCGGCAGGCCTCGTCCGGCTCGGTCGCTCCGCGGCGGCGGCTAGGGGTCGCTGCCGTGTCGGCTCGGAAACGGCGGAACGGTCTACCCGGCCCCGGCAGGCTGCGTCCGGCTCGGTCGCTCCGCGGCGGCGGCTAGAGGTCGCTGCCGGGGCGGCTGGGAAACGGCGGGACGGTCTACCCGGCTCCGGCGGGCCCCGTCCGACTTGGTCGCTCCGCGGAGGAGGCTAGGGGTCGCTGCCGGGGCGTCTCGGAAACGGCGGGACGGTCTACCTTGCCCCGGCAGGTTTCGTCCGGCTCGGTCTCTCCGCGGCGGCGGCGGCTAGGGGTCGCTGCCGGGGCGGCTCGGAAACGCCGGCACGGTCTACCTGGCTCCGGCGGTCCCCGTCCGGCTCGGTCGCTCCGCGGCGGCGGCTAGGGGTCGCTGCCGGGGCGGCTGGGGCACGGCGGAACGGTCTACCTGGTCCCGGCGGGCACCGTCCGGCTCGGTCTCTCCGCGGCGGCGGCGGCTAGGGGTCGCTGCCGGGGCGTCTCGGAAACGGCGGAACGGTCTACCCGGGTGCTACCGTCTCGCGCTCTCCGCGGCGGCGGCTAGAGGTCGCTGCCGGGGCGGCTTGCGATCCGCGTCCAGGTCTACCCCGTTTCGGATTGTCTTGGCCGCTCTGGCTGTGGGGGGGGGCGCTACAGCTCCGGAGCTGCCAGAGGCGTCGCTGTAATTTTGTACCTCCAGTTACGTCGAGGTAAACCTCGGCTGCCGTCGGAGCCGCTGCCGGTAGTCGGCGCCTATGGGACTAGAACGTTTTTTTCGGATGCCTTATATGTTCGTCTGTAGGAGCGAGTGAGGACTCGGCTCCGGTAGTGGCGGTGAGCGGGCGCTCGCGAGCAGGGTTGACCGGCCGGCCGCCTAGAGAGGGGATCGGCGGCGGCGGCGGCGGCTTTCTCGGGCATCGGTTCGTTCGATCGGTCCGGTCGCTTCGGTTTGTCCGTCGCTCCTCATCCCGCAGCTCTGTCCTGGGCTAAGGCGGTTTTGCAGGCGAGCAGCGAAAAAAAGCCGGAGAAGGCGAGAGAGAGGCAAGAAGCAAGCCGGCTCCCGCGCCGCCAGGGCGAAGGCGAGAGAGAGAGGGAGACGAGAAGGGCACGGGCCGGTCTGCCGGCACCCGAACGTAGGATGGCCGGGGGCGTCCCCGGCGGGTCCCGCCGCGATGGAAGAGGGGGACCCGGAGGTCGTAGGTCGTGGCGGCGTCGCCTCGTCCTCCTTTCGCACCGCATTCTCACCCGCACGCGGGAGCCCCGGCCGATTCGTGGCGCTCCTCGGGCGCGTCGGGGAGGCTTCCCGGCGGGCCGGCTCTATCCCGCTCCCCGGCTCGTTCGGGGTGGCGTGGGGCGGGCCGGTGTTCAGGCACGGGCGAGCACCTCTCGTCGGACGTTGCCCACGCACACCCACCTGCACGTGCGCGTGCGGTCTTTCCGCCGCGCCTGGGGGAAGGGCTCGCGCCTTCTCCCTCCTTTCTTTCTCCTCCCCCCCACCCCCTTTCTCCCACCGATCGATGAGGCCACTCGGGTCGCGTCGGAGAGGGCCCCCGGCGGGCCGGCGCTCTGCGCTCCCTGTCCCAGGGAAGCCGCGGCGGCGTCCGGTGTTCAGGCACGGGCGGCCTCCTCTCCAGTTCGCTTCCCGTCGTTCGCGAGGTGAGGCGCTCGCCCGCTTGGGCCGAGGGCGGCGGCGGCGGCGGCTTCGGGGCGCGTGGCCTCGCCGTGCCGACTCGTCTGTCCGCCCGCCCTGTCGGTGCCCCAGGGCTCGCCCGACCGAATCCAGCTGTGTGACGGCCGAGCGGCCCCGCGAGCCGCAGGCGTACCTATTTCGTTGTGAGCGAGGCGTCGGCGCTGCCCTCGTTTCGGGGCCCGGCGAGTGCCGGCCGCGAGCAGCAAGCCGGCGGGGTGGCAACCGAGGGAAACCGCGGGGAACCGAGGCGAAGCGAGCAGCAGCAGAAGAAGAAGGAACGAGAAGACAACGGGGGGCTGCGCCCGGCCGAGCGGGCGAGCCCGGAGCAGCGCGGCGCGTCCCGCTCCGGATCCGTCGGGGTGTGGGGGCCGGGGGCGTCCGCCGGCCTCTCCTCCGCCTTCGGGCCGCCGCAGCCCGTGTCGGTTTTCCTGCCGCGTCCCCGCCCGCTGCGGAGCGTGCCGCCCCGGGAAAGGGTCTCCGATCGTGGGGTCGCGCCCGTCTCGAGGTCGCGTTCTCCTCTAGCACGTCCGTCTCCGGCGGCGGGGCTTCGTTTCCCCGTCCGCTTCTCCGCCGGTCCCGGAGGGCGGGTCAGCCCCGGCCGGCCGTGCGGCGCGAGCGCGAGTCCGGCTCCCGCGGGGGGGGCCCGGAGCGTGCCGCCGAAAGCAGCTGCGCAGCGGTCCCCGCTCCTTCCCCGCGGGGGGGAGGTCGGCGGGGCCGCCCCGGGGATCGGGCGCGCCTCTCCGTCGTGGTCGGCGAGCGAGCGAGCGAGCGAGGGAACGACGGAGGGCCGCCCGCCCCGCCGAGAGGCGTTCGCCCCGGCGGCCGCCGCCGTCGACCCGGCAAGGGCCAGACGGGAAAGCCGAGCGAGCAGGCGAGAGAGAGAGAGAGGGAAGGAGCGAGAGCGGTCGGCGGCGGGCCGGGCCCGTCGGGTCGTGCCCCGTGGCGCGGCTACCTGGTTGATCCTGCCAGTAGCATATGCTTGTCTCAAAGATTAAGCCATGCATGTCTAAGTACACACGGGCGGTACAGTGAAACTGCGAATGGCTCATTAAATCAGTTATGGTTCCTTTGGTCGCTCCCCTCCCGTTACTTGGATAACTGTGGTAATTCTAGAGCTAATACATGCCGACGAGCGCCGACCTCCGGGGACGCGTGCATTTATCAGACCAAAACCAACCCGGGCTCGCCCGGCGGCTTTGGTGACTCTAGATAACCTCGAGCCGATCGCACGCCCCCGTGGCGGCGACGACCCATTCGAATGTCTGCCCTATCAACTTTCGATGGTACTGTCTGTGCCTACCATGGTGACCACGGGTAACGGGGAATCAGGGTTCGATTCCGGAGAGGGAGCCTGAGAAACGGCTACCACATCCAAGGAAGGCAGCAGGCGCGCAAATTACCCACTCCCGACCCGGGGAGGTAGTGACGAAAAATAACAATACAGGACTCTTTCGAGGCCCTGTAATTGGAATGAGTCCACTTTAAATCCTTTAACGAGGATCCATTGGAGGGCAAGTCTGGTGCCAGCAGCCGCGGTAATTCCAGCTCCAATAGCGTATATTAAAGTTGCTGCAGTTAAAAAGCTCGTAGTTGGATCTTGGGATCGAGCTGGCGGTCCGCCGCGAGGCGAGCTACCGCCTGTCCCAGCCCCTGTCTCTCGGCGCCCCCTCGATGCTCTTAACTGAGTGTCCCGCGGGGCCCGAAGCGTTTACTTTGAAAAAATTAGAGTGTTCAAAGCAGGCTGGCCGCCGGAATACTCCAGCTAGGAATAATGGAATAGGACTCCGGTTCTATTTTGTTGGTTTTCGGAAACGGGGCCATGATTAAGAGGGACGGCCGGGGGCATTCGTATTGTGCCGCTAGAGGTGAAATTCTTGGACCGGCGCAAGACGAACTAAAGCGAAAGCATTTGCCAAGAATGTTTTCATTAATCAAGAACGAAAGTCGGAGGTTCGAAGACGATCAGATACCGTCGTAGTTCCGACCATAAACGATGCCGACTCGCGATCCGGCGGCGTTATTCCCATGACCCGCCGGGCAGCTCCCGGGAAACCCAAGTCTTTGGGTTCCGGGGGGAGTATGGTTGCAAAGCTGAAACTTAAAGGAATTGACGGAAGGGCACCACCAGGAGTGGAGCCTGCGGCTTAATTTGACTCAACACGGGAAACCTCACCCGGCCCGGACACGGACAGGATTGACAGATTGAGAGCTCTTTCTCGATTCCGTGGGTGGTGGTGCATGGCCGTTCTTAGTTGGTGGAGCGATTTGTCTGGTTAATTCCGATAACGAACGAGACTCTGGCATGCTAACTAGTTACGCGACCCCCGAGCGGTCGGCGTCCAACTTCTTAGAGGGACAAGTGGCGTTCAGCCACCCGAGATTGAGCAATAACAGGTCTGTGATGCCCTTAGATGTCCGGGGCTGCACGCGCGCTACACTGACTGGCTCAGCTTGTGTCTACCCTACGCCGGCAGGCGCGGGTAACCCGTTGAACCCCATTCGTGATGGGGATCGGGGATTGCAATTATTCCCCATGAACGAGGAATTCCCAGTAAGTGCGGGTCATAAGCTCGCGTTGATTAAGTCCCTGCCCTTTGTACACACCGCCCGTCGCTACTACCGATTGGATGGTTTAGTGAGGTCCTCGGATCGGCCCCGGCGGGGTCGGCCACGGCCCTGCCGGAGCGTCGAGAAGACGGTCGAACTTGACTATCTAGAGGAAGTAAAAGTCGTAACAAGGTTTCCGTAGGTGAACCTGCGGAAGGATCATTACCGGGGCCGAGGCCGGGCGTCCGGCCGAGCCGTGGCACGAGCGCGCGCGGGCGCGCAGCCTTCCCTTCCCTTCCCCGAGCCCGCTCCGCGCGGAGCGCGGCTCCTCTCCCCCGGTCGAAACGGGGAAAGAAAAAAAAAACACCGCAAGTCGCTCCGCGCGCCTGCCGGCGAGAGAGAAGGGAGACGAGGGCGCGGAGCGCAGCTCCGGGGGGGAGGCGCGTGTGGGGCGCTCCGGCGCTCCGGCGCGTCTCTCCCCCCCGGCGCCGGTCCGCCGTCGGTCCGCACGCCGCGGGTCCGGTCCGTCCGGTCGCCTCGCCGGCGCGCGCCCGCGCGCGCGCGTCCCGCGGGCCTCGCCCGGGTCGCCGCGCTCCGGAGCGTCCCGCGGCCGAGTCCCGCTCCGACCGCGGGGTCGGGGTCGGGAGGTGGCGGCGGTGCGGAGGGTGGAAGGACGGCTCCCCGCTTCGTCGCTCGGCCGGAAACTCGCCACCGGCCCCCGCCGCTGTCGACGCCGGCACCCCGAGTCCGCTCGGAGGGAAGCCGCGCGGGCGGCCGCGCGCGGGGGAGGCGGCGGGCGGCGGGTCCGAGCGCGGGGCGCGGGAAGTCGGCCGCTTCCCCCGGCCTCACCCCCCACCCCCTTCGCCCGGCCCGTCGCGGGGACGGGGCCGGGTCGCGGGCGGCTGCGGAGCCGGCCGACTCCGGGCGAGCGCCGGAGGGACGCGCGCGCCGCGTACGCGCGGCAGGCGCGAGGTGCCCCGGGCGGCTTCGGTCCCGCGCGGGCGGTCCGAGCCTCGCGGCTCCTCCCGGGTGCAGCTGCCGCCCGGCGCCGGGTTGCCGAGGGAAACCCCGGGCCCCGGGAGGAACGCGAGGTGGTGGCGGCGGACGTCGGGCGCGCCCCCGCGGGCGGACGCTCCCCCGAGGGGCGCCGGGGCCGGCTGGCGGGTGCCGGGTCTCCCCTCGGCGCCCCGTCCCGCCCCGCCGAGCGGGGCGGGCGGGGGAGGCACCCCCGCGGGGCCTTCGGGTCGTTTCCCTCACCCCAGGGCCAGGTACCTAGCGTCCGCGCCTCCGCGCGTCCGGGGGGCGGGGAGGAAGGAGCGCGGCGCCGGTCCCGAGCGGGCCGCGTCGCCCACACCCCCCTCCTCCCCCCGGGCCGCGGAGCCGGGCGGAGGTTTAAAGACTCGGGCGGCCCGCGGCGCGCGCCGCGAGGTCGGGGGCCGGGGGCGGTCTTCTGCCCGCCGGCGGGACGCCGGGATGGAAGAGAGGAGTCCGGGCGGGGCGCGGCGGCGCGCCCCGCCGGCCCTCTCCCTCCCGAGCCCGCCGGCGGCGTCGGCCGTCGCCGCGCCCTCGGTCCTCCGCGGGGCGGGCCCGGGCCGGAGAGGGGGTCATCCCGTCCCCCCTCTCCGCGGCCTCGGTCTCGGGCGGAGAGCTCGGCGCGCGCGCGGGCGCGCGCTCGCTCCGGCCGGCCTCGCCCGCGTACGGAGCGGGCCGAGACGCGGGTCTCGGCCCGGCGCCCGCCGCTCCCCGCGGCGGTGCGTTCCGCGGCCTCCCCGCCGCGCGCGGCCGGCGGGACGGCGAGCCGGCCGTCCCGCCCGCGCCAGCCGCGGCGCCGGCGGTTCCGCTCCGCCGGTCCGCCCGGCGTGCGTCCGCACGCCCGGCCTCCTGCCCTCCCTCGGGGCCTCGCCGCCGTTTCCCCCTTCCGTCGCAAGCCGCGTCCTCTCCTTCGTCCCCGCCGCCGTCGCCTCCCACCGCGCTTTCGCCCTCGGCCTCGCCGGCCGCGCCGGTCGTGCGAGCGGGAGGTCCGGCGTGGGGCGTCCCGCAGCCGGTCTCCGCGCGGAGGCGCGGGGAGCGGGCGCCGCTCCCGAATCCGTCCCCGTCCCGCCCGCCGCCGTGCGCGCGTCCGCCGCGGGCGCGCCGCCAGGGCGAGCGAGAGGAGGAGGCGTCGGAGGACGAGGGGCGGGGGAGGAAGGTGAGAGGCGGCGGGGGCGTTTCGGTGCGCGCGTCTCCCGCACGGCGAGGAAGGGGCCGAGGTCGGCGCGGGCGCCGTCGGGCGGTCCGTCGCGGCAGCGGGGCTTCGGCCGGGGCGGCGCGCGCCGTCCCGCGGGCGTCCGCGGCTCCTCCGCCCGGGCCGGGCCGAGCCGGGCGCCTGGTCCGTCCCCGAAGCGAGACAGGGTCGTTTCCCCAGGTCGGGAGCGAGGGCTCCCCGCCCTTCTCGTTCGGGTCGCGCTTCATTGCCGGCCGGCCGGCCGGCCGGCCGTCGCCGGCTTTTTTTTTCCCTCCCGCATCCGATATTCGTGTGCTCGTACGGTCAGCGGAGGCGACGCTCGTCCGCCCCGCGGTCGCCCCGGCGTCGGGGCTGGCCGCGGGCGCGGGCCGAGCGCCTTCGGGCAAGGCGAGAGAGAACGAGAGCGGTCCCCCCGCGCGCGCGGGGCGGTGCCGAAAGTCAGACAACTCTTAGCGGTGGATCACTCGGCTCGTGCGTCGATGAAGAACGCAGCTAGCTGCGAGAATTAATGTGAATTGCAGGACACATTGATCATCGACACTTCGAACGCACTTGCGGCCCCGGGTTCCTCCCGGGGCTACGCCTGCCTGAGCGTCGCTTGACGGTCAATCGCCGACGGCCGCCGTCCGCGGCGGCCGCGCGGCGCGGCTGGGGCGCCTCGCAGGCCCGCGCGCCCCGCCGGAGGCGGGTCGCGAGGGGGGGGCCGCCGTCCGTCCGTCCGTCCGCCCGTCGGTCGGTCGGTTCGGGCGCCCGGATTCCCTCCCCCGCACCCCCTCCGAGCGGCGTCGCGCCGCGGGCCTTCGTCCCCCTAAGTGGAGACCCAGGTCGGGGAGCTCGCCGAGCTCCCCGCGCTCCCGGAGCGCCCGCTTTGGCCGAGCTCGTCCCCACGGGGCGGCCGGGCTTTCCGGTCGGTCGCGCGGCGCAGCGCGGCGGGGCCGGACGTTCGTTCGTTCGTTCGTTCGTCCGGCCCCCCGCCCCGGAGGAGCGCACCTCGCCCTCCCCGGCCCCGCGCGCGGCTGCCTGCGGGTCGCGTTACCGGCGGCGGTAACGCGCCGTGCTGCCGCGCGCGTGGCGGTCCGGGTCGGGGCGAGGCTGCCGGCCTCCGGTCGTCCGCCCGTCCGTCCGGCCGAGCCCGGCGCGCGTCCCCGCGGGTCCGTCTCCGGCCACCGTGCGCCGGCGGCGGCGGCGGCGGCGGTGCGAACCGCCGGCGGCGCGCCGGCTCCCCCGTCCGGGCGTTCCTCCCTCGGCAGCGCCGGGAGCAGCCGCTTGGCGTCCGAAGGCGGGTGGCCGGGCGAGCGCGGGCTCGCCCGGGGCCCGGCGTTCGGGCCCCGTTTCCGATCGCGACCTCAGGTCAGACGTGGCGACCCGCTGAATTTAAGCATATTAGTCAGCGGAGGAAAAGAAACTAACGAGGATTCCCTCAGTAACGGCGAGTGAAGAGGGAAGAGCCCAGCGCCGAATCCCCGCCCCGCGGTGGGGCGCGGGAGGTGTGGCGTACGGAAGCCCCCATCCCCGGCGCCGCTCTCGGGGGCCCAAGTCCTTCTGATCGAGGCCCAGCCCGCGGACGGTGTGAGGCCGGTAGCGGCCCCCCGGCGCGCCGGGCCCGGGGCTTCTCGGAGTCGGGTTGCTTGGGAATGCAGCCCAAAGCGGGTGGTAAACTCCATCTAAGGCTAAATACCGGCACGAGACCGATAGCCAACAAGTACCGTAAGGGAAAGTTGAAAAGAACTTTGAAGAGAGAGTTCAAGAGGGCGTGAAACCGTTAAGAGGTAAACGGGTGGGGTCCGCGCAGTCGGCCCGGAGGATTCAACCCGGCGGGCCAAGGTCGGCCGGCGCGGGCGCCGTCGGATCCCCGCCTCCGCCTCCCCTCCGTCCCTCCCCTTCGCCGGGGCGGGGCGGGCCCAGGGGGGGCGGGCGGGCCGGGGACCGCCGCCCGGCCGGCGTCCGGCCCCCGTCGGGCGCATTTCCTCCGCGGCGGTGCGCCGCGACCGGCTCCGGGACGGCTGGGAAGGGCTGCCGGCGGGCAGGTGGCCCGGCGCCGCGCGAGCGGCCGCCGGGTGTTATAGCCGCCGGGCCCGGATCGTCGCCGAATCCCGGGGCCGAGGGAGAGGACCGCCGCCGCGCCCTCCCCCGGAGGGGGCGGCCCCCCGGAGGGCCCCCCGCGGCCGGACCGGCGTCGGGCCGGCCGCGCCGCGCGCGCGTCCGCGCCGCCGCCGTACGCCGCCGCTCGCTCTCTCTCCGTTCCCCGCCCCGGGTCCGTCCCGGGGCGCGGGGGCGGGGGGGGTCGGGTGTCCGGCGCGCGGCTCGGCGCGGCGCCGCGCGTGTGGCGCGCGCCTCCAGCCCGGCGCGGGCGAGGCCGCGGGGGGCGCCGGGGGGAACCTTCCCCCTTCTGTTCGGGCCGCCTCCGTTCCCGCGGGGGCGGCCCGTTCGGGGGACGGGCCCGCCGGCCCCCGGCGCCGCTGTCCGACCGGGGCGGACTGCGCTCAGTGCGCCCCGACCGCGCGGCGCCGCCGGGCCGGGCTCGGGCCACGCCAGGGCGCCCGGGGTCCGCGGCGACGTCGGCTACCCACCCGACCCGTCTTGAAACACGGACCAAGGAGTCTAGCACGCGCGCGAGTCGGCGGCTCGCGCGAAAGCCCGCGGCGCAATGAAGGTGAGGGCCGGCGCGCGCCGGCTGAGGTGGGATCCCGGGGCGGCAGGCCGGAAGGCCCCGGGCGCACCACCGGCCCGTCTCGCCCGCCTCGCCGGGGAGGTGGAGCATGAGCGCGCGTGCTAGGACCCGAAAGATGGTGAACTATGCCTGGGCAGGGCGAAGCCAGAGGAAACTCTGGTGGAGGTCCGTAGCGGTCCTGACGTGCAAATCGGTCGTCCGACCCGGGTATAGGGGCGAAAGACTAATCGAACCATCTAGTAGCTGGTTCCCTCCGAAGTTTCCCTCAGGATAGCTGGCGCTCGGGGCGGCGGTGCAGTTTTACCCGGTAAAGCGAATGATTAGAGGTCTTGGGGCCGAAACGATCTCAACCTATTCTCAAACTTTCAATGGGTAAGACGCCCGGCTCGCTGGCGTGGAGCCGGGCCGTGGAATGCGAGCGCTCAGTGGGCCACTTTTGGTAAGCAGAACTGGCGCTGCGGGATGAACCGAACGCCGGGTTAAGGCGCCCGATGCCGACGCTCATCAGAGCCCAGAAAAGGTGTTGGTTGATCTAGACAGCAGGACGGTGGCCATGGAAGTCGGAACCCGCTAAGGAGTGTGTAACAACTCACCTGCCGAATCAACTAGCCCTGAAAATGGATGGCGCTGGAGCGTCGGGCCCATACCCGGCCGTCGCCGGCGGTGCGGAGCCGCGGGGGCTACGCCGCGACGAGTAGGAGGGCCGCTGCGGTGCGCCTGGAAGCCTGGGGCGCGGGCCCGGGTGGAGCCGCCGCAGGTGCAGATCTTGGTGGTAGTAGCAACTATTCAAACGAGAGCTTTGAAGGCCGAAGTGGAGCAGGGTTCCATGTGAACAGCAGTTGAACATGGGTCAGTCGGTCCTAAGCGATAGGCGAGCGCCGTTCCGAAGGGACGGGCGATGGCCTCCGTTGCCCTCAGCCGATCGAAAGGGAGTCGGGTTCAGATCCCCGAATCCGGAGCGGCGGAGACAGGCGCCGCGAGGCGCCCAGTGCGGTAACGCAAGCGATCCCGGAGAAGCCGGCGGGAGCCCCGGGGAGAGTTCTCTTTTCTTTGTGAAGGGCCGGGCGCCCTGGAACGGGTTCGCCCCGAGAGAGGGGCCCGCGCCTTGGAAAGCGTCGCGGTTCCGGCGGCGTCCGGTGAGCTCTCGCTGGCCCGTGAAAATCCGGGGGAGAGGGTGTAAATCTCGCGCCGGGCCGTACCCATATCCGCAGCAGGTCTCCAAGGTGAACAGCCTCTGGCATGTTGGACCAATGTAGGTAAGGGAAGTCGGCAAGCCGGATCCGTAACTTCGGGATAAGGATTGGCTCTAAGGGCTGGGTCGGTCGGGCTGGGGCGCGAAGCGGGGCTGGGCGCGCGCCGCGGCTGGACGAGGCGCCGCCGCCCCCGCCCCCCCCTTTCCCCGCTCCCGCTCGCCGGGGCGCCGGGGGGGGGGGTCAGCGGGCGGCGCGGCGGCGGCGACTCTGGACGCGCGCCGGGCCCTTCCCGTGGATCGCCCCAGCTGCGGCGGGCGCCGCTCGCCCCCCTCCTTGCCCTCCGCCCCCCGCTCCCGGCGCCCCTCCCGTCGGCCGTCGTCCGGCCGCCCCCCGTCCCGAGCGCCCTCCCCGCGAGGCGAGGGGCGAGGGGCGGCGGCGGCGGCCGCGGGCGCGGCGGCGGCGGGGGGGGCCCGCCGGCGGCGCCGGGCGGGGCGGTCCCGGGCGGGGGGGGTCTCCGGGCCGGCGCCCCGCCTCGGCCGGCGCCTAGCAGCCGGCTTAGAACTGGTGCGGACCAGGGGAATCCGACTGTTTAATTAAAACAAAGCATCGCGAAGGCCCGCGGCGGGTGTTGACGCGATGTGATTTCTGCCCAGTGCTCTGAATGTCAAAGTGAAAAATTCAATGAAGCGCGGGTAAACGGCGGGAGTAACTATGACTCTCTTAAGGTAGCCAAATGCCTCGTCATCTAATTAGTGACGCGCATGAATGGATGAACGAGATTCCCACTGTCCCTACCTACTCTCCAGCGAAACCACAGCCAAGGGAACGGGCTTGGCGGAATCAGCGGGGAAAGAAGACCCTGTTGAGCTTGACTCTAGTCTGGCGCTGTGAAGAGACATGAGAGGTGTAGAATAAGTGGGAGGCCCCGCGGTCGCGCGACCCGCGCCGCGGCCCGGCCGCCGGTGAAATACCACTACTCTGATCGTTTTTTTCACTTACCCGGTGAGGCGGGGGGGCGAGCCCCGAGGGGCTCTCGCTTCTGGCGCCAAGCGCCCGGCGCGCGCCGGGCGCGACCCGCTCCGGGGACAGCGTCAGGTGGGGAGTTTGACTGGGGCGGTACACCTGTCAAAGCGTAACGCAGGTGTCCTAAGGCGAGCTCAGGGAGGCCAGAAACCTCCCGTGGAGCAGAAGGGCAAAAGCTCGCTTGATCTTGATTTTCAGTACGAATACAGACCGTGAAAGCGGGGCCTCACGATCCTTCTGACTTTTTGGGTTTTAAGCAGGAGGTGTCAGAAAAGTTACCACAGGGATAACTG
